# Design and Engineering of Light‐Induced Base Editors Facilitating Genome Editing with Enhanced Fidelity

**DOI:** 10.1002/advs.202305311

**Published:** 2023-12-01

**Authors:** Yangning Sun, Qi Chen, Yanbing Cheng, Xi Wang, Zixin Deng, Fuling Zhou, Yuhui Sun

**Affiliations:** ^1^ Department of Hematology Zhongnan Hospital of Wuhan University School of Pharmaceutical Sciences Wuhan University Wuhan 430071 China; ^2^ Key Laboratory of Combinatorial Biosynthesis and Drug Discovery (Ministry of Education) Wuhan University Wuhan 430071 China

**Keywords:** base editor, low off‐target effect, optogenetics, spatiotemporal control

## Abstract

Base editors, which enable targeted locus nucleotide conversion in genomic DNA without double‐stranded breaks, have been engineered as powerful tools for biotechnological and clinical applications. However, the application of base editors is limited by their off‐target effects. Continuously expressed deaminases used for gene editing may lead to unwanted base alterations at unpredictable genomic locations. In the present study, blue‐light‐activated base editors (BLBEs) are engineered based on the distinct photoswitches magnets that can switch from a monomer to dimerization state in response to blue light. By fusing the N‐ and C‐termini of split DNA deaminases with photoswitches Magnets, efficient A‐to‐G and C‐to‐T base editing is achieved in response to blue light in prokaryotic and eukaryotic cells. Furthermore, the results showed that BLBEs can realize precise blue light‐induced gene editing across broad genomic loci with low off‐target activity at the DNA‐ and RNA‐level. Collectively, these findings suggest that the optogenetic utilization of base editing and optical base editors may provide powerful tools to promote the development of optogenetic genome engineering.

## Graphical abstract

This study engineered the blue‐light‐activated base editors (BLBEs) by combining the photoswithes magnets with DNA deaminases. The BLBEs system showed a remarkable reduction in background editing and a significant enhancement of blue light‐dependent editing. Simultaneously, the potentially harmful off‐target effects on both the genomic and transcriptomic levels are greatly minimized.

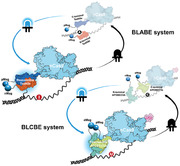

## 1 Introduction

Base editors (BEs) are indispensable tools for gene engineering that can convert A:T to G:C or C:G to T:A in the case of DNA deaminases without introducing double‐stranded DNA breaks (DSBs) or requiring a donor DNA template.^[^ ^1^, ^2^ ^]^ Cas9‐mediated DNA base editors can be divided into cytosine base editor (CBE) and adenine base editor (ABE), which are fused to defective Cas9.^[^ ^3^ ^]^ Because of their capacity to circumvent double‐stranded breaks, which often caused gene damage, BEs have gained considerable attention in genetic research, such as gene therapy^[^ ^4^, ^5^, ^6^, ^7^, ^8^, ^9^ ^]^ and metabolic engineering.^[^ ^10^ ^]^

ABE involves the partnership of dead Cas9 (dCas9) or Cas9 nickase (nCas9) with adenine DNA deaminase, which can cause programmable A‐to‐G transitions in DNA. The initial tRNA‐specific adenine deaminase, TadA, was engineered by directed evolution, leading to the deamination of DNA.^[^ ^2^ ^]^ However, the application of ABE has been limited by the low catalytic efficiency of TadA. To improve the catalytic ability of adenine deaminase, the TadA‐derivative TadA‐7.10 was evaluated by phage‐assisted non‐continuous and continuous evolution, resulting in TadA‐8e,^[^ ^11^ ^]^ which has emerged as a promising enzyme in the application for base editing. The APOBEC3 family enzymes are cytidine deaminases that function as defense tools.^[^ ^12^ ^]^ These enzymes were fused with Cas9 to catalyze cytosine deamination with additional C‐to‐T conversion.^[^ ^1^ ^]^ APOBEC3A (A3A) has been identified as the predominant source of mutations in a broad spectrum of cancer types^[^ ^13^, ^14^ ^]^ and provides the highest deaminase activity in CBE.^[^ ^15^, ^16^, ^17^ ^]^ Although base editing has been widely used in fundamental research and disease therapy, it presents significant off‐target risks, which is also a hurdle in advancing gene editing technology.^[^ ^18^, ^19^ ^]^ Several researchers have reported the malpractices in base editing, including genome‐wide single‐guide RNA (sgRNA)‐dependent or sgRNA‐independent off‐target DNA editing and transcriptome‐wide sgRNA‐independent off‐target RNA editing.^[^ ^20^, ^21^, ^22^, ^23^ ^]^ The sgRNA‐dependent off‐target effects are caused by an sgRNA mismatch or poor target specificity of the Cas nucleases.^[^ ^24^ ^]^ Notably, sgRNA‐independent off‐target effects result from the activities of free deaminases.^[^ ^25^ ^]^

Protein engineering represents a vital means of addressing off‐target problems. Several BE variants have been engineered by introducing additional mutations to alleviate its off‐target effects or improve the specificity of on‐target effects in DNA.^[^ ^26^, ^27^, ^28^, ^29^, ^30^, ^31^, ^32^ ^]^ Furthermore, the Cas9 protein has been engineered into split proteins to control the spatiotemporal editing of BEs, which can significantly reduce sgRNA‐dependent off‐target DNA editing.^[^ ^33^, ^34^, ^35^, ^36^ ^]^ Although these strategies have shown satisfactory performance in improving the specificity of the Cas9 protein, an off‐target risk associated with free deaminases still exists for BEs, indicating that the off‐target situation still needs to be solved.

To date, a few groups used split‐protein methods to control the activity of the cytosine deaminases. These results demonstrate that cytosine deaminase can be split into two independent components combined with chemically triggered elements, resulting in the small‐molecule drug regulation of CBE.^[^ ^37^, ^38^ ^]^ Moreover, narrowing the time window for editing and regulating cytosine deaminase activity can mitigate undesirable effects, such as sgRNA‐dependent and sgRNA‐independent off‐target effects.

Driven by the context of precision medicine, meticulous control and therapeutic manipulation of tissue cells are of paramount significance.^[^ ^39^ ^]^ Optogenetic technologies have emerged as powerful tools that offer robust assistance in engineering genetically modified cells.^[^ ^40^ ^]^ Through the convergence of optogenetics and genome engineering, new avenues have arisen to meticulously govern cellular behavior and advance the treatment of associated pathological conditions. Optogenetic strategies offer significant advantages over traditional chemical induction techniques in terms of spatiotemporal precision, rapid tissue penetration, and flexibility. Using chemical inducers may have undesirable effects on cells, such as cytotoxicity, impermeability, and disruption of normal metabolism.^[^ ^41^ ^]^ Therefore, the fusion of optogenetics and BEs has a significant application value. Recognizing that the principle of split proteins may also be applicable to the ABE base editing system will provide a solution to reduce the off‐target effects resulting from adenine deaminases.

**Figure 1:**
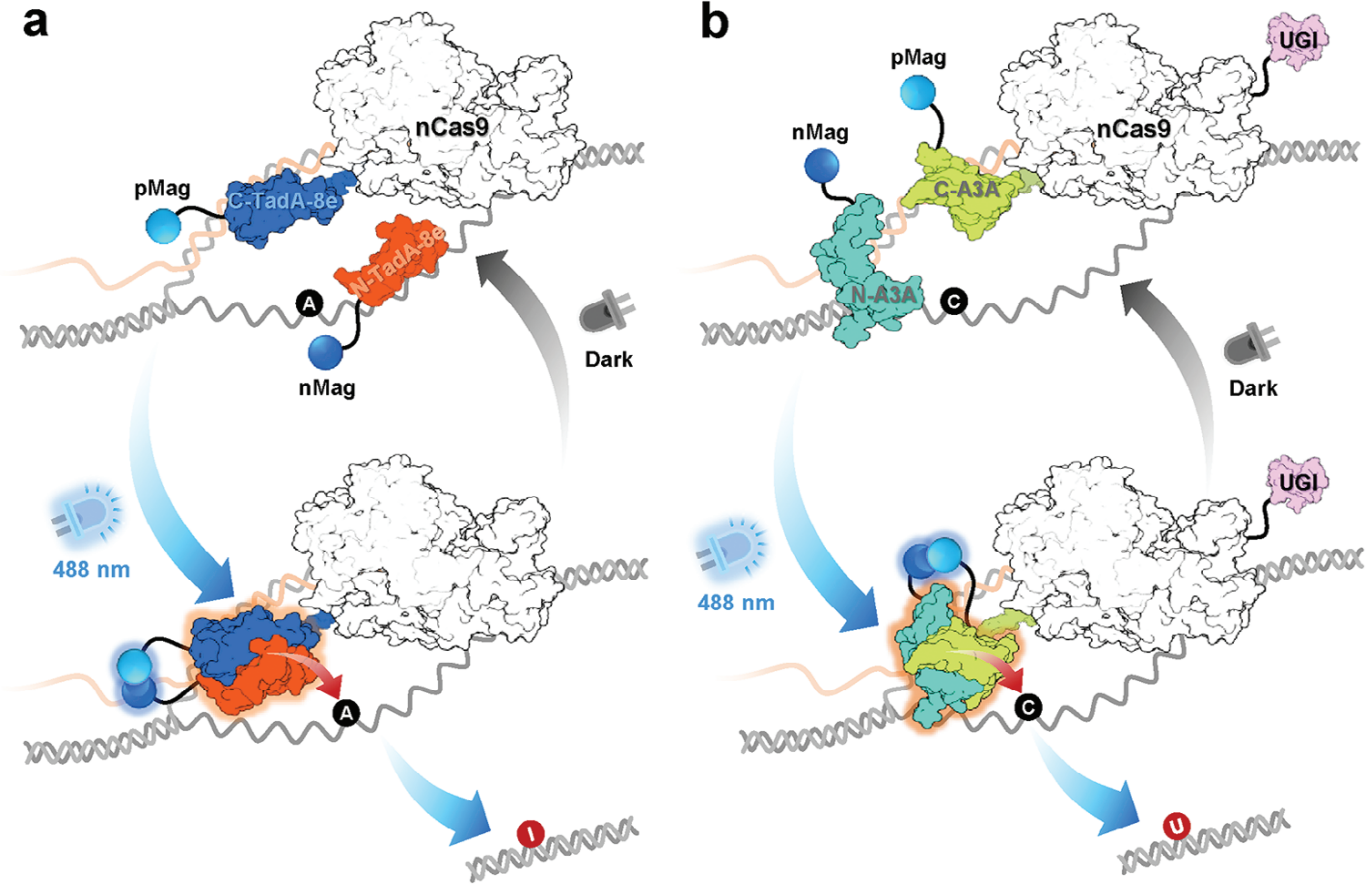
Schematic of BLABE and BLCBE. a) Split DNA deaminase TadA‐8e was linked to two ingredients of Magnets (pMag and nMag) and nCas9, with the N‐terminal TadA‐8e (blue) fused to nMag (orange), and the C‐terminal was tagged with pMag and nCas9, respectively. Under the blue light (488 nm) adaptor stage, the Magnets dimerize, resulting in functional TadA‐8e that converted the target adenine (A) to inosine (I). b) For BLCBE, similar to BLABE, N‐terminal APOBEC3A (cyan blue) was fused to nMag. The C‐terminal APOBEC3A (green) combined with nCas9 and UGI, with pMag attached to C‐terminal APOBEC3A. Blue light (488 nm) permitted the dimerization of N‐ and C‐terminal APOBEC3A to form a functional protein that changed target cytosine (C) to uracil (U). In another instance, the active protein was dissociated and inactivated under the dark stage.

Based on the structural information of TadA‐8e and A3A,^[^ ^42^, ^43^ ^]^ we divided the DNA deaminases into two parts, the N‐terminal and C‐terminal, which were connected with two Magnets,^[^ ^44^ ^]^ respectively, to construct the blue‐light‐activated adenine base editor (BLABE) (**Figure** **1**a) and the blue‐light‐activated cytosine base editor (BLCBE) (Figure 1b). As a potential genome editing tool, it realizes blue light regulation of ABE and CBE for precise gene editing.

## 2 Results

### 2.1 Construction of BLABE and BLCBE Systems in *Escherichia coli*

**Figure 2:**
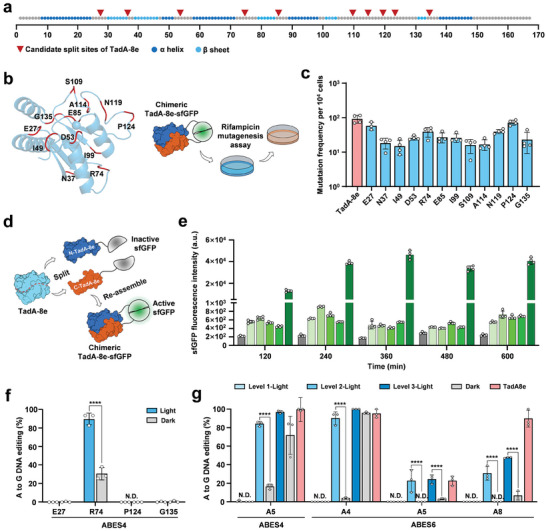
Split deaminases strategy for optically regulated base editing of BLABE. a) Schematic of potential split sites on the primary TadA‐8e structure. Twelve candidate sites for splitting are located in the unstructured loops, indicated with the red triangle, and positioned between two amino acids. The secondary structure of TadA‐8e is highlighted with different colors (α‐helix, dark blue; β‐sheet, light blue). b) Cartoon representation of TaA‐8e protein monomer and the assay of screening test for rifampicin resistance. Left, potential sfGFP insertion sites for TadA‐8e were located after the red‐labeled amino acid. Right, intact TadA‐8e and chimeric TadA‐8e‐sfGFP were transformed into *E. coli* DH10B to evaluate rifampicin resistance mutations, with TadA‐8e as the positive control. c) The frequency of rifampicin‐resistance mutations of intact TadA‐8e and chimeric TadA‐8e variants were measured using the number of Rif^R^ clones on the expression of TadA8e variants in *E. coli* DH10B. d) Strategy for characterizing spontaneous dimerization of split TadA‐8e. The chimeric TadA‐8e‐sfGFP were engineered split within the sfGFP to yield N‐ and C‐terminal chimeric proteins, where the sfGPF was inactive. The sfGFP intensity can be detected until the spontaneous complementation of split TadA‐8e. e) The green fluorescence intensity of split‐sfGFP with various split sites for TadA‐8e. *E. coli* DH10B with split‐proteins variants were incubated up to 600 min at 120‐min intervals and subsequently evaluated for green fluorescence intensity. Intact sfGFP was used as the positive control and *E. coli* DH10B without plasmid (CK^−^) as negative control. f) The efficiency of BLABE on base editing at the target ABES4 site. To investigate the editing efficiency using pMag and nMagHigh1 as preliminary light‐inducing elements. *E. coli* DH10B was transformed with plasmids expressing BLABE featuring various split sites (E27, R74, P124, and G135). Base editing efficiency was evaluated using Sanger sequencing and EditR (N.D. no detected; **** *P* < 0.0001). g) Selection of photoswitches for BLABE system. Candidates contain pMagFast1‐nMagHigh1 (Level 1), pMag‐nMagHigh (Level 2), and pMagHigh1‐nMagHigh1 (Level 3). The efficiency of adenine editing with various Magnets for the BLABE system targeting two different sites (*n* = 3, N.D. no detected; **** *P* < 0.0001). Dots represent individual biological replicates, and bars represent mean ± standard deviation (s.d.) from *n*  =  4 donors (c) or *n*  =  3 donors (e, f, and g).

To construct the BLABE system, we sought to identify a suitable site in TadA‐8e for Magnets insertion based on the crystal structure of the DNA‐bound TadA‐8e complex.^[^ ^42^ ^]^ Structural analysis of the TadA‐8e‐SpCas9 complex revealed the formation of a dimeric assembly with a second copy of TadA‐WT, raising the possibility that TadA‐8e may function in a dimerized form. In a previous study, cytosine DNA deaminases were split via rational manipulation of loop regions.^[^ ^37^ ^]^ TadA‐8e, a TadA DNA deaminase variant, features several loop regions (**Figure** **2**a), suggesting that splitting the TadA‐8e DNA deaminase is feasible in theory. First, we sought to identify sites within the protein that tolerate the insertion of a super‐folder green fluorescent protein (sfGFP) without significantly affecting its activity. Next, we attempted to split sfGFP to evaluate whether the TadA‐8e deaminase can be spontaneously reconstituted from the separate fragments (Figure 2b).^[^ ^45^ ^]^ Finally, eight loops were recognized as insertion locations for sfGFP.

To assess the activity of TadA‐8e deaminase variants, we expressed the fusion proteins in *E.coli* DH10B and subjected these variants to a rifampicin resistance assay.^[^ ^46^ ^]^ The AID/APOBEC family of deaminases can catalyze cytosine deamination to uracil in DNA, resulting in DNA mutations in the *rpoB* gene coding for the β‐subunit of RNA polymerase, giving rise to rifampicin antibiotic resistance.^[^ ^47^ ^]^ TadA‐8e can also induce DNA deamination, indicating the possibility of using rifampin resistance assays to screen for TadA‐8e variants. Using this method, we observed a decrease in the activity of all fusion deaminases (Figure 2c; Figure S1b, Supporting Information), which was unsurprising given that significant modification of the intact protein may affect its stability and function. Owing to the high deamination activity of TadA‐8e, the functionality of the ABE system may not be critically affected by a certain degree of activity loss. Several split sites located distal to the active center are potential sites for splitting. Based on the activity level, spatial relationship, and proximity to the catalytic center, we identified four potential locations in the loops (E27, R74, P124, and G135) suitable for the subsequent construction of the BLABE system (Figure 2c). Notably, E27 and G135 were located at the N‐ and C‐termini of TadA‐8e, respectively, indicating that they may have a minimal impact on the functionality of ABE.

Assuming that the split proteins maintaining a robust capacity for spontaneous association impinges on optogenetic regulatory efficacy and results in high background effects. Next, we split the sfGFP fusion protein into two independent fragments, resulting in a construct pair of N‐TadA‐8e‐sfGFP_1–10_ and sfGFP_11_‐C‐TadA‐8e (Figure S1c, Supporting Information). If split TadA‐8e exhibits robust self‐association, it will elicit a strong fluorescent signal owing to the reassembly of sfGFP. To avoid the split sfGFP reassembled too slow in *E. coli* DH10B, we extended the test time appropriately (0–600 min). The results showed that the candidate split proteins exhibited a negligible fluorescence response (Figure 2e), supporting these split sites (E27, R74, P124, and G135) as potential engineered sites for building the BLABE system.

The use of natural photoreceptors as photoactivatable actuators represents a powerful tool for the optogenetic manipulation of molecular processes in biological systems.^[^ ^48^, ^49^, ^50^, ^51^ ^]^ In previous studies, researchers engineered a series of variants of the Vivid protein, named Magnets, comprising both positive (pMag) and negative (nMag) Magnets, enabling the selection of subsequent blue light‐controlled components.^[^ ^51^ ^]^ Based on the extent of dimerization and dissociation in the mutant variants, we categorized the various combinations into three levels: level 1 (nMagHigh1‐pMagFast1), level 2 (nMagHigh1‐pMag), and level 3 (nMagHigh1‐pMagHigh1). First, the components of level 2, pMag, and nMagHigh1 were fused to the N‐ and C‐termini of split TadA‐8e deaminases, through 10 amino acids (aa) flexible linkers. We named them E27, R74, P124, and G135. To validate this concept, sgRNA ABES4 was employed as a target to evaluate the effect of blue light regulation. In the presence of blue light (10 mW cm^−2^), we observed that blue‐light‐induced target DNA editing of R74 exhibited the highest inducible activity (≈90%) and low background activity (≈30%) under dark conditions (Figure 2f; Figure S1d, Supporting Information). We also conducted functional verification of other split sites with high activity in the rifampin mutagenesis assay, including N37, D53, S109, and N119; however, no editing activity of the target was observed. In conclusion, R74 exhibited exceptional inducible activity and was selected for further optimization.

We tested the effects of various Magnet combinations and constructed a range of BLABE systems to evaluate the efficacy of the base editing. *E. coli* DH10B cells transformed with the above BLABE editors were treated with 10 mW cm^−2^ blue light for 6 h. To assess the effects of blue light in different BLABE systems, ABES4 and ABES6 targets for each condition were quantified by Sanger sequencing. These results indicate a positive correlation between the level of aggregation of the Magnets and the efficiency of target editing. However, the background editing has also increased. At level 1‐BLABE, no targeted editing was observed, whereas at level 3‐BLABE, the efficiency of blue light‐induced editing was close to that of intact ABE, but with higher background editing. For level 2‐BLABE, the target adenine within ABES4 and ABES6 showed the highest editing efficiency (90%) and the lowest editing (3%) at the target base in the presence and absence of blue light (Figure 2g).

Several studies have engineered split cytosine deaminases for rapamycin‐induced regulation of CBE.^[^ ^37^, ^38^ ^]^ Based on previous studies,^[^ ^38^ ^]^ we chose four potential split sites (N42, D85, T118, and G147) that were directly connected to a series of optical Magnets to test the effects of base editing (Figure S2a,b, Supporting Information). Therefore, we constructed a series of blue light‐responsive CBE editing systems with three levels of regulatory elements for four potential split sites. The linker length was standardized to ten amino acids. Consistent with previous reports,^[^ ^38^ ^]^ among these, N42 exhibited the highest editing efficiency (≈100%) across all three base targets within the window of CBES5, but its activity remained under dark conditions (Figure S2c, Supporting Information). Only D85 exhibited a predictable inducible editing activity. For D85, the level 2 inducible blue light Magnets proved to be appropriate, which is consistent with the findings of BLABE (Figure S2d, Supporting Information). T118 and G147 showed comparatively low editing efficiencies within the target region. In addition, these sites also had a high background signal (Figure S2e,f, Supporting Information). Finally, the linker lengths of BLABE and BLCBE were optimized. The results showed that 10 amino acids were the optimal strategy for the systems. Interestingly, the base editing activity of BLABE disappeared when the linker lengths were 5 and 15 amino acids (Figure S3a, Supporting Information); this phenomenon was not observed in BLCBE (Figure S3b, Supporting Information).

### 2.2 Characterization of the BLABE and BLCBE in *E. coli*

To further examine the characteristics of BLABE and BLCBE in response to blue light, we detected the light‐induced duration‐dependent editing efficiency. BLABE and BLCBE were treated with different light intensities, ranging from 2.5–10 mW cm^−2^ (Figure S4a,b, Supporting Information). Surprisingly, the editing efficiencies of BLABE and BLCBE were similar in response to blue light were similar. The base editing efficiency plateau at just 2.5 mW cm^−2^, suggesting that BLABE and BLCBE were sensitive to blue light. These results indicate that BLABE and BLCBE can rapidly complete gene editing in prokaryotic cells.

**Figure 3:**
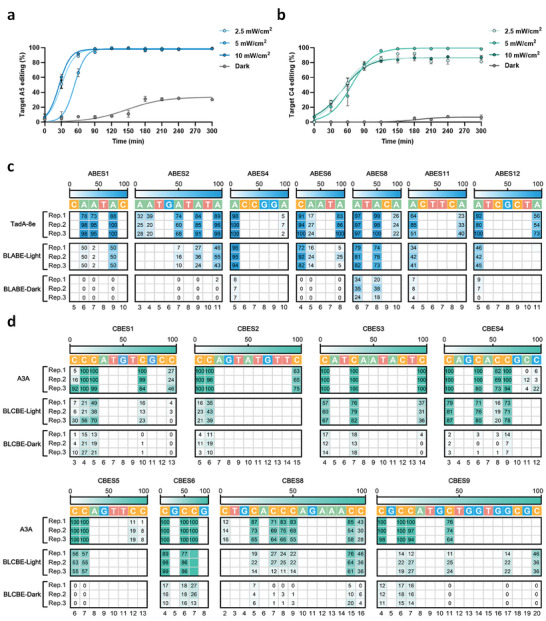
Characterization of BLABE and BLCBE for base editing in E. coli. a) DNA editing efficiency of BLABE under various blue light intensities. The E. coli DH10B with BLABE system targeting ABES4 was treated with varying intensities (dark, 2.5; 5; and 10 mW cm^−2^) of light and dark for 300 min. b) The E. coli DH10B with BLCBE system targeting CBES3 was treated with varying intensities (dark; 2.5; 5; and 10 mW cm^−2^) of blue light and dark for 300 min, then the DNA editing efficiency was analyzed using Sanger sequencing. c) Heat maps show the on‐target adenine editing frequencies of ABE, BLABE‐light, and BLABE‐dark with different sgRNAs targeting genome. d) Heat maps show the on‐target cytosine editing frequencies of CBE, BLCBE‐light, and BLCBE‐dark with different sgRNAs targeting the genome. The editing windows represent the base involved in DNA editing, not the entirety of the protospacer. The numbering at the bottom represents the position of the respective base in the protospacer sequence. All data are shown as individual data points and means ± s.d. for n = 3 independent biological replicates.

We observed that BLABE and BLCBE displayed a notable increase in their background effects post after completion of target base editing under dark conditions (**Figure** **3**a,b). This phenomenon resembles off‐target editing. However, the background base editing effect of BLABE was stronger than that of BLCBE, which stabilized at a plateau. Although more vigorous light intensity‐inducing blue‐light‐activated base editors (BLBEs) can reach saturation gene editing more rapidly (Figure 3a), it may cause phototoxicity and affect editing efficiency (Figure 3b).

Next, we evaluated the editing performance of the light‐controlled base editors in prokaryotic cells to test the capacity of BLABE and BLCBE across a broad range of genomic sites with different characteristics. We randomly selected multiple sgRNA target sites in the genome of *E. coli* DH10B. The results showed that ABE exhibited average editing efficiency of 60.8% for all the investigated loci (Figure 3c). BLABE demonstrated an editing efficiency of 27.2% under blue light‐on conditions, then had a lower background of just only 3.6% editing efficiency under the dark (Figure S4c,e, Supporting Information). The editing efficiency of BLABE was ≈44.7% relative to the intact ABE across all sites. Across these sites, the total average editing efficiency of all sites in the intact CBE was 68.9%. For the BLCBE in the presence of light, on‐target editing efficiency was 33.3% (Figure 3d). In contrast, BLCBE exhibited a significantly higher editing efficiency of 6.1% under dark conditions (Figure S4d,f, Supporting Information). In summary, BLABE and BLCBE exhibited 7.5‐fold and 5.4‐fold differential alterations in light‐induced gene‐editing efficiency prompted by the light, respectively. Notably, split DNA deaminases slightly shifted the position and width of the editing window relative to intact DNA deaminases.

### 2.3 Temporal Application of the BLABE and BLCBE Systems in *E. coli*

The noninvasive modulation of BEs shows significant promise for various applications. Compared with chemical molecules, optical regulation offers precise spatiotemporal control.^[^ ^41^ ^]^ Optogenetic control of BEs may rationally regulate the on‐off‐target editing duration and ratio, whereas manipulating the timing of target genome editing can significantly reduce off‐target effects.^[^ ^50^ ^]^

**Figure 4:**
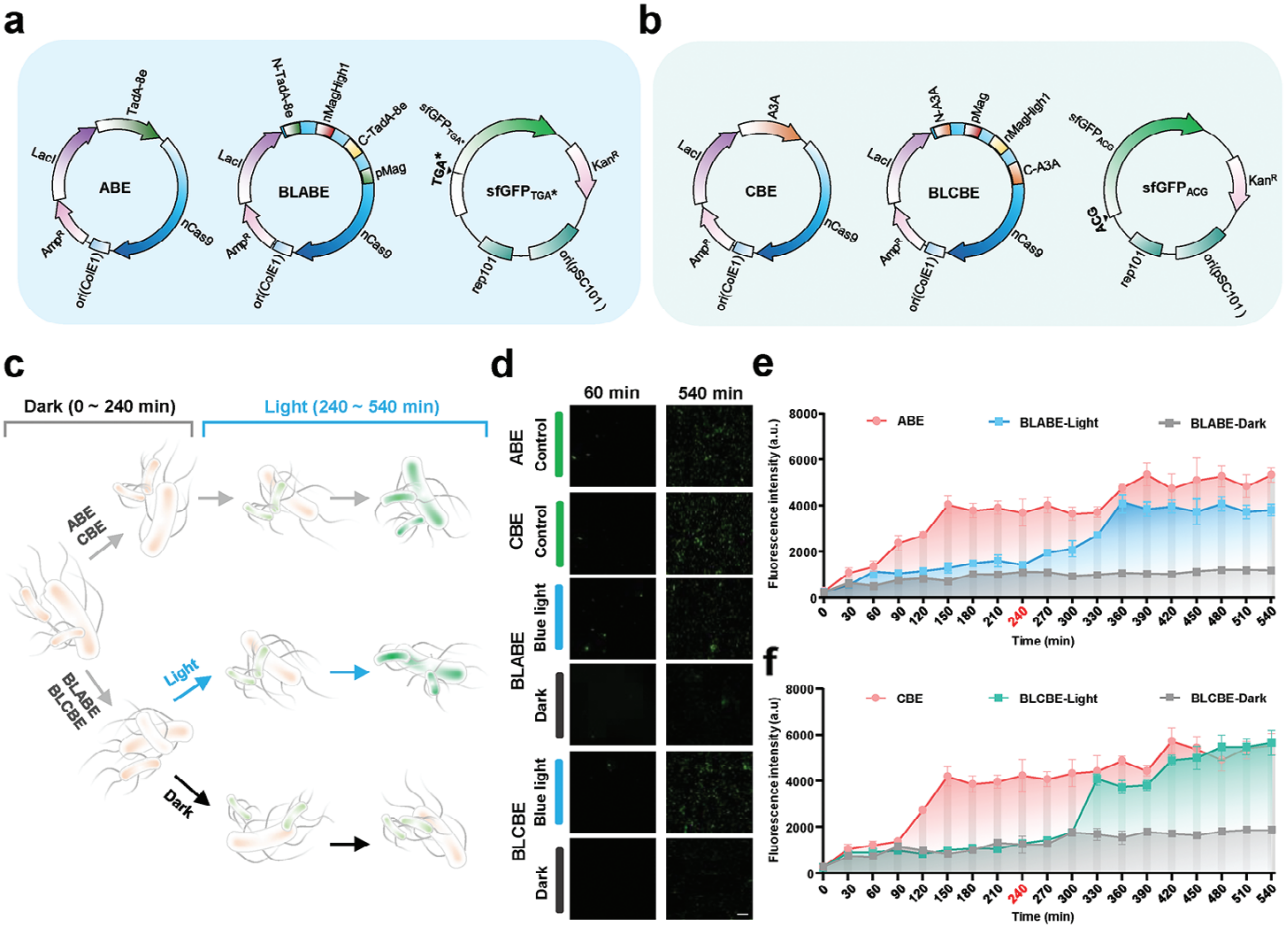
BLBEs enable temporal control of base editing in *E. coli*. a) Schematic illustrating the construction of ABE, BLABE, and target sfGFP_TGA*_ plasmids. b) Schematic illustrating the construction of CBE, BLCBE, and target sfGFP_ACG_ plasmids. c) Temporal control of gene editing of inactivated sfGFP variants by the BLBEs systems. The process was divided into the dark (0–240 min) and light (240–540 min) stages. ABE was in normal light. For BLBEs systems, *E. coli* DH10B with inactive sfGFP was incubated up to 240 min, after which the function of BLBEs was regulated in the presence or absence of light until 540 min. d) Fluorescence image showing sfGFP expression level after cultured for 60 min and treatment with blue light from 240 to 540 min. Scar bar, 20 µm. e,f) Quantification of fluorescence intensity of sfGFP_TGA*_ (e) and sfGFP_ACG_ (f) by MicroplateReader with intact BEs or BLBEs with the presence or absence of blue light changed from 0 to 540 min. The time point of blue light activation (240 min) is marked in red. The mean and s.d. were obtained from three independent biological replicates.

To further explore the feasibility of the temporal control of BLBEs systems, we used the low‐copy inactive sfGFP as a monitor. First, we constructed two inactive mutants of sfGFP, one used for the BLABE assay, which terminated translation prematurely, resulting in an inactive protein named sfGFP_TGA*_ (**Figure** **4**a). The other mutant was used for BLCBE, which involved erasing the start codon so that the protein could not initiate translation, and was named sfGFP_ACG_ (Figure 4b). For sfGFP_TGA*_, BLABE modified the artificially introduced stop codon TAG/TGA in sfGFP_TGA*_ by converting A to G, which restored sfGFP activity. To circumvent the issue that the introduce of mutation in α‐helix or β‐sheet may cause structural damage to sfGFP, the position E142 in the loop region was selected to introduce the stop codon TGA, replaced with W142(TGG) after gene editing. For sfGFP_ACG_, the ATG start codon was replaced with ACG, which can be recovered using CBE. Intact ABE and CBE were co‐transformed into *E. coli* DH10B, with the corresponding inactivated sfGFP mutants as the positive controls. The BLBEs operated rapidly in prokaryotic cells at 30 min intervals and delineated two distinct phases, dark (0–240 min) and light (240–540 min), for spatiotemporal induction characterization (Figure 4c).

We performed GFP imaging assays to evaluate the fluorescence recovery of the sfGFP 1 h after the induction and expression of sfGFP. sfGFP variants did not exhibit detectable fluorescence within 60 min. After the induction of base editing, the green fluorescence was restored (Figure 4d; Figure S5a,b, Supporting Information). The green fluorescence intensity of the BLBEs and sfGFP mutant co‐transformed strains remained low after 540 min in the absence of blue light. We turned on at 240 min to activate the sfGFP variants. Intact ABE and CBE significantly restored the function of the sfGFP variant within 240 min, whereas BLABE and BLCBE remained inactive until blue light activation. We turned on the light at 240 min and observed the rapid accumulation of sfGFP‐variant fluorescence intensity (Figure 4e,f). The fluorescence intensity of BLCBE‐light was similar to that of CBE after 450 min, indicating that BLCBE transformed almost all sfGFP_ACG_ into sfGFP_ATG_ at this time point. Under dark conditions, the green fluorescence intensity attributed to BLABE and BLCBE accumulated slightly over time. This phenomenon is likely attributable to the background editing effects. Interestingly, the background effect observed for BLCBE was greater than that for BLABE, which may be due to the lack of a start codon (AUG) for translation termination.^[^ ^52^ ^]^ Our results show that BLBEs have temporal regulatory specificity, indicating that editing can be effectively regulated noninvasively.

### 2.4 Assessment of DNA Off‐target Effects of BLABE and BLCBE in *E. coli*

BE is a valuable tool for the treatment of diseases and has other applications. However, the utilization of base editors has been hindered by shortcomings, including off‐target DNA editing and other bystander effects.^[^ ^19^, ^21^ ^]^ These two categories can be attributed to the causes of the base editor's off‐target effects: sgRNA‐dependent and sgRNA‐independent off‐targets. The sgRNA‐dependent off‐target effect is caused by mismatching between sgRNA and similar sequence sites, whereas the sgRNA‐independent off‐target effect results from random deamination caused by intact DNA deaminase effector elements.^[^ ^22^, ^23^ ^]^ Therefore, it is necessary to reduce both potential off‐target effects to enhance security of base editors.

**Figure 5:**
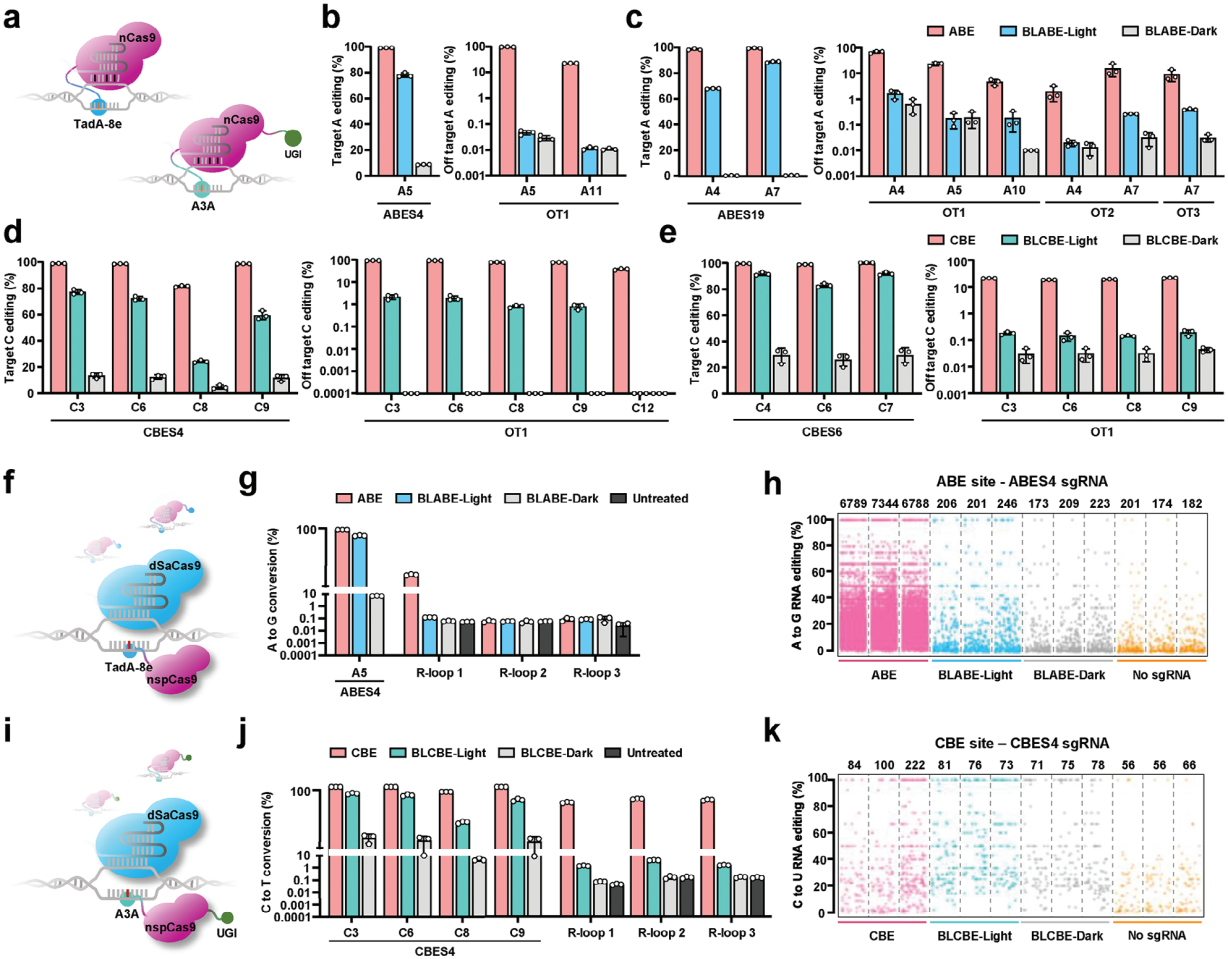
Off‐target editing of base editor systems on the genome and transcriptome. a) Schematic showing sgRNA‐dependent off‐target effects for ABE (left) and CBE (right). b) Left shows quantified on‐target genomic editing for ABE, BLABE, and sgRNA targeting ABES4. Right shows quantified sgRNA‐dependent off‐target genomic editing for BLABE on the OT1. c) Left shows quantified on‐target genomic editing for ABE, BLABE, and sgRNA targeting ABES19. Right shows quantified sgRNA‐dependent off‐target genomic editing for BLABE on the OT1, off‐target 2 (OT2), and off‐target 3 (OT3). d) Left shows quantified on‐target genomic editing for CBE, BLCBE, and sgRNA targeting CBES4. Right shows quantified sgRNA‐dependent off‐target genomic editing for BLABE on the OT1. e) Left shows quantified on‐target genomic editing for CBE, BLCBE, and sgRNA targeting CBES6. Right shows quantified sgRNA‐dependent off‐target genomic editing for BLABE on the OT1. f) Schematic diagram of orthogonal R‐loop assay for evaluating sgRNA‐independent off‐target of TadA‐8e on the genome. g) The sgRNA‐independent off‐target genomic editing was evaluated. The editing efficiency of ABE and BLABE (light and dark) were evaluated using NGS. R‐loop 1, R‐loop 2, and R‐loop 3 show off‐target editing effects at the locus opened by dSaCas9. h) Manhattan scatter plot showing transcriptomic A‐to‐I mutations detected in RNA‐seq experiments from *E. coli* DH10B in which ABE, BLABE‐light, BLABE‐dark with sgRNA targeting ABES4. The *E. coli* DH10B as the negative control expressed no sgRNA. The number of adenines modified is indicated at the top. i) Schematic diagram of orthogonal R‐loop assay for evaluating sgRNA‐independent off‐target of A3A on the genome. j) The sgRNA‐independent off‐target genomic editing was evaluated. The editing efficiency of CBE and BLCBE (light and dark) were evaluated using NGS. R‐loop 1, R‐loop 2, and R‐loop 3 show off‐target editing effects at the locus opened by dSaCas9. k) Manhattan scatter plot showing transcriptomic C‐to‐U mutations detected in RNA‐seq experiments from *E. coli* DH10B in which CBE, BLCBE‐light, BLCBE‐dark with sgRNA targeting CBES3. The *E. coli* DH10B as the negative control expressed no sgRNA. The number of cytosines modified is indicated at the top. All data are shown as individual data points and means ± s.d. for *n* = 3 independent biological replicates.

To verify that the sgRNA‐dependent off‐target effects can be effectively reduced by controlling the deaminase activity and editing time window, we used next‐generation sequencing (NGS) technology to evaluate the off‐target effects caused by the mismatch of sgRNA (**Figure** **5**a). To investigate the potential off‐target effects of BLABE, we selected two targets, ABES4 and ABS19, and their predicted off‐target sites for analysis. We predicted one off‐target site for ABES4 and three off‐target sites for ABES19 and then sequenced the amplicons for these sites. The off‐target editing was detected at all sites for the intact ABE editor using amplicon sequencing, and the base editing efficiency was close to 100%. BLABE exhibited appropriate on‐target editing (Figure 5b,c). Interestingly, for the off‐target positions, the intact ABE off‐target efficiency approached 100%, whereas the off‐target editing of BLABE was significantly low. In addition, the off‐target effects were lower under dark than blue light conditions (Figure S6a, Supporting Information). Similar to BLABE, the BLCBE system also exhibited low off‐target effects. First, we selected two targets, CBES4 and CBES6, with wide editing windows for their off‐target sites to evaluate their off‐target effects. The results showed that the BLCBE worked normally at the on‐target (Figure 5d,e).

We observed that the intact CBE for off‐target 1 (OT1) of CBES4 exhibited high off‐target editing efficacy for all nucleotide bases within the editing window. Conversely, the BLCBE demonstrated only 1% off‐target editing and no off‐target effects were observed in the dark (Figure 5d). Interestingly, when the off‐target efficiency of the intact CBE editor at the CBES6‐OT1 site was reduced by 10‐fold, the BLCBE was also diminished by the same proportion, approximately 0.1% (Figure 5e; Figure S6b, Supporting Information).

Using whole‐genome sequencing to evaluate sgRNA‐independent off‐target DNA is time‐consuming and expensive.^[^ ^53^, ^54^, ^55^ ^]^ Instead, a more economical and timesaving detection method for assessing these effects has been reported. The orthogonal R‐loop assay has been proven to be effective for assessing sgRNA‐independent off‐target activity in eukaryotic cells.^[^ ^56^ ^]^ In this assay, the orthogonal CRISPR system dSaCas9 was used to create single‐stranded DNA (ssDNA) regions in human cells that acted as targets for the sgRNA‐independent deamination of base editors, which can be detected by amplicon sequencing. We then used an orthogonal R‐loop assay to investigate the off‐target activity of BLBEs at the DNA level. We used the dead SaCas9 (dSaCas9) within *lacZ* to generate single‐stranded DNA (ssDNA) regions to examine BLABE and BLCBE sgRNA‐independent off‐target activities (Figure 5f,i). Three sites were selected: R‐loops 1, 2, and 3. *E. coli* DH10B was co‐transformed with two plasmids encoding 1) an ABE or CBE base editor with an ABES4 or CBES4‐targeting sgRNA, and 2) the dSaCas9 with a SaCas9 sgRNA targeting the genomic *lacZ* gene. In this assay, the on‐target editing of ABES4 and CBES4 for BLABE and BLCBE was unaffected by dSaCas9. Under light conditions, the target editing efficiency of BLBEs was similar to that of the intact ABE and CBE base editors (Figure 5g,j). The results showed that ABE exhibited a relatively weak off‐target in these R‐loop regions: only the R‐loop 1 displayed a 15% off‐target efficiency, and the remaining R‐loops showed no significant off‐target activity. Furthermore, BLABE had no obvious off‐target effects on these R‐loops (Figure 5g; Figure S7a, Supporting Information), under blue light or dark, the off‐target editing of BLABE was near the background (mean 0.14% and 0.07%, respectively). In contrast, CBE showed a higher sgRNA‐independent off‐target effect, with an off‐target editing efficiency of a mean of 59% across all R‐loop regions. In the presence of blue light, off‐target editing efficiency of R‐loops for BLCBE was 2.5%. In the dark, off‐target editing was ≈0.1% (Figure 5j; Figure S7b, Supporting Information). Thus, the low off‐target effects of BLABE and BLCBE at the DNA level indicated that optogenetic control of base editors can significantly mitigate the off‐target activity.

To investigate the transcriptome‐wide off‐target RNA editing profiles of BLABE and BLCBE, RNA‐sequencing (RNA‐seq) was performed. The results showed that negative control exhibited background levels in the transcriptome with few RNA A‐to‐G and C‐to‐U edits. Although ABE did not exhibit higher off‐target activity in the orthogonal R‐loop assay, it induced relatively high levels of A‐to‐G editing in the transcriptome, which may be related to its origin in tRNA deaminases. BLABE treatment did not significantly increase the number of A‐to‐G transcripts in either the presence or absence of blue light. However, there was a slight increase in the efficiency compared to the untreated group (Figure 5h; Figure S8a,b, Supporting Information). In contrast to the ABE results, intact CBE did not exhibit significant off‐target activity in the transcriptome. For intact CBE, the number of C‐to‐U deamination of RNA was 1.78‐ and 2.4‐fold higher than that of BLCBE and the negative control, respectively (Figure 5k). Compared to the negative control, the frequency of C‐to‐U mutations was only 1.3‐fold higher, which was lower than the positive control (Figure S8c, Supporting Information). Based on these results, BLABE and BLCBE demonstrated a reduced off‐target activity in the transcriptome compared to intact base editors.

### 2.5 Characterization of the Gene Editing Performance of BLABE and BLCBE Systems in Mammalian Cells

Efficient base editing in response to blue light by the BLABE and BLCBE systems in prokaryotic cells motivated us to think further regarding whether the system also applies to eukaryotic cells. To avoid the potential high activity of BLBEs in the absence of blue light, we first investigated the performance of base editing of BLCBE in HEK293T cells using a P2A self‐cleaving peptide, internal ribosome entry sequence (IRES), and dual‐plasmid expression strategies. For the dual‐plasmid construct, the N‐ and C‐termini of split A3A were expressed separately from the two independent plasmids. Unexpectedly, we observed low BLCBE activity in the presence of light under the IRES design. The construction with P2A‐light (36%) and dual‐plasmid‐light (32%) strategies demonstrated a high level of base editing activity compared to CBE (44%), where P2A‐dark construct (13%) had higher background activity relative to dual‐plasmid‐dark expression system (9%) in the dark (Figure S9a, Supporting Information). For the dual‐plasmid strategy, we further investigated the ratio of the N‐ and C‐termini of the split A3A. The 1:1 ratio was determined to exhibit superior light‐activated base editing efficiency (45%) and low‐level background activity (7%) in the dark (Figure S9b, Supporting Information). Thus, the dual‐plasmid construct permitted 6.4‐fold inducible control over base editing by separately expressing the split fragments of A3A. The BLABE was then constructed using the same approach.

**Figure 6:**
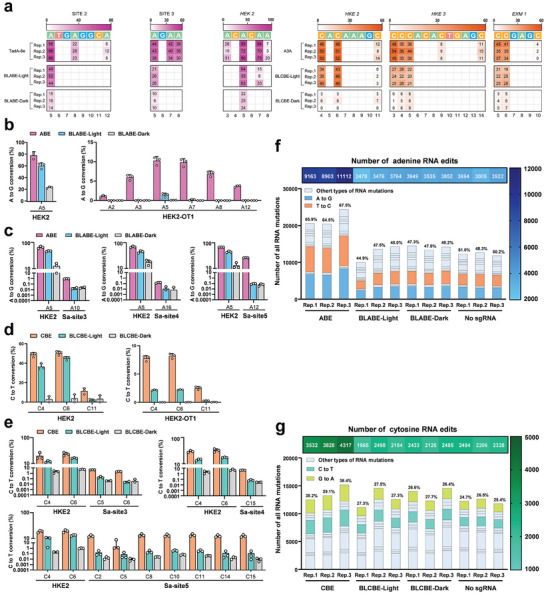
Off‐target editing of BLABE and BLCBE on the genome and transcriptome in HEK293T. a) Left, heat maps show on‐target adenine editing frequencies of ABE, BLABE‐light, and BLABE‐dark with different sgRNAs targeting HEK293T genome. Right, heat maps show on‐target cytosine editing frequencies of CBE, BLCBE‐light, and BLCBE‐dark with different sgRNAs targeting HEK293T genome. The editing windows represent the base involved in DNA editing, not the entirety of the protospacer. The numbering at the bottom represents the position of the respective base in the protospacer sequence. b) Schematic showing sgRNA‐dependent off‐target effects for ABE and BLABE. Left shows that on‐target genomic editing was quantified for ABE and BLABE with sgRNA targeting HEK2. Right shows that sgRNA‐dependent off‐target genomic editing was quantified for ABE and BLABE on the off‐target 1 (HEK2‐OT1). c) Schematic diagram of orthogonal R‐loop assay for evaluating sgRNA‐independent off‐target of ABE and BLABE on the genome. The sgRNA‐independent off‐target genomic editing was evaluated. The editing efficiency of ABE and BLABE (light and dark) were evaluated using NGS. Sa‐site3, Sa‐site4, and Sa‐site5 show off‐target editing effects at the locus opened by dSaCas9. d) Schematics show sgRNA‐dependent off‐target effects for CBE and BLCBE. Left shows quantified on‐target genomic editing for CBE and BLCBE with sgRNA targeting HEK2. Right shows quantified sgRNA‐dependent off‐target genomic editing for ABE and BLABE on the off‐target 1 (HEK2‐OT1). e) Schematic diagram of orthogonal R‐loop assay for evaluating sgRNA‐independent off‐target of CBE and BLCBE on the genome. The sgRNA‐independent off‐target genomic editing was evaluated. The editing efficiency of CBE and BLCBE (light and dark) were evaluated using NGS. Sa‐site3, Sa‐site4, and Sa‐site5 show off‐target editing effects at the locus opened by dSaCas9. f) The combination of column stacking plot and heat map for the off‐target efficiency of ABE and BLABE on the transcriptome. Cloum stacking plot shows the proportion of various types of RNA mutation in the total transcriptomic SNPs mutation. The number indicated the percentage of A to G and T to C mutation frequencies across all mutation types of the transcriptome (left) and the hot map indicated the number of A to G and T to C mutations at the transcriptome (right). g) The combination of column stacking plot and heat map for the off‐target efficiency of CBE and BLCBE on the transcriptome. Cloum stacking plot shows the proportion of various types of RNA mutation in the total transcriptomic SNPs mutation. The number indicated the percentage of C to T and G to A mutation frequencies across all mutation types of the transcriptome (left) and the hot map indicated the number of C to T and G to A mutations at the transcriptome (right). All data are shown as individual data points and means ± s.d. for *n* = 3 independent biological replicates.

To evaluate the base editing activities of the BLABE and BLCBE systems in HEK293T cells, five sgRNAs (SITE2, SITE3, HEK2, HEK3, and EXM1) targeting widely used human genomic sites were selected for BLBEs.^[^ ^1^, ^2^ ^]^ Across these sites (SITE2, SITE3, and HEK2), the intact ABE average on‐target editing efficiency was 75%. BLABE exhibited 38% base editing efficiency across the sites in the presence of light and background on‐target base editing of 5% in the dark. In the presence of blue light, base editing efficiency was increased by 7.6‐fold, reaching 51% of the ABE (**Figure** **6**a). For BLCBE, we observed that the intact CBE exhibited 40% base editing efficiency across these sites (HEK2, HEK3, and EXM1). In the presence of blue light, BLABE can realize 28% on‐target editing efficiency and exhibited 4% low background on‐target efficiency in the dark. Similar to BLABE, the base editing activity was increased by 7‐fold, reaching 70% of the intact CBE under the blue light (Figure 6a). As expectedly, the activity variations of BLABE and BLCBE were similar to those observed in prokaryotic cells.

### 2.6 Quantification of sgRNA‐Dependent and Independent Off‐target Editing of BLABE and BLCBE in Mammalian Cells

Based on the effective on‐target base editing performance of BLBEs in HEK293T cells, we evaluated the off‐target effects of BLABE and BLCBE. For sgRNA‐dependent off‐target base editing, the binding sites of the base editors were similar to those of the on‐target sgRNA. To evaluate off‐target effects, we selected the sgRNAs targeting HEK2 genomic sites and well‐established off‐target sites for HEK2‐targeting sgRNAs. We observed that BLABE and BLCBE maintained 60% and 40% on‐target editing efficiency at HEK2, respectively. The results showed that intact ABE and CBE had off‐target editing activities at the HEK2‐OT1, with mean efficiencies ranging of 1.1–10.2% and 2.5–8.1%, respectively (Figure 6b,d). Notably, the off‐target editing efficiencies of HEK2‐OT1 of BLABE and BLCBE were reduced to 0.007–1.4% and 0.02–2.2%, respectively (Figure 6b,d; Figure S10a,b, Supporting Information).

To determine sgRNA‐independent off‐target efficiency of BLBEs in HEK293T cells, we examined the off‐target activity at the DNA level using an orthogonal R‐loop assay and employed an RNA‐seq method to detect RNA off‐target effects. In the orthogonal R‐loop assay, the mean base editing efficiencies of BLABE and BLCBE at the target site HEK2 were 47.3% and 15.1%, respectively, approximating intact ABE (mean 67%) and CBE (mean 27%). Besides, the on‐target editing efficiencies were only 14% and 3.4% without light (Figure 6c,e). To assess off‐target sgRNA‐independent efficiency at the DNA level, three R‐loop off‐target sites were selected for BLABE and BLCBE. Intact ABE (0.15–26.1%) and CBE (4.1–18.8%) exhibited off‐target efficiencies across all R‐loop sites. In the presence of blue light, BLABE and BLCBE exhibited low mean off‐target efficiencies of 0.036% and 0.73%, respectively, slightly higher than those observed in the dark (Figure 6c,e; Figure S11a–c and S12a–c, Supporting Information).

Similar to prokaryotic cells, sgRNA‐independent transcriptome‐wide deamination of RNA has also been observed in eukaryotic cells. To evaluate off‐target activity in the transcriptome, we performed RNA‐seq on HEK293T cells that underwent different treatments using various types of base editor systems. Interestingly, BLABE exhibited a high transcriptome off‐target effect, whereas BLCBE did not show an off‐target activity equivalent to that of BLABE, which aligned with the tendency of RNA off‐target activity observed in *E. coli* DH10B.

Intact ABE showed significant off‐target activity at the RNA‐level, whereas the BLABE system effectively reduced off‐target activity, regardless of the presence or absence of light, and had a low off‐target activity similar to that of the controls (Figure 6f; Figure S13a, Supporting Information). Furthermore, our results revealed that ABE maintained a higher level of off‐target activity when evaluating the amount of off‐target RNA and mutation percentage. This resulted in an off‐target efficiency that was of 1.74‐ and 1.81‐fold higher than that of the no‐sgRNA and BLABE controls, respectively (Figure S13c, Supporting Information).

Although intact CBE also caused some degree of off‐target effects at the RNA level, our engineered BLABE system still mitigated the off‐target effects to natural levels under both light and dark conditions (Figure 6g; Figure S13b, Supporting Information). A comparative analysis of the RNA off‐target efficiency revealed that the off‐target efficiency of intact CBE at the transcriptome level was 1.70‐fold higher than that of BLCBE. Notably, the constructed BLCBE system displayed an off‐target efficiency similar to that of the blank control (Figure S13d, Supporting Information). Subsequently, calling the locations where the single nucleotide polymorphisms (SNPs) occurred at the transcriptome level to the chromosomes revealed that intact ABE and CBE exhibited considerable off‐target editing across various chromosomal loci. In contrast, the engineered BLABE and BLCBE systems were similar to the control, with no treatment for off‐target activity (Figure S14 and S15, Supporting Information). Collectively, the DNA deaminases of ABE and CBE resulted in sgRNA‐independent off‐target transcriptome editing. In addition, ABE exhibited a comparatively elevated level of off‐target effects compared to CBE, whereas the engineered BLABE and BLCBE could significantly reduce such off‐target effects.

## 3 Discussion

Base editing is a breakthrough technology that mediates the targeting of single‐nucleotide conversations without requiring double‐stranded DNA breaks.^[^ ^1^, ^2^ ^]^ To improve the efficiency of targeted base editing, several studies have developed highly active deaminases for base editor systems. It has been reported that the adenine deaminase mutant TadA‐8e exhibits the highest activity so far.^[^ ^11^ ^]^ Unfortunately, the increased DNA deaminase activity in BEs results in elevated CRISPR off‐target effects, which pose a significant barrier to precise gene editing. Engineering DNA deaminases in BEs through amino acid mutations to obtain a series of variants with low off‐target activity is a promising priority.^[^ ^57^, ^58^, ^59^ ^]^ However, this process can be time‐consuming because of the potential unwanted effects on the native function of proteins.

Spatiotemporal control utilizing the split‐Cas9 strategy has demonstrated effectiveness in enhancing the precision of Cas9 targeting and mitigating off‐target effects.^[^ ^33^, ^34^, ^35^, ^36^ ^]^ These innovative systems have enabled researchers to finely regulate Cas9‐mediated genome modifications by restricting Cas9 activity or its life cycle, thereby reducing off‐target effects by minimizing the exposure of the cell genome to nucleases. However, base editors based on the split‐Cas9 strategy remain susceptible to sgRNA‐independent off‐targeting,^[^ ^60^ ^]^ which has been proven to be detrimental to both the genome and transcriptome because deaminases remain intact and unrestricted within the cells.^[^ ^22^, ^23^ ^]^

Therefore, the split‐deaminases strategy appears to be a more efficient approach for lowering sgRNA‐independent off‐targeting. Berríos et al. and Long et al. has successfully developed split‐CBE based on the chemically induced dimerization proteins.^[^ ^37^, ^38^ ^]^ In the preparation of this manuscript, Zeng et al. reported a split‐ABE based on the aforementioned small‐molecule‐induced dimerization system.^[^ ^61^ ^]^ However, the inducer rapamycin, exhibited potential cytotoxicity and cannot be rapidly removed once used, posing unpredictable risks to cell viability.^[^ ^62^ ^]^ In contrast, BLBEs utilize blue light as the inducer, which is non‐invasive and easy to remove, thereby improving the security and resolution of induction. Meanwhile, BLBEs showed similar on‐target efficiency and low off‐target effects compared to the chemically induced split base editors. However, BLBEs showed a slight increase in background editing, suggesting that more subtle adjustments should be made to pMag and nMag to achieve lower background binding while maintaining efficient photo‐induced binding.

Furthermore, BLBEs have versatile architectures that can be used in various applications. Several high‐fidelity deaminases, such as A3A(N57G),^[^ ^19^ ^]^ A3A(Y130F),^[^ ^17^ ^]^ and TadA‐8e(N108Q/L145T),^[^ ^63^ ^]^ exhibited capacity for enhanced precision. Notably, the key mutation responsible for improving the fidelity was far away from the split site in BLBEs. Therefore, we hypothesized that integrating these fidelity‐improving mutations into BLBEs may further reduce off‐target effects, potentially approaching background levels in cells. In addition, TadA‐8e has been engineered to achieve more types of base editing, including C to T,^[^ ^64^, ^65^, ^66^ ^]^ C‐to‐G,^[^ ^65^ ^]^ A&C‐to‐G&T,^[^ ^64^, ^66^ ^]^ and A‐to‐Y,^[^ ^67^ ^]^ which are theoretically fully compatible with BLABE in theory. In addition, the SpCas9 protein utilized in BLBEs can be replaced by other small Cas proteins, such as IscB^[^ ^68^ ^]^ or Cas12f,^[^ ^69^ ^]^ both of which are compact enough for packaging into a single adeno‐associated virus (AAV) for clinical therapeutic applications.

Additionally, we showed that the rifampin assay, which was used to identify suitable split sites, was feasible for cytosine deaminases as well as for adenine deaminases. Thus, this strategy can be applied to other deaminases, such as APOBEC3B,^[^ ^59^ ^]^ TadA orthologs,^[^ ^70^ ^]^ and even the non‐deaminase N‐methylpurine DNA glycosylase,^[^ ^71^ ^]^ which has been shown to achieve G‐to‐Y editing.

In summary, BLBEs are promising tools for optogenetics with high blue light‐dependent editing efficiency and low off‐target effects, and should be further engineered and applied for a diverse range of purposes.

## 4 Experimental Section

### Plasmid Cloning

The DNA fragments of APOBEC3A, TadA‐8e, Vivid and sfGFP were synthesized by GenScript. Unless stated otherwise, cloning was performed using the Hieff Clone One Step Cloning Kit (YEASEN). PCR was performed using Phanta Max Super‐Fidelity DNA polymerase (Vazyme). Plasmid site‐directed mutagenesis to construct variants of Vivid were performed using Mut Express II Fast Mutagenesis Kit V2 (Vazyme).

For bacterial studies with TadA‐8e, the TadA‐8e fragment was amplified and cloned into pET28a(+) under the control of the IPTG‐inducible T7‐*lacO* promoter. Then, the sfGFP fragment was amplified and cloned inside TadA‐8e fragment in different insertion sites.

For construction of bacterial all‐in‐one base editor plasmid, A3A or TadA‐8e was amplified and replaced the APOBEC1 in pEcBE3^[^ ^72^ ^]^ or TadA‐TadA* in pEcABE,^[^ ^73^ ^]^ obtaining pEcA3A and pEcA8e under the control of IPTG‐inducible *lac* operator with ampicillin resistance gene. Various levels of pMag and nMag were amplified and inserted into pEcA3A and pEcA8e to construct pEcBLCBE and pEcBLABE. J23119‐BsaI‐BsaI‐sgRNA scaffold cassette was pre‐embedded to clone and express the sgRNA sequence. Briefly, Protospacer oligos were phosphorylated by PNK (Thermo Fisher Scientific) and cloned into BsaI‐digested base editor plasmids by T4 DNA Ligase (Thermo Fisher Scientific). In addition, all‐in‐one p15A‐based pdSaCas9 with kanamycin resistance gene was constructed by combining the SaCas9 sgRNA expression cassette under the control of J23119 promoter with dSaCas9‐UGI cassette under the native constitutive promoter of SpCas9.

For construction of mammalian base editor plasmids, A3A or TadA‐8e was amplified and replaced APOBEC1 fragment in pCMV‐BE3^[^ ^1^ ^]^ or TadA‐TadA* of pCMV‐ABE7.10,^[^ ^2^ ^]^ obtaining pCMV‐A3A and pCMV‐A8e. Fragment of pMag was amplified and inserted into pCMV‐A3A or pCMV‐A8e to create pCMV‐pMag‐A3AC or pCMV‐pMag‐A8eC. Fragment of nMagHigh1, N‐terminal of A3A, and N‐terminal of TadA‐8e were amplified and cloned into the backbone of pCMV‐BE3 to create pCMV‐A3AN and pCMV‐A8eN. The nMagHigh1‐IRES‐pMag or nMagHigh1‐P2A‐pMag was inserted into A3A in pCMV‐A3A to create pCMV‐IRES‐BLCBE or pCMV‐P2A‐BLCBE.

For sfGFP activation assay, psfGFP was constructed via combing the J23119‐RBS‐sfGFP with the backbone containing ori (pSC101) and kanamycin resistance gene. The initiation condon ATG of sfGFP was replaced with ACG to construct the psfGFP_ACG_. The condon of E142 was mutated to TGA, constructing the psfGFP_TGA*_.

### Blue Light Irradiation Setup

Blue light irradiation was performed using six LED lamps (465 nm peak). The irradiation intensity was measured by the light meter (A5813, SMART SENSOR) and adjusted by changing the distance of the lamps and plates. The average light intensity was 2.5–10 mW cm^−2^ for *E. coli* DH10B and 2 mW cm^−2^ for HEK293T. Plates were wrapped in tinfoil for the negative control under the dark.

### Culture Condition and Transfection

For base editing in bacteria, *E. coli* DH10B and *E. coli* B21(DE3) were cultured in LB broth. On‐target genomic base editing was performed by the transformation of *E. coli* DH10B competent cells with plasmids encoding base editors. For orthogonal R‐loop assays, an all‐in‐one SpCas9‐containing plasmid was co‐transformed with pdSaCas9. After overnight culture, a single colony was selected and seeded into a 96‐well plate (Corning) containing 200 µL LB broth with 1 mM IPTG (Sigma‐Aldrich) for each well. The blue light was turned on 30 min later.

For base editing in mammalian cells, HEK293T (ATCC CRL‐3216) cells were kept in Dulbecco's modified Eagle medium (DMEM, Gibco) supplemented with 10% (vol/vol) fetal bovine serum (FBS, Gibco) in 5% CO_2_ incubator (Crystal) at 37 °C.

HEK293T cells were seeded into 48‐well plates (Corning) at a density of 55000 cells per well and were transfected 18–24 h after plating. Transfection was performed using Lipofectamine 3000 (Invitrogen) according to the manufacturer's instructions. For on‐target genomic editing, 500 ng SpCas9‐containing plasmids and 250 ng guide RNA plasmids were co‐transfected with 250 ng pCMV‐A3AN or pCMV‐A8eN for the experimental group and 250 ng pUC57 for the positive control group. For orthogonal R‐loop assays, 300 ng SpCas9‐containing plasmids, 200 ng SpCas9 guide RNA plasmids, 300 ng dSaCas9 plasmids, 200 ng SaCas9 guide RNA plasmids were co‐transfected with 150 ng pCMV‐A3AN or pCMV‐A8eN for the experimental group and 150 ng pUC57 for the positive control group. The blue light was turned on 24 h later, and cells were cultured for another 48 h.

### Design of Split DNA Deaminases Constructs

The structure of the SpCas9‐ABE8e complex (PDB 6vpc) was downloaded from the Protein Data Bank and analyzed in PyMol (The PyMOL Molecular Graphics System, Version 2.4 Schrödinger, LLC). After removing the SpCas9 backbone, TS, NTS, and guide RNA sequences, the TadA‐8e monomer was kept. For exploring all potentially active split sites, every site in the middle of the loop regions of TadA‐8e was considered.

### Bacterial Rifampicin Assay

The pET28a‐based TadA‐8e expression plasmid and the sfGFP‐inserted plasmid were transformed into chemically competent *E. coli* B21(DE3). After overnight cultured in LB agar plates with kanamycin (50 µg mL^−1^) at 37 °C, a single colony was selected and seeded into 10 mL LB medium, and 1 mM IPTG was added when OD_600_ reached 0.4. Overnight cultures were diluted to 10^−5^‐fold in 1 mL LB medium 12 h later. Then, the LB medium was transferred to LB agar plates containing rifampicin (100 µg mL^−1^) and kanamycin (50 µg mL^−1^). After air drying, plates were cultured at 37 °C overnight. The number of survival colonies represented the activity of deamination.

### sfGFP Activation Assay

All‐in‐one base editors with or without Magnets targeting inactive sfGFP gene fragments were co‐transformed with sfGFP_ACG_ or sfGFP_TGA*_ into *E. coli* DH10B. *E. coli* with active sfGFP was set as the positive control. A single colony was selected and seeded in a 96‐well plate harboring 200 µL LB medium with 1 mM IPTG. All the plates were wrapped in tinfoil in the “dark‐off” phase (0–240 min). At the same time, all plates were irradiated by blue light except the BLCBE‐Dark and BLABE‐Dark groups in the “light‐on” phase (240–540 min). The fluorescent intensity was measured every 30 min by the fluorescence microplate reader (TECAN).

### Extraction of Gnomic DNA

To extract bacterial genomic DNA, overnight induced *E. coli* DH10B in a 96‐well plate was transferred into a 0.2 mL PCR tube. After centrifugation, the precipitate was resuspended in 100 µL TE buffer (10 mM Tris‐HCl, 1 mM EDTA, pH 8.0). Then, tubes were heated at 95 °C for 20 min and cooled at 4 °C. After centrifugation, the supernatant could be used as the DNA template for the amplicon.

To extract HEK293T genomic DNA, cells were washed with PBS and lysed in 100 µL DNA lysis buffer (10 mM Tris‐HCl, pH 8.0, 5 mM EDTA, 0.1% SDS, 30 µg mL^−1^ Proteinase K (YEASEN)) at 37 °C for 1 h. After heating at 80 °C for 20 min, the DNA solution was stored at −80 °C.

### Targeted Amplicon Sequencing

For next‐generation sequencing, PCR amplification was performed using primers synthesized with different barcodes. PCR products were verified on 2% agarose gel and purified using Universal DNA Purification Kit (Tiangen Biotech). Amplicons with various barcodes were mixed together and sequenced on an Illumina MiSeq instrument according to the manufacturer's protocols (GENEWIZ). The quality of raw sequencing reads was evaluated using Fastp, and those with a quality score below 15 were discarded. Adapters were trimmed using PANDAseq, and Fastq multx was used to demultiplex the paired sequences. CRISPResso2^[^ ^74^ ^]^ was used to calculate the editing efficiency.

For sanger sequencing, the target regions were PCR amplified and then analyzed by Beijing Tsingke Biotech Co., Ltd. The results were quantified using EditR.^[^ ^75^ ^]^

### Off‐target Amplicon Sequencing

SgRNA‐dependent off‐target sites were investigated using Cas‐OFFinder.^[^ ^76^ ^]^ SgRNA‐independent off‐target sites were consistent with dSaCas9 targeting sites in the orthogonal R‐loop assays. Next‐generation sequencing was performed as described above. CRISPResso2^[^ ^74^ ^]^ was used to calculate the editing efficiency of off‐target sites.

### Analysis of RNA Off‐target Editing

The *E. coli* DH10B harboring base editors were cultured in a 6‐well plate and induced. Overnight cultures were collected in a 50 mL tube and centrifuged in 6500 r.p.m. for 10 min. After resuspending in TRIzol (Thermo Fisher Scientific), the precipitates were frozen in liquid nitrogen.

HEK293T cells were seeded into the 6‐well plate at an appropriate density. About 20 h later, 3 µg SpCas9‐containing plasmids and 1.5 µg guide RNA plasmids were co‐transfected with 1.5 µg pCMV‐A3AN or pCMV‐A8eN for the experimental group and 1.5 µg pUC57 for positive control group using 6 µL lipofectamine 3000. The negative control group was transfected with 6 µg guide RNA plasmids and kept in thick tinfoil all the time. The blue light was turned on 24 h later, and cells were cultured for another 48 h. Cells were washed in pre‐cooled DPBS and collected in a 15 mL tube. After centrifugation in 1200 r.p.m. for 10 min, cells were resuspended in TRIzol and frozen in liquid nitrogen.

RNA purification, reverse transcription, library construction, and sequencing were performed at Majorbio Bio‐pharm Biotechnology Co., Ltd. Briefly, total RNA was extracted from the cells according to the manufacturer's instructions of TRIzol, and genomic DNA was removed using DNase I (TaKaRa). RNA degradation and contamination were monitored on 1% agarose gels. Then RNA quality was determined by 2100 Bioanalyser (Agilent Technologies) and quantified using the ND‐2000 (NanoDrop Technologies). Only high‐quality RNA sample was used to construct the sequencing library.

The RNA library was sequenced using Illumina NovaSeq 6000 sequencer in 2×150 bp read length. The raw paired‐end reads were trimmed and quality controlled by fastp^[^ ^77^ ^]^ with default parameters. Then clean reads were separately aligned to the reference genome with orientation mode using HISAT2^[^ ^78^ ^]^ software. StringTie assembled the mapped reads of each sample in a reference‐based approach.^[^ ^79^ ^]^ SNP calling analysis was performed by Sentieon Genomics Suite.

### Statistics Analysis

All statistical analysis were performed on at least *n* = 3 biologically independent experiments using an unpaired two‐tailed student's *t*‐test through GraphPad Prism 9 (GraphPad Software). All data were expressed as the mean ± SD. Statistical significance was set at *P* < 0.05.

## Conflict of Interest

The authors declare no conflict of interest.

## Author Contributions

Y.S. and Q.C. contributed equally to this work. Y. S. and Q.C. conceptualized the idea. Y.S. and Q.C. designed and performed the experiments. Y.C. analyzed the next‐generation sequencing data. X.W. conducted subsequent additional experiments. Y.S. and Q.C. wrote the manuscript with contributions from all authors. Y.S. reviewed and revised this manuscript.

## Supporting information

Supporting Information

Supplemental Data 1

Supplemental Data 2

Supplemental Data 3

Supplemental Data 4

## Data Availability

The data that support the findings of this study are available in the supplementary material of this article.
