# Supplementary material for: Design and Engineering of Light‐Induced Base Editors Facilitating Genome Editing with Enhanced Fidelity
Source: Adv Sci (Weinh). 2023 Dec 1;11(5):2305311. doi: 10.1002/advs.202305311 (PMC10837352; doi:10.1002/advs.202305311)
Supplement: Supplementary file 1 — Supporting Information [file ADVS-11-2305311-s002.pdf]

ADVANCED SCIENCE
Open Access

Supporting Information

for *Adv. Sci.*, DOI 10.1002/advs.202305311

Design and Engineering of Light-Induced Base Editors Facilitating Genome Editing with Enhanced Fidelity

*Yangning Sun, Qi Chen, Yanbing Cheng, Xi Wang, Zixin Deng, Fuling Zhou and Yuhui Sun**

ADVANCED SCIENCE
Open Access

Supporting Information

Design and engineering of light-induced base editors facilitating genome editing with enhanced fidelity

*Yangning Sun, Qi Chen, Yanbing Cheng, Xi Wang, Zixin Deng, Fuling Zhou, and Yuhui Sun**

Y. Sun, Q. Chen, Y. Cheng, X. Wang, Z. Deng, F. Zhou, Y. Sun
Department of Hematology
Zhongnan Hospital of Wuhan University
School of Pharmaceutical Sciences
Wuhan University
Wuhan, 430071, China
E-mail: yhsun@whu.edu.cn

Y. Sun, Q. Chen, Y. Cheng, X. Wang, Z. Deng, Y. Sun
Key Laboratory of Combinatorial Biosynthesis and Drug Discovery (Ministry of Education)
Wuhan University
Wuhan, 430071, China

Y. Sun, Q. Chen
These authors contributed equally to this work

Table of Contents

Supporting Figures

Figure S1. The construction and functional verification of blue light-activated adenine base editor.

Figure S2. Split deaminases strategy for the construction of BLCBE

Figure S3. The optimization of linker length of BLABE and BLCBE.

Figure S4. The performance of BLABE and BLCBE for base editing in *E. coli* DH10B.

Figure S5. Fluorescence image showing sfGFP variants expression.

Figure S6. Allele frequencies in the entire amplicon of DNA on-target and sgRNA-dependent off-target editing at diverse loci in *E. coli* DH10B.

Figure S7. Allele frequencies in the amplicon of DNA on-target and sgRNA-independent off-target editing at diverse genomic loci in *E. coli* DH10B.

Figure S8. Total RNA mutation types in the transcriptome.

Figure S9. The optimization of expression strategies and plasmid ratio for HEK293T cells transfection of BLCBE.

Figure S10. Allele frequencies in the amplicon of sgRNA-dependent off-target editing at genomic loci for ABE, BLABE, CBE, and BLCBE systems.

Figure S11. Allele frequencies in the amplicon of sgRNA-independent off-target editing at diverse genomic loci for ABE and BLABE systems.

Figure S12. Allele frequencies in the amplicon of sgRNA-independent off-target editing at diverse genomic loci for CBE and BLCBE systems.

Figure S13. Off-target editing of base editor systems on the transcriptome in HEK293T cells.

Figure S14. RNA off-target editing induced by ABE and BLABE at all chromosome locations.

Figure S15. RNA off-target editing induced by CBE and BLCBE at all chromosome locations.

Supporting Tables

Table S1. Target sgRNA-protospacer sequence for *E. coli* DH10B and HEK293T in this study.

Table S2. Sequences of protospacers and primers for sgRNA-independent and dependent off-target sites for *E. coli* DH10B and HEK293T in this study.

Supporting Sequences

Sequence S1. Amino acid sequences used for *E. coli* DH10B in this study.

Sequence S2. Amino acid sequences used for HEK293T in this study.

a

The number of rifampicin mutant clones

TadA-8e split sites for insertion of sfGFP	TadA-8e	E27	N37	I49	D53	R74	E85	I99	S109	A114	N119	P124	G135
Rep. 1	40	12	2	5	11	11	10	8	1	13	1	18	15
Rep. 2	53	22	3	6	15	15	15	6	1	11	4	32	12
Rep. 3	52	25	3	7	13	13	16	13	3	17	5	24	20
Rep. 4	44	17	5	6	9	20	12	9	11	14	7	21	12

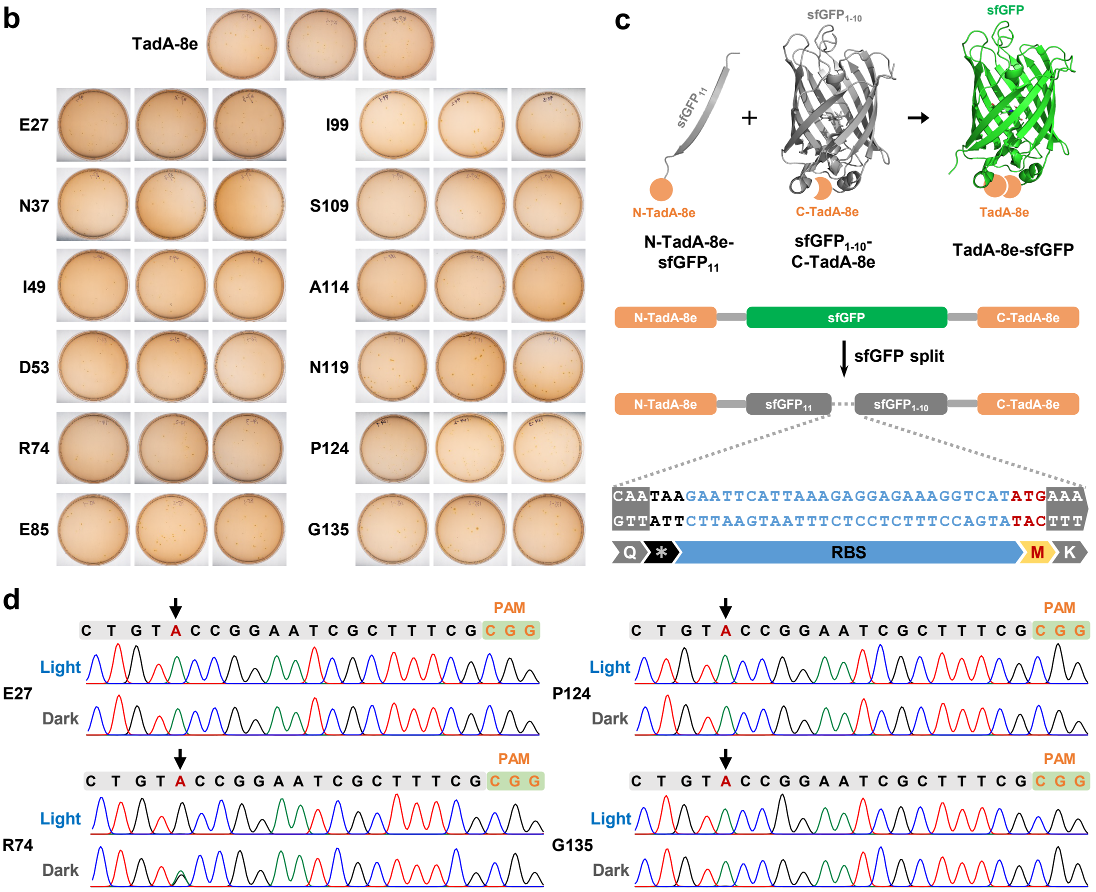

Figure S1. The construction and functional verification of blue light-activated adenine base editor. a) The number of clones with rifampicin resistance for all chimeric sfGFP-TadA-8e proteins. Counts of four independent replicates (*n* = 4) for each protein variant are displayed. b) Photos of clones for *E. coli* DH10B containing rifampicin resistance. Intact TadA-8e servers as the positive control. The photos from three independent experiments. c) Schematic diagram of split sfGFP for analyzing the spontaneous dimerization of split TadA-8e variants. The dimerization of split-TadA-8e (N-TadA-8e and C-TadA-8e) induces the polymerization of sfGFP$_{1\text{-}10}$ and sfGPF$_{11}$ to recover the function of fluorescence. Introduce a DNA sequence within the sfGFP coding gene to split sfGFP, including a stop codon (TAA), ribosome binding site (RBS), and an initiation codon (ATG). d) DNA sequencing chromatograms of different BLABE systems with different split sites in the presence and absence of blue light. The arrow indicates that the red base is the potential editing site.

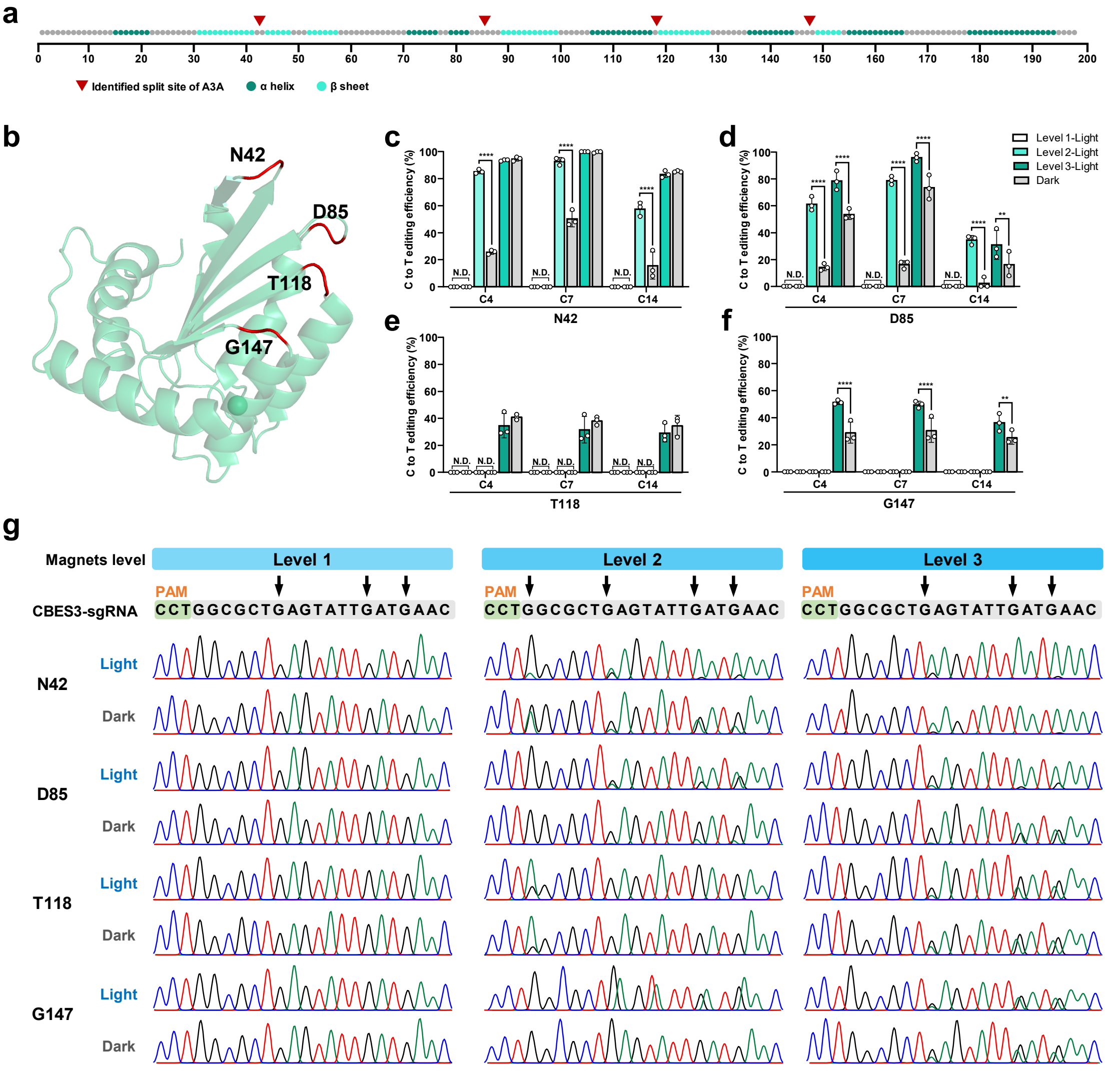

Figure S2. Split deaminase strategy for the construction of BLCBE. a) Schematic of potential split sites on the APOBEC3A (A3A) amino acid sequence. Four candidate sites for splitting are located in the loop area, marked with the red triangle, and split positioned between two amino acids. The secondary structure of A3A is highlighted in different colors (α helix, dark blue; β sheet, light blue). b) Cartoon representation of A3A protein. The potential sfGFP insertion sites for A3A are behind the red-labeled amino acids. c-f) Base editing efficiency of various BLCBE systems, including N42 (c), D85 (d), T118 (e), and G147 (f). Base editing efficiency is calculated by EditR (N.D. no detected; *n* = 3 independent replicates). g) DNA sequencing chromatograms of selection of photoswitches for different BLCBE systems. The Magnets are classified into three levels, and the reverse sgRNA sequences are shown. All possible editing sites are marked with black arrows.

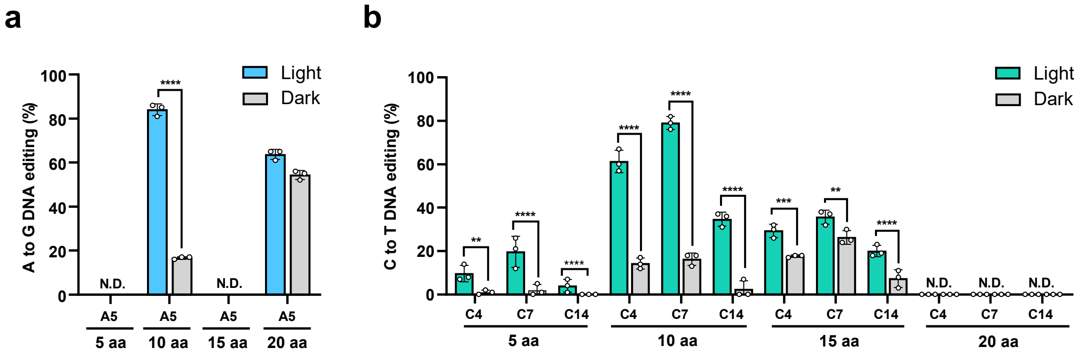

Figure S3. The optimization of linker length of BLABE and BLCBE. a) Target adenine base editing efficiency for various lengths of linker for BLABE. The efficiency of target editing with different linkers of the BLABE system targeting the ABES4 site is shown by the bar chart (*n* = 3, N.D. no detected). b) Target cytosine base editing efficiency for various lengths of linker for BLCBE. The bar chart shows the efficiency of target editing with different linkers of the BLCBE system targeting the CBES3 site (*n* = 3, N.D. no detected).

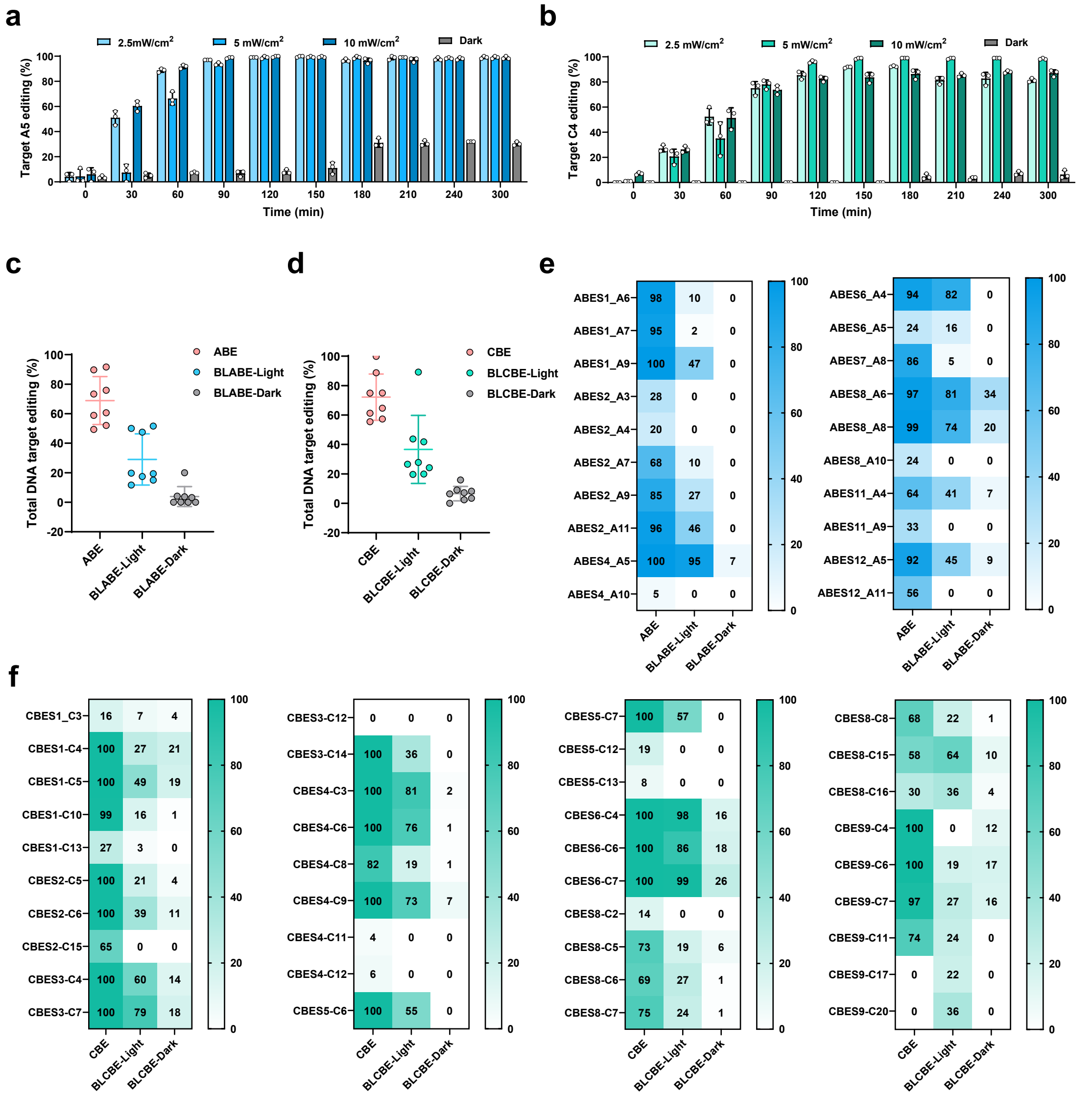

Figure S4. The performance of BLABE and BLCBE for base editing in *E. coli* DH10B. a) The bar plots showing the on-target DNA base editing efficiency of BLABE under various blue light intensities. The target site ABES4 is treated with different light intensities (2.5 mW cm^{-2}; 5 mW cm^{-2}; 10 mW cm^{-2}) for 300 min. b) Bar plots showing on-target DNA base editing efficiency of BLCBE under various blue light intensities. The target site CBES3 is treated with different light intensities (dark; 2.5 mW cm^{-2}; 5 mW cm^{-2}; 10 mW cm^{-2}) for 300 min. The editing efficiency of ABES4 and CBES3 are calculated from three independent replicates (n = 3). c,d) Scatter plots showing the efficiency of all base editing at diverse loci in the genome of BLABE (c) and BLCBE (d) Each data point represents three biological replicates. Data are presented as Mean values. e,f) Heat maps showing the base editing efficiency of BLABE (e) and BLCBE (f) in *E. coli* DH10B at diverse genomic loci. The editing efficiency is marked in the heat map. Intact ABE and CBE serve as the positive control. These data are shown from n = 3 independent biological replicates.

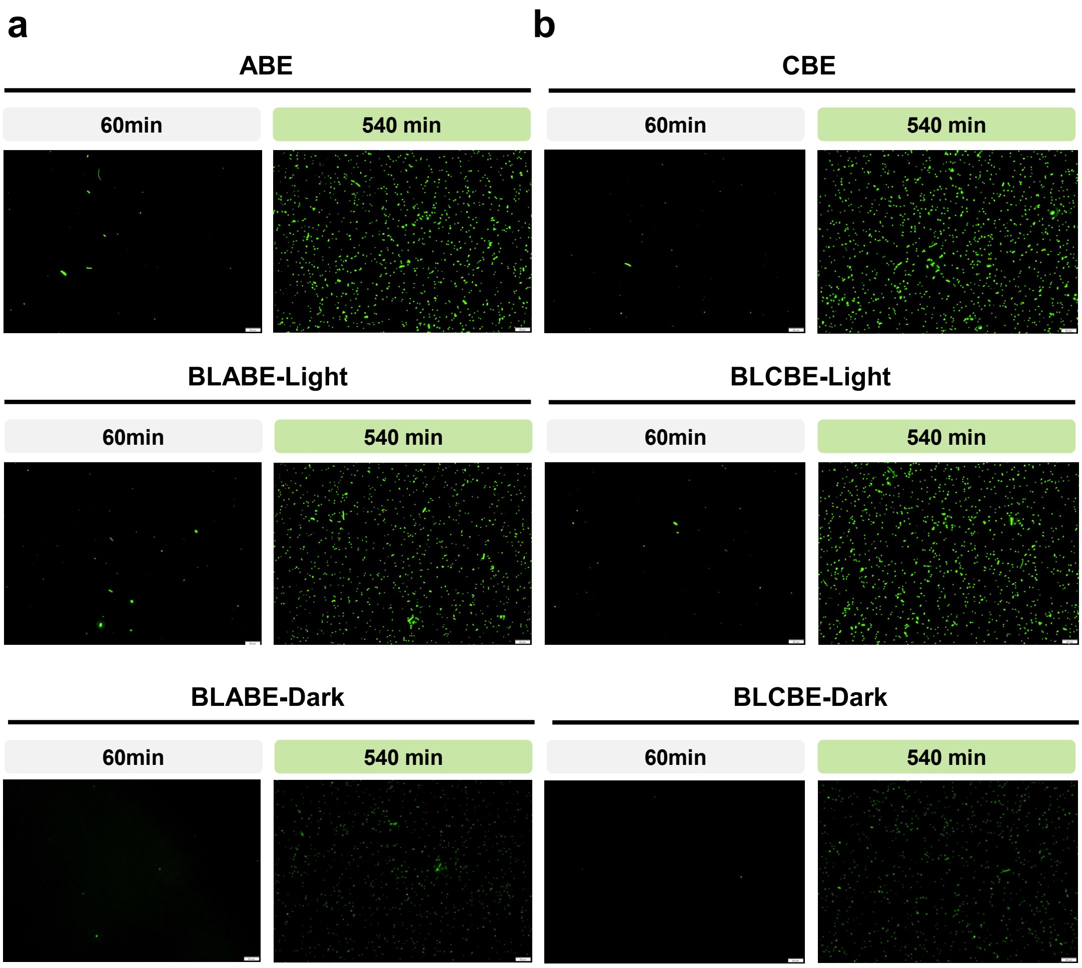

Figure S5. Fluorescence image showing sfGFP variants expression. a, b) The *E. coli* DH10B with plasmids expressing the sfGFP mutants and BLABE (a) or BLCBE (b) are cultured for 540 min and at 240 min treated with blue light. The fluorescence images are obtained at 60 and 540 min under light and dark conditions. Scar bar, 20 μm. Intact ABE and CBE serve as positive controls.

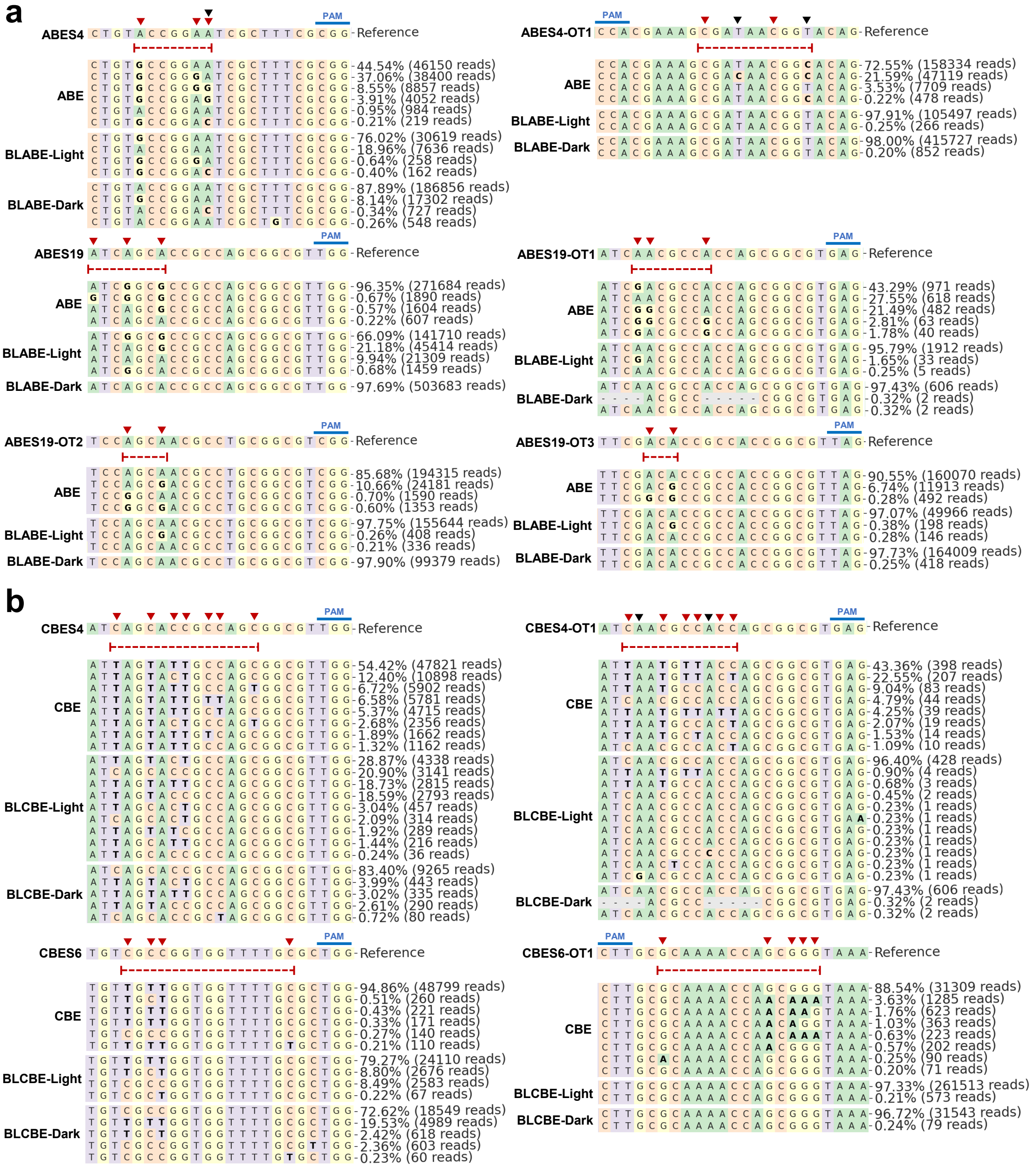

Figure S6. Allele frequencies in the entire amplicon of DNA on-target and sgRNA-dependent off-target editing at diverse loci. a) Allele nucleotide percentages of DNA on-target and off-target base editing of BLABE targeting two sites, ABES4 and ABES19. ABE serves as the positive control, and the target editing efficiency of BLABE is tested in the presence and absence of blue light. b) Allele frequencies of DNA on-target editing within target sites and off-target allele efficiency at diverse genomic loci in the presence and absence of blue light for BLCBE. The possible editing sites within the protospacer sequence are marked by red arrows and the unexpected editing are indicated by black arrows. The base substitutions and deletions are represented with bold letters and short dashes, respectively. The editing window is indicated by red double-ended dotted lines. PAM sequences for SpCas9 (NGG) and SaCas9 (NNGRRT) are marked by short blue lines. The values on the right of the graph represent frequencies and mutation alleles' reads (n = 3).

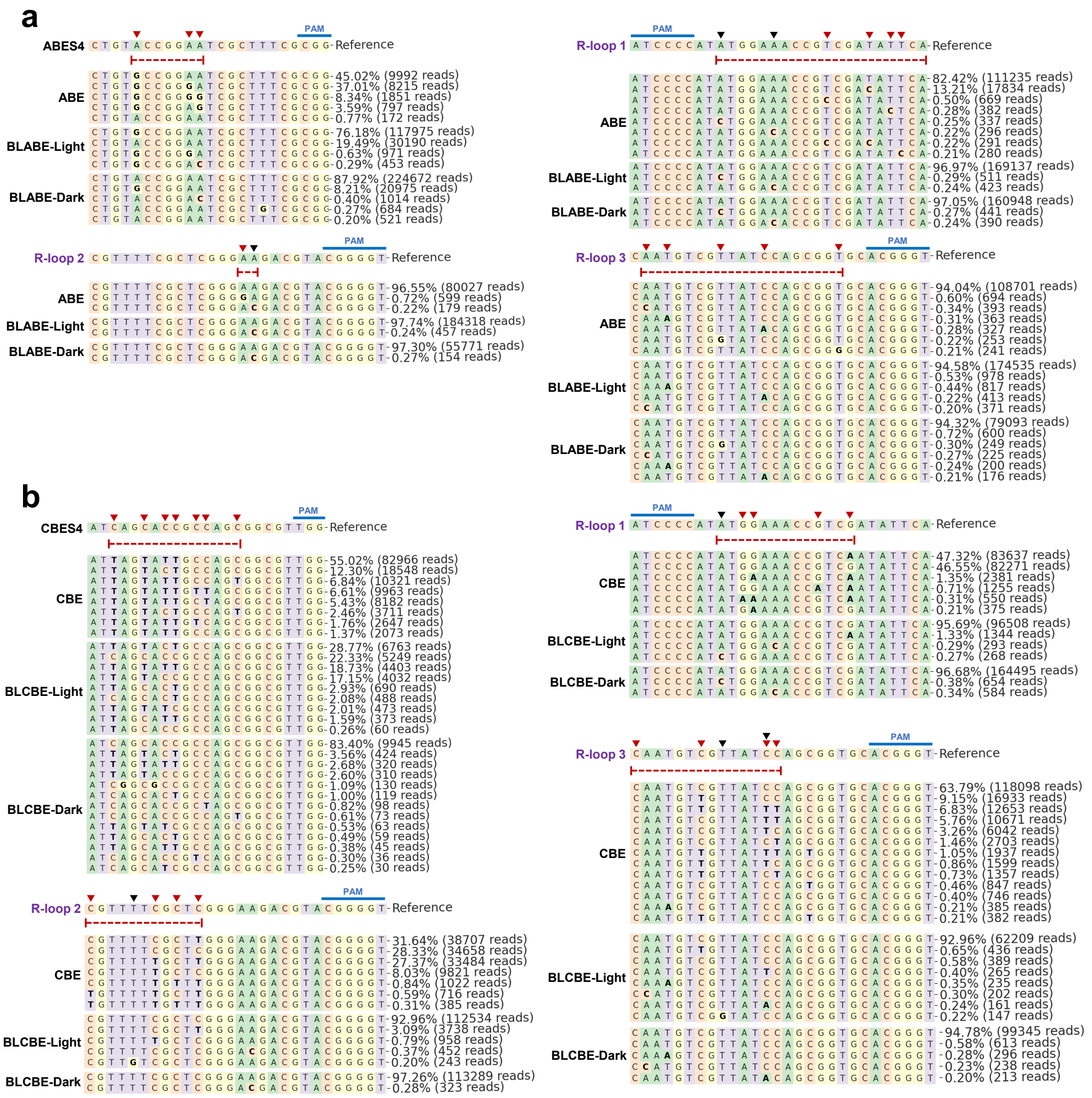

Figure S7. Allele frequencies in the amplicon of DNA on-target and sgRNA-independent off-target editing at diverse genomic loci. a,b) Allele frequencies of on-target editing and sgRNA-independent off-target for BLABE (a) and BLCBE (b) in the presence and absence of blue light. The allele frequencies of off-target within the R-loop region are calculated by amplicon sequencing, and the protospacer sequence is pointed by the purple font. The possible editing sites within the protospacer sequence are marked by red arrows and the unexpected editing are indicated by black arrows. The base substitutions and deletions are represented with bold letters and short dashes, respectively. The editing window is indicated by red double-ended dotted lines. PAM sequences for SpCas9 (NGG) and SaCas9 (NNGRRT) are marked by short blue lines. The values on the right of the graph represent frequencies and mutation alleles' reads (n = 3).

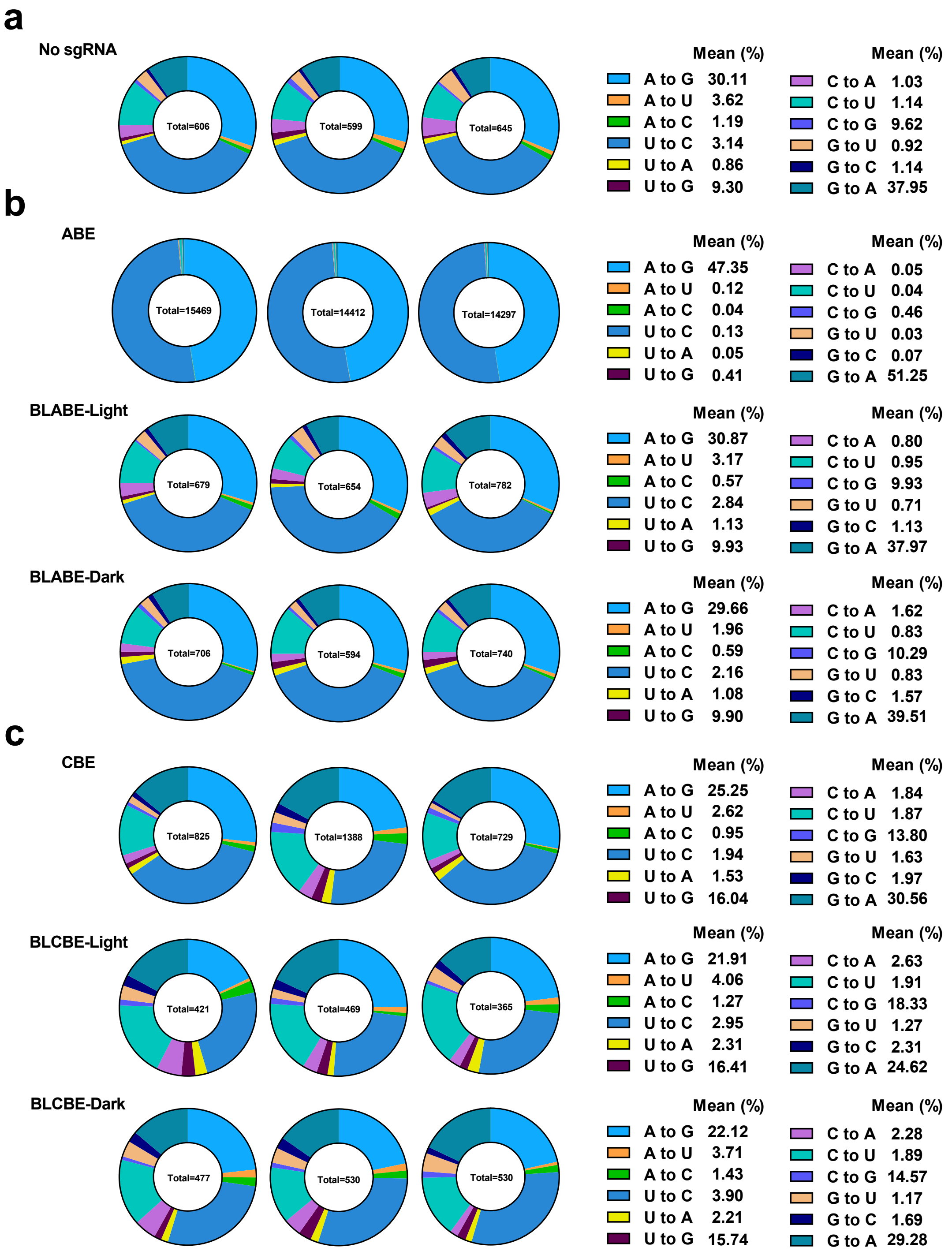

Figure S8. Total RNA mutation types in the transcriptome. a-c) The frequencies of total RNA mutation types in the transcriptome for *E. coli* DH10B (a, negative control), adenine base editor (b), and cytosine base editor (c). The *E. coli* DH10B is not treated as a negative control. Left, the donut chart shows the proportion of various types of RNA mutation in the total transcriptomic single nucleotide polymorphism (SNP) mutation. Right, the mean values of RNA mutation frequencies across all mutation types of the transcriptome. The RNA mutation frequencies of three independent replicates are displayed (n = 3).

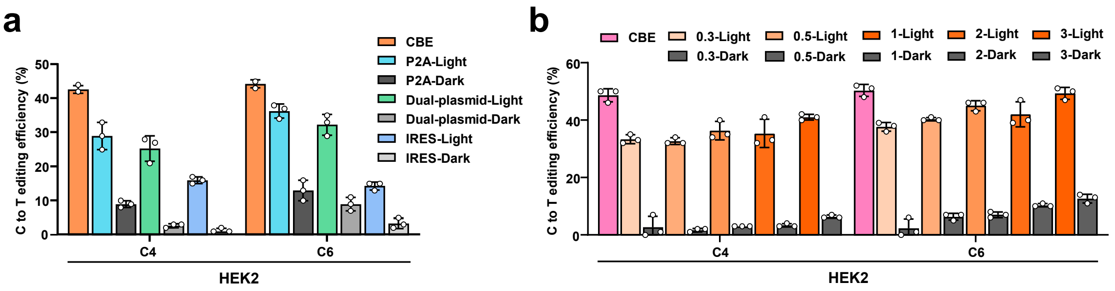

Figure S9. The optimization of expression strategies and plasmid ratio for HEK293T cells transfection of BLCBE. a) Target cytosine base editing efficiency for various strategies of expression for BLCBE, HEK293T cells were transfected using BLCBE with various protein expression strategies, where the BLCBE systems were expressed using P2A, IRES, and cleavage assays. The base editing efficiency of HEK2 is shown by the bar chart (*n* = 3, N.D. no detected). b) Target cytosine base editing efficiency for transfection of HEK293T using dual plasmids system. The numbers of 0.3 ~ 3 in the legend mean the ratios of transfected plasmids (pCMV-A3AN:pCMV-pMag-A3AC). The bar chart shows the efficiency of HEK2 editing efficiency using different ratios of the plasmids for the expression of BLCBE. (*n* = 3, N.D. no detected).

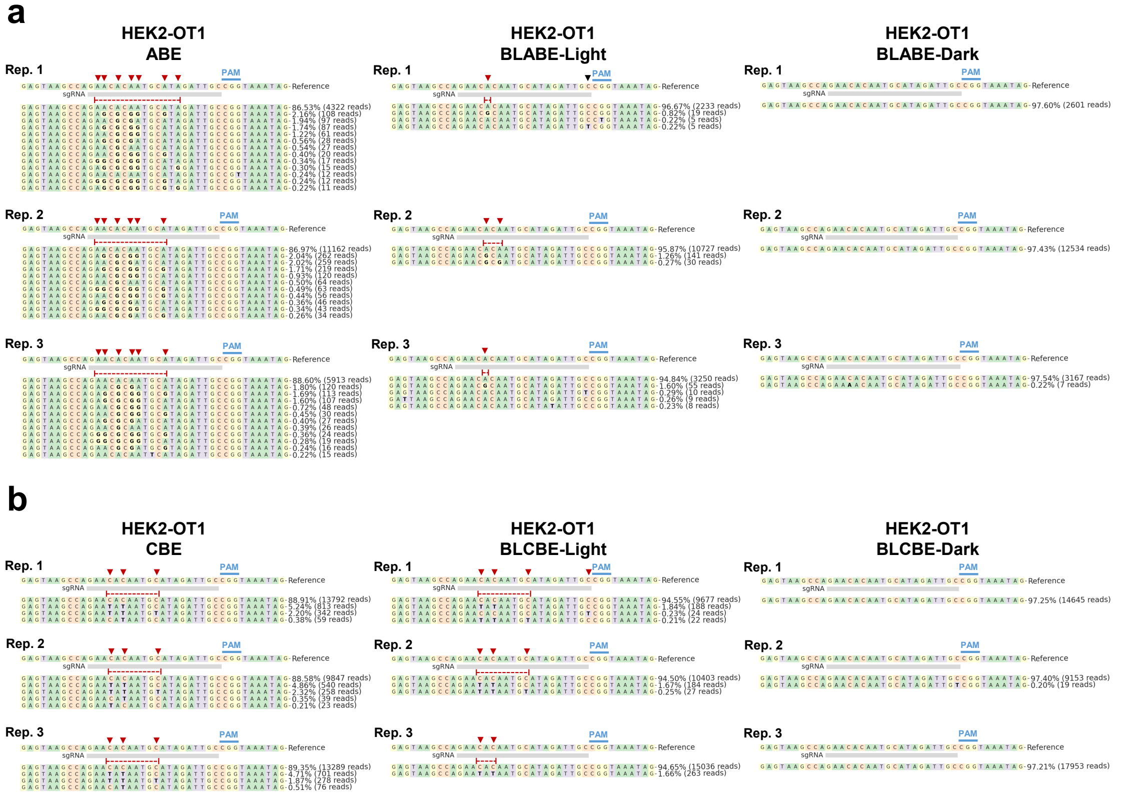

Figure S10. Allele frequencies in the amplicon of sgRNA-dependent off-target editing at genomic loci for ABE, BLABE, CBE, and BLCBE systems. a) Allele frequencies of sgRNA-dependent off-target for ABE and BLABE with sgRNA targeting HEK2. The off-target site was selected by Cas-OFFinder and named HEK2-OT1. The BLABE system was treated under blue light and darkness respectively. b) Allele frequencies of sgRNA-dependent off-target for CBE and BLCBE with sgRNA targeting HEK2. The off-target site was selected by Cas-OFFinder and named HEK2-OT1. The BLCBE system was treated under blue light and darkness respectively. The grey rectangle indicates the sequence of protospacer within sgRNA. The allele frequencies of off-target within the R-loop region are calculated by amplicon sequencing. The values on the right of the graph represent frequencies and mutation alleles' reads. The possible editing sites within the protospacer sequence are marked by red arrows and the unexpected editing are indicated by black arrows. The base substitutions and deletions are represented with bold letters and short dashes, respectively. The editing window is indicated by red double-ended dotted lines. PAM sequences for SpCas9 (NGG) and SaCas9 (NNGRRT) are marked by short blue lines. The values on the right of the graph represent frequencies and mutation alleles' reads (n = 3).

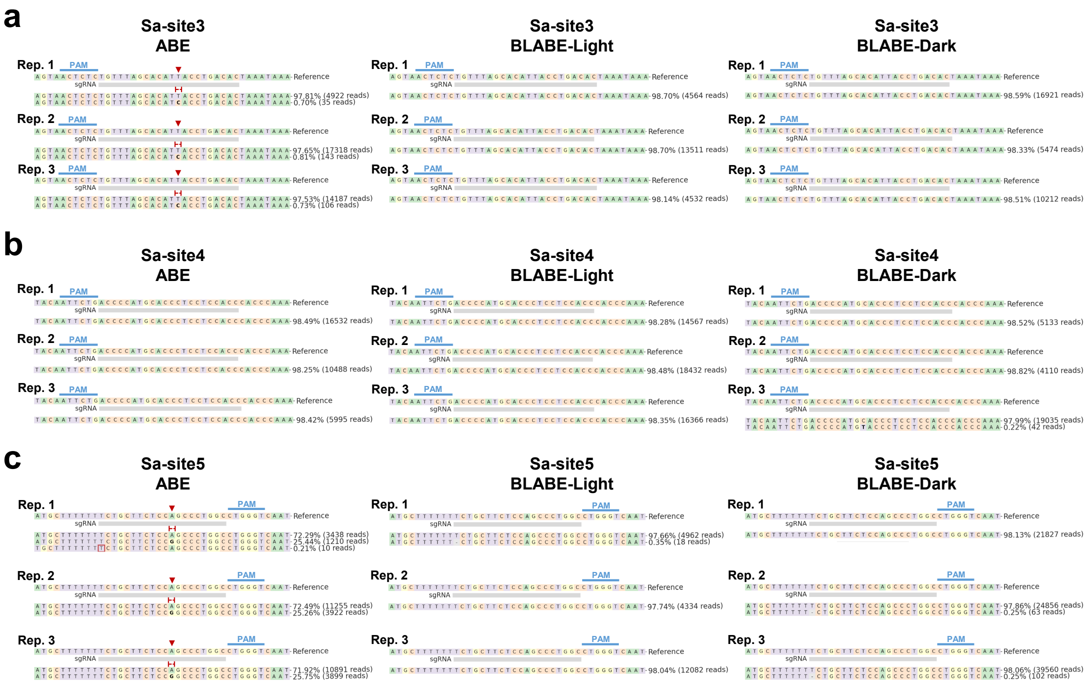

Figure S11. Allele frequencies in the amplicon of sgRNA-independent off-target editing at diverse genomic loci for ABE and BLABE systems. a) Allele frequencies of sgRNA-independent off-target for ABE and BLABE at the R-loop region named Sa-site3. b) Allele frequencies of sgRNA-independent off-target for ABE and BLABE at the R-loop region named Sa-site4. c) Allele frequencies of sgRNA-independent off-target for ABE and BLABE at the R-loop region named Sa-site5. The grey rectangle indicates the sequence of protospacer within sgRNA. BLABE system was treated under blue light and darkness respectively. The allele frequencies of off-target within the R-loop region are calculated by amplicon sequencing. The values on the right of the graph represent frequencies and mutation alleles' reads. The possible editing sites within the protospacer sequence are marked by red arrows and the unexpected editing are indicated by black arrows. The base substitutions and deletions are represented with bold letters and short dashes, respectively. The editing window is indicated by red double-ended dotted lines. PAM sequences for SpCas9 (NGG) and SaCas9 (NNGRRT) are marked by short blue lines. The values on the right of the graph represent frequencies and mutation alleles' reads (n = 3).

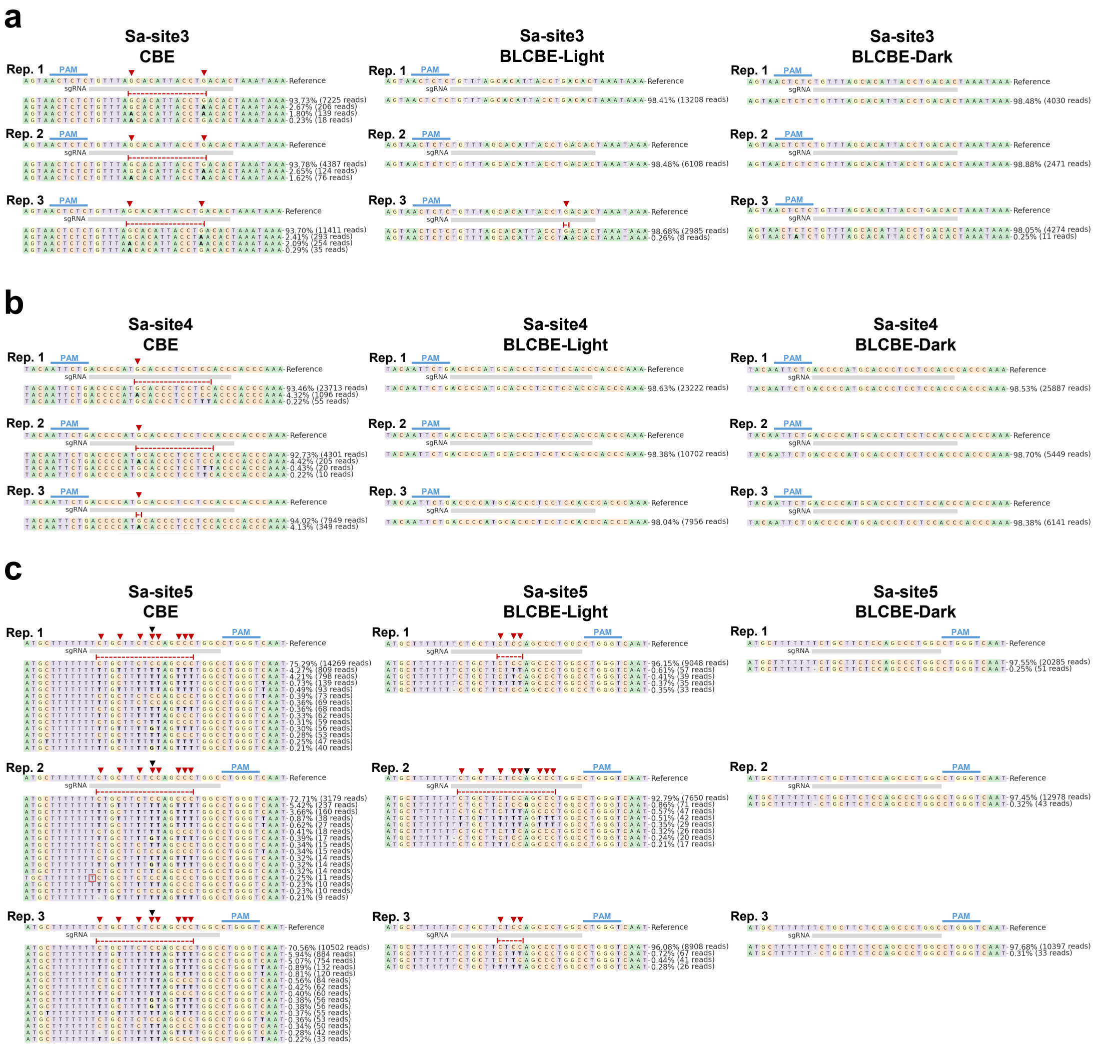

Figure S12. Allele frequencies in the amplicon of sgRNA-independent off-target editing at diverse genomic loci for CBE and BLCBE systems. a) Allele frequencies of sgRNA-independent off-target for CBE and BLCBE at the R-loop region named Sa-site3. b) Allele frequencies of sgRNA-independent off-target for CBE and BLCBE at the R-loop region named Sa-site4. c) Allele frequencies of sgRNA-independent off-target for CBE and BLCBE at the R-loop region named Sa-site5. The grey rectangle indicates the sequence of protospacer within sgRNA. The BLCBE system was treated under blue light and darkness respectively. The allele frequencies of off-target within the R-loop region are calculated by amplicon sequencing. The values on the right of the graph represent frequencies and mutation alleles' reads. The base substitutions and insertion are represented with bold letters and red box, respectively. The unexpected editing is indicated by black arrows. The editing window is indicated by red double-ended dotted lines. PAM sequences for SpCas9 (NGG) and SaCas9 (NNGRRT) are marked by short blue lines. The values on the right of the graph represent frequencies and mutation alleles' reads (n = 3).

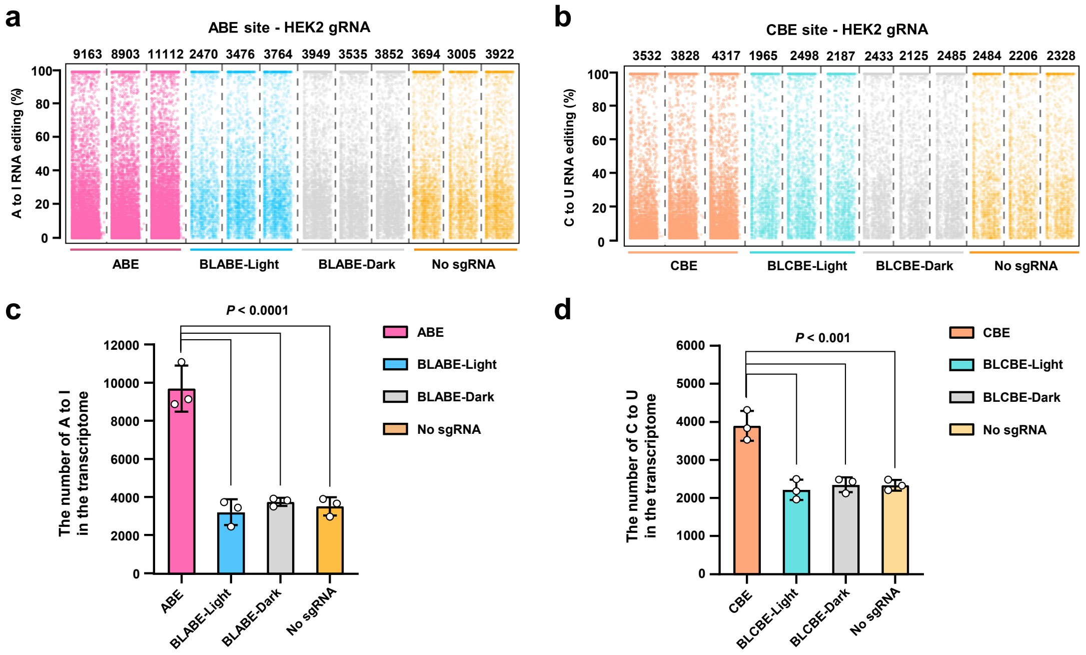

Figure S13. Off-target editing of base editor systems on the transcriptome in HEK293T cells. a) Manhattan scatter plot showing transcriptomic A-to-I mutations detected in RNA-seq experiments from HEK293T cells in which ABE, BLABE-Light, BLABE-Dark with sgRNA targeting HEK2. The HEK293T cells are transfected by ABE system which has no sgRNA as the negative control. The number of adenines modified is indicated at the top. b) Manhattan scatter plot showing transcriptomic C-to-U mutations detected in RNA-seq experiments from HEK293T cells in which ABE, BLABE-Light, BLABE-Dark with sgRNA targeting HEK2. The HEK293T cells are transfected by empty plasmid which has no sgRNA and base editor system as the negative control. The number of adenines modified is indicated at the top. c) The numbers of A to I edits in the transcriptome are shown. One-way ANOVA was performed ($P < 0.0001$). d) The numbers of C to U edits in the transcriptome are shown. One-way ANOVA was performed ($P < 0.001$). All data are shown from three independent replicates ($n = 3$).

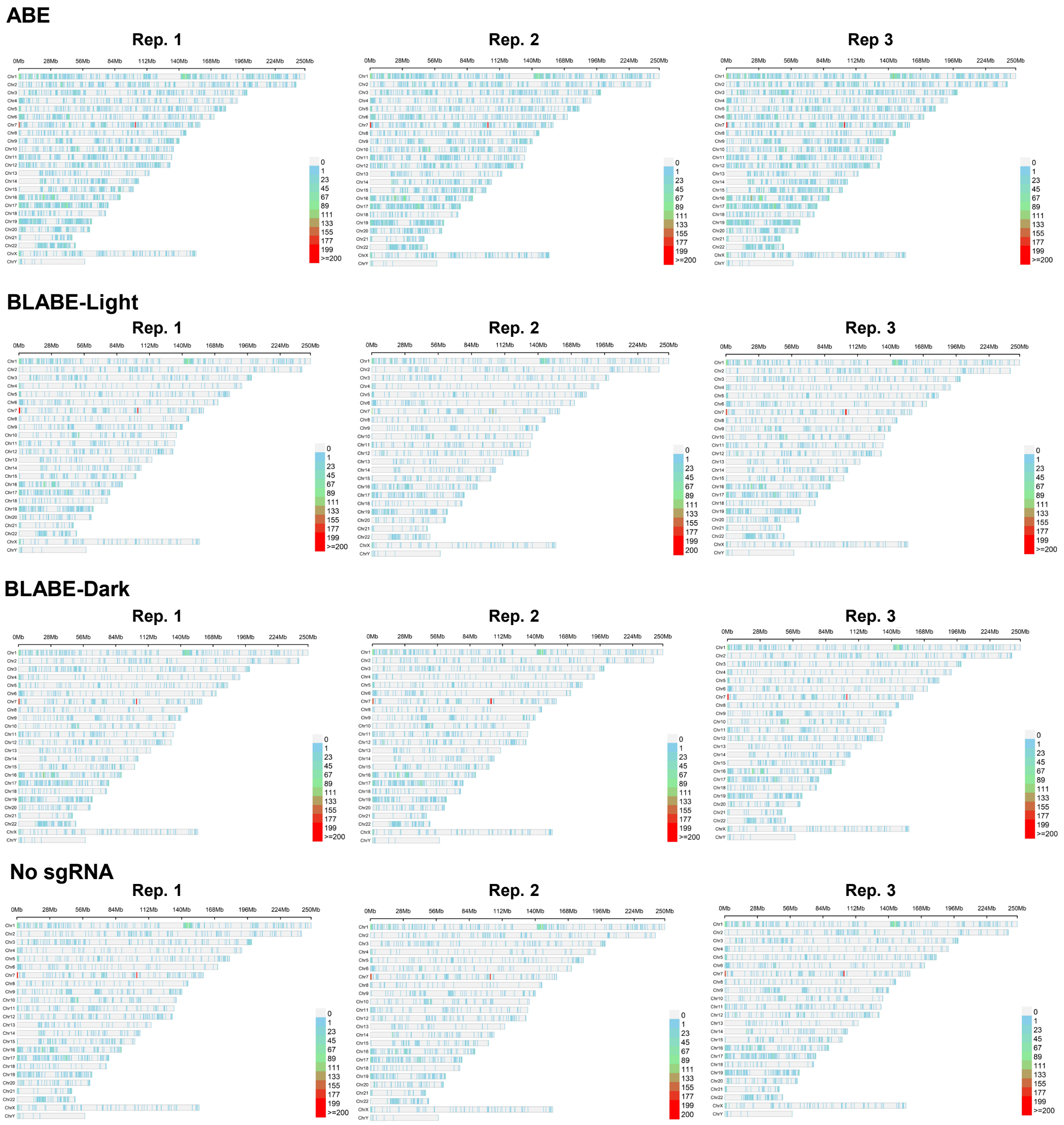

Figure S14. RNA off-target editing induced by ABE and BLABE at all chromosome locations. The SNP density plot within the HEK293T cells chromosomes is plotted from the data of transcriptome RNA SNP loci calling to the genome. SNP density plot showing base mutations that can be induced by ABE and BLABE systems. The short bar indicates the position of SNPs, and the color represents the density of SNPs in the region. The horizontal coordinate represents the chromosome physical length and the vertical coordinate represents the chromosome designation. The BLABE system is treated under blue light and darkness respectively. The HEK293T cells are transfected by empty plasmid which has no sgRNA and base editor system as the negative control. All data are shown from three independent replicates (n = 3).

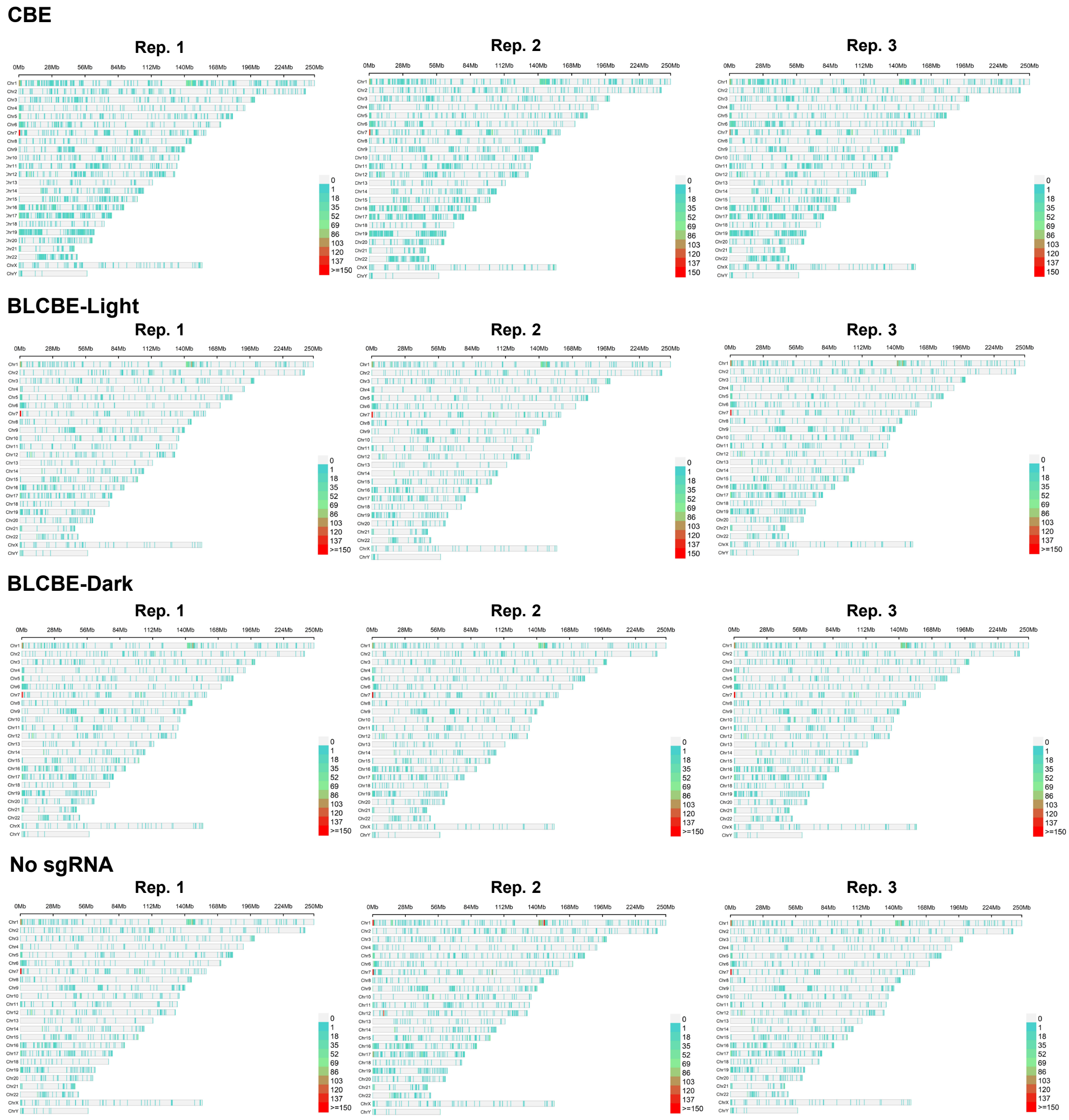

Figure S15. RNA off-target editing induced by CBE and BLCBE at all chromosome locations. The SNP density plot within the HEK293T cells chromosomes is plotted from the data of transcriptome RNA SNP loci calling to the genome. SNP density plot showing base mutations that can be induced by CBE and BLCBE systems. The short bar indicates the position of SNPs, and the color represents the density of SNPs in the region. The horizontal coordinate represents the chromosome physical length and the vertical coordinate represents the chromosome designation. The BLABE system is treated under blue light and darkness respectively. The HEK293T cells are transfected by empty plasmid which has no sgRNA and base editor system as the negative control. All data are shown from three independent replicates (*n* = 3).

Supporting Tables

Table S1. Target sgRNA-protospacer sequence for *E. coli* DH10B and HEK293T in this study.

sgRNA	Target sgRNA-protospacer sequence	PAM	Oligo primer sequence (5'→3')	
ABES1	TTGCGAATACGCCCACGCGA	TGG	sgRNA-F	GCACTTGCGAATACGCCCACGCGA
			sgRNA-R	AAACTCGCGTGGGCGTATTCGCAA
ABES2	TCAATGATATACCGTAGATT	CGG	sgRNA-F	GCACTCAATGATATACCGTAGATT
			sgRNA-R	AAACAATCTACGGTATATCATTGA
ABES3	CTGTAACGATCAATTTCTTC	AGG	sgRNA-F	GCACCTGTAACGATCAATTTCTTC
			sgRNA-R	AAACGAAGAAATTGATCGTTACAG
ABES4	CTGTACCGGAATCGCTTTCG	CGG	sgRNA-F	GCACCTGTACCGGAATCGCTTTCG
			sgRNA-R	AAACCGAAAGCGATTCCGGTACAG
ABES5	ACGACTTTATATCTTAAGAA	CGG	sgRNA-F	GCACACGACTTTATATCTTAAGAA
			sgRNA-R	AAACTTCTTAAGATATAAAGTCGT
ABES6	AACAATCAAGCAACCTGTAC	CGG	sgRNA-F	GCACAACAATCAAGCAACCTGTAC
			sgRNA-R	AAACGTACAGGTTGCTTGATTGTT
ABES7	ACGCGAAATACGGGCAGACA	TGG	sgRNA-F	GCACACGCGAAATACGGGCAGACA
			sgRNA-R	AAACTGTCTGCCCGTATTTCGCGT
ABES8	GGGGTATACATGTCTGACAA	TGG	sgRNA-F	GCACGGGGTATACATGTCTGACAA
			sgRNA-R	AAACTTGTCAGACATGTATACCCC
ABES9	GGTCAAAACAGGCGGCAGTA	AGG	sgRNA-F	GCACGGTCAAAACAGGCGGCAGTA
			sgRNA-R	AAACTACTGCCGCCTGTTTTGACC
ABES10	TGGAATTCCGCCGATACTGA	CGG	sgRNA-F	GCACTGGAATTCCGCCGATACTGA
			sgRNA-R	AAACTCAGTATCGGCGGAATTCCA
ABES11	GGCACTTCACCGCTTGCCAG	CGG	sgRNA-F	GCACGGCACTTCACCGCTTGCCAG
			sgRNA-R	AAACCTGGCAAGCGGTGAAGTGCC
ABES12	CGTTATCGCTATGACGGAAC	AGG	sgRNA-F	GCACCGTTATCGCTATGACGGAAC
			sgRNA-R	AAACGTTCCGTCATAGCGATAACG
ABES13	CAAGACTGTTACCCATCGCG	TGG	sgRNA-F	GCACCAAGACTGTTACCCATCGCG
			sgRNA-R	AAACCGCGATGGGTAACAGTCTTG
ABES14	GAAGCAGAAGCCTGCGATGT	CGG	sgRNA-F	GCACGAAGCAGAAGCCTGCGATGT
			sgRNA-R	AAACACATCGCAGGCTTCTGCTTC
ABES15	ACGTACCGCGAAAGCGATTC	CGG	sgRNA-F	GCACACGTACCGCGAAAGCGATTC
			sgRNA-R	AAACGAATCGCTTTCGCGGTACGT
ABES16	GATGACAATATCGCCGACAC	TGG	sgRNA-F	GCACGATGACAATATCGCCGACAC
			sgRNA-R	AAACGTGTCGGCGATATTGTCATC
ABES17	GCGAGCTTCTTCATCTGGCA	TGG	sgRNA-F	GCACGCGAGCTTCTTCATCTGGCA
			sgRNA-R	AAACTGCCAGATGAAGAAGCTCGC
ABES18	CATCCACGCGCGCGTACATC	GGG	sgRNA-F	GCACCATCCACGCGCGCGTACATC
			sgRNA-R	AAACGATGTACGCGCGCGTGGATG
ABES19	ATCAGCACCGCCAGCGGCGT	TGG	sgRNA-F	GCACATCAGCACCGCCAGCGGCGT
			sgRNA-R	AAACACGCCGCTGGCGGTGCTGAT

CBES1	AGCCCATGTCGCCAGTGCAA	AGG	sgRNA-F	GCACAGCCCATGTCGCCAGTGCAA
			sgRNA-R	AAACTTGCACTGGCGACATGGGCT
CBES2	ATGACCAGTATGTTCAGCTG	CGG	sgRNA-F	GCACATGACCAGTATGTTCAGCTG
			sgRNA-R	AAACCAGCTGAACATACTGGTCAT
CBES3	GTTCATCAATACTCAGCGCC	AGG	sgRNA-F	GCACGTTCATCAATACTCAGCGCC
			sgRNA-R	AAACGGCGCTGAGTATTGATGAAC
CBES4	ATCAGCACCGCCAGCGGCGT	TGG	sgRNA-F	GCACATCAGCACCGCCAGCGGCGT
			sgRNA-R	AAACACGCCGCTGGCGGTGCTGAT
CBES5	TTTTTCCAGTTCCGTTTATC	CGG	sgRNA-F	GCACTTTTTCCAGTTCCGTTTATC
			sgRNA-R	AAACGATAAACGGAACTGGAAAAA
CBES6	TGTCGCCGGTGGTTTTGCGC	TGG	sgRNA-F	GCACTGTCGCCGGTGGTTTTGCGC
			sgRNA-R	AAACGCGCAAAACCACCGGCGACA
CBES7	CGACTTCTACTGGCGCATCC	GGG	sgRNA-F	GCACCGACTTCTACTGGCGCATCC
			sgRNA-R	AAACGGATGCGCCAGTAGAAGTCG
CBES8	GCTGCACCCAGAAATCCATA	GGG	sgRNA-F	GCACGCTGCACCCAGAAATCCATA
			sgRNA-R	AAACTATGGATTTCTGGGTGCAGC
CBES9	GATCGCCATGCTGGTGGCGC	AGG	sgRNA-F	GCACGATCGCCATGCTGGTGGCGC
			sgRNA-R	AAACGCGCCACCAGCATGGCGATC
CBES10	AATCTCTTAACCGCGTTGCC	GGG	sgRNA-F	GCACAATCTCTTAACCGCGTTGCC
			sgRNA-R	AAACGGCAACGCGGTTAAGAGATT
CBES11	GAACCGGTAATCGCCACAAT	CGG	sgRNA-F	GCACGAACCGGTAATCGCCACAAT
			sgRNA-R	AAACATTGTGGCGATTACCGGTTC
CBES12	CATCATCAACGATATTCTGG	CGG	sgRNA-F	GCACCATCATCAACGATATTCTGG
			sgRNA-R	AAACCCAGAATATCGTTGATGATG
EXM1	GAGTCCGAGCAGAAGAAGAA	GGG	sgRNA-F	CAGCTCAGCCTGAGTGTTGA
			sgRNA-R	CGATGTCCTCCCCATTGGCCTGC
HEK site2	GAACACAAAGCATAGACTGC	GGG	sgRNA-F	GGTCCTAAACCAGTGTCAGGGAGC
			sgRNA-R	TGCTCCCCTCAGCATTCAGCCA
HEK site3	GGCCCAGACTGAGCACGTGA	TGG	sgRNA-F	ATGTGGGCTGCCTAGAAAGG
			sgRNA-R	CCCAGCCAAACTTGTCAACC
ABE site2	GAGTATGAGGCATAGACTGC	AGG	sgRNA-F	CCCTGAGATACAGTCACGAGGTAA
			sgRNA-R	TGAGGTCAGAAGTTTGAGACCAGC
ABE site15	GTCTAGAAAGCTTAGACTGC	TGG	sgRNA-F	ATGCCAGGTCAGATCCTGGG
			sgRNA-R	GGGGTTAGTAACGGATGACATAG

Table S2. Target sgRNA-protospacer sequence for *E. coli* DH10B and HEK293T in this study.

Name	Amplicon target sequence	PAM	Oligo primers for HTS (5’→3’)	
ABE ABES4-Target	CTGTACCGGAATCGCTTTCG	CGG	F-primer	ATATAACCCCGGGGGATAATCCGTTGTTAC
			R-primer	GGGATCTCGCCAGCTTCGTTACCATGCC
ABE ABES4-OT1	CTGTACCGTTATCGCTTTCG	TGG	F-primer	GGATACATGCCAAGCTGAATACCCAGCA
			R-primer	CGTAAGACGACAGCCTGTTACAGGCAGC
ABE ABES19-Target	ATCAGCACCGCCAGCGGCGT	TGG	F-primer	TCATGGGACGCTGAAAGCGAAGCCTGCC
			R-primer	CTACAACTATGGCGGCACTACGTGAACC
ABE ABES19-OT1	ATCAACGCCACCAGCGGCGT	GAG	F-primer	CGTCGAATGCGAATTTGATTGCGGTGCC
			R-primer	CTTTGACGCGAGGTGTGTGGCGATCTCT
ABE ABES19-OT2	TCCAGCAACGCCTGCGGCGT	CGG	F-primer	CTCGCGAAGAACGTGACGCCCACCAGTA
			R-primer	GCTGCCAGGTTTGCGACACGCCGGTCAC
ABE ABES19-OT3	TTCGACACCGCCACCGGCGT	TAG	F-primer	CAGGCGAAACTCAACAGCGAGGAAGACGTC
			R-primer	GAGTATCAGTATTCGCATGGTGGTTGACTGT
ABE ABES19-OT4	GACAGCAGCGCCTGCGGCGT	CGG	F-primer	CAGCTTAGGCAATAACACCGTCTTACCGTC
			R-primer	GAATCTAGATGAAGTGGGTGAAGTGCGC
BLABE ABES4-Target	CTGTACCGGAATCGCTTTCG	CGG	F-primer	AGCATATTCCGGGGGATAATCCGTTGTTAC
			R-primer	CCTAGTTGGCCAGCTTCGTTACCATGCC
BLABE ABES4-OT1	CTGTACCGTTATCGCTTTCG	TGG	F-primer	CGGAGTTCGCCAAGCTGAATACCCAGCA
			R-primer	TCCGCATTGACAGCCTGTTACAGGCAGC
BLABE ABES19-Target	ATCAGCACCGCCAGCGGCGT	TGG	F-primer	ATCAAGATCGCTGAAAGCGAAGCCTGCC
			R-primer	CGTTGGTTATGGCGGCACTACGTGAACC
BLABE ABES19-OT1	ATCAACGCCACCAGCGGCGT	GAG	F-primer	CTAACACGGCGAATTTGATTGCGGTGCC
			R-primer	CTTACCGGCGAGGTGTGTGGCGATCTCT
BLABE ABES19-OT2	GACAGCAGCGCCTGCGGCGT	CGG	F-primer	TGAGATCAGAACGTGACGCCCACCAGTA
			R-primer	CTTACGTGGTTTGCGACACGCCGGTCAC
BLABE ABES19-OT3	TCCAGCAACGCCTGCGGCGT	CGG	F-primer	CTCTAGGCACTCAACAGCGAGGAAGACGTC
			R-primer	CACCGATCGTATTCGCATGGTGGTTGACTGT

BLABE ABES19-OT4	TTCGACACCGCCACCGGCGT	CAG	F-primer	CACACGCCGCAATAACACCGTCTTACCGTC
			R-primer	TTGCGACCATGAAGTGGGTGAAGTGCGC
CBE CBES4-Target	ATCAGCACCGCCAGCGGCGT	TGG	F-primer	GTACAGTACGCTGAAAGCGAAGCCTGCC
			R-primer	GCATGTGCATGGCGGCACTACGTGAACC
CBE CBES4-OT1	ATCAACGCCACCAGCGGCGT	GAG	F-primer	GTCACCAAGCGAATTTGATTGCGGTGCC
			R-primer	TGATGTTGCGAGGTGTGTGGCGATCTCT
CBE CBES4-OT2	GACAGCAGCGCCTGCGGCGT	CGG	F-primer	GGCTCGCTGAACGTGACGCCCACCAGTA
			R-primer	TCCTGTTCGTTTGCGACACGCCGGTCAC
CBE CBES4-OT3	TCCAGCAACGCCTGCGGCGT	CGG	F-primer	TGGATTACACTCAACAGCGAGGAAGACGTC
			R-primer	AGTACGTGGTATTCGCATGGTGGTTGACTGT
CBE CBES4-OT4	TTCGACACCGCCACCGGCGT	CAG	F-primer	GAGCATCAGCAATAACACCGTCTTACCGTC
			R-primer	AACTGCTAATGAAGTGGGTGAAGTGCGC
BLCBE CBES4-Target	ATCAGCACCGCCAGCGGCGT	TGG	F-primer	CGCTTATACGCTGAAAGCGAAGCCTGCC
			R-primer	GCACATGAATGGCGGCACTACGTGAACC
BLCBE CBES4-OT1	ATCAACGCCACCAGCGGCGT	GAG	F-primer	CATAGGTGGCGAATTTGATTGCGGTGCC
			R-primer	TCTAGACTCGAGGTGTGTGGCGATCTCT
BLCBE CBES4-OT2	GACAGCAGCGCCTGCGGCGT	CGG	F-primer	GCTCGGTTGAACGTGACGCCCACCAGTA
			R-primer	ATAGTGGCGTTTGCGACACGCCGGTCAC
BLCBE CBES4-OT3	TCCAGCAACGCCTGCGGCGT	CGG	F-primer	GACTAAGAACTCAACAGCGAGGAAGACGTC
			R-primer	TAGCTGGCGTATTCGCATGGTGGTTGACTGT
BLCBE CBES4-OT4	TTCGACACCGCCACCGGCGT	CAG	F-primer	TAGTTGTGGCAATAACACCGTCTTACCGTC
			R-primer	ATTAGTACATGAAGTGGGTGAAGTGCGC
CBE CBES6-Target	TGTCGCCGGTGGTTTTGCGC	TGG	F-primer	CACGGCCTGGGGCTGTTCTACATTCTGCTG
			R-primer	TAGTGATGATCGCGGTACAGACAATAACCAG
CBE CBES6-OT1	TTTACCCGCTGGTTTTGCGC	AAG	F-primer	GGATCCGTTCTCGCTTAAGCCTGGTGCC
			R-primer	TAGTATGAGAGCACAGTCAACAGCAGCA
BLCBE CBES6-Target	TGTCGCCGGTGGTTTTGCGC	TGG	F-primer	CAGACGTGGGGGCTGTTCTACATTCTGCTG
			R-primer	GTAGGACTATCGCGGTACAGACAATAACCAG

CBE CBES6-OT1	TTTACCCGCTGGTTTTGCGC	AAG	F-primer	GGATCCGTTCTCGCTTAAGCCTGGTGCC
			R-primer	TAGTATGAGAGCACAGTCAACAGCAGCA
BLCBE CBES6-Target	TGTCGCCGGTGGTTTTGCGC	TGG	F-primer	CAGACGTGGGGGCTGTTCTACATTCTGCTG
			R-primer	GTAGGACTATCGCGGTACAGACAATAACCAG
BLCBE CBES6-OT1	TTTACCCGCTGGTTTTGCGC	AAG	F-primer	TACTCAACTCTCGCTTAAGCCTGGTGCC
			R-primer	TAGTAGTTGAGCACAGTCAACAGCAGCA
ABE R-loop1	TGAATATCGACGGTTTCCATAT	GGGGAT	F-primer	AAGGCAGTGACATGGCCTGCCCGGTTATTA
			R-primer	GTCAGCCTACATCAGCCGCTACAGTCAAC
ABE R-loop2	CGTTTTCGCTCGGGAAGACGTA	CGGGGA	F-primer	GAAGGACTGCTGTTGACTGTAGCGGCTG
			R-primer	AACTTGGCCAGAGCGGGTAAACTGGCTC
ABE R-loop3	CAATGTCGTTATCCAGCGGTGC	ACGGGT	F-primer	GGTCGGTGAACAACGCTGCTTCGGCCTG
			R-primer	TGGTTGTCCGAGCTGGGTAATAAGCGTTGGC
BLABE R-loop1	TGAATATCGACGGTTTCCATAT	GGGGAT	F-primer	CTGATCCCGACATGGCCTGCCCGGTTATTA
			R-primer	AGGAACGTACATCAGCCGCTACAGTCAAC
BLABE R-loop2	CGTTTTCGCTCGGGAAGACGTA	CGGGGT	F-primer	CAACGCATGCTGTTGACTGTAGCGGCTG
			R-primer	ATCTGAATCAGAGCGGGTAAACTGGCTC
BLABE R-loop3	CAATGTCGTTATCCAGCGGTGC	ACGGGT	F-primer	GACCTTGTAACAACGCTGCTTCGGCCTG
			R-primer	CGTCGTACCGAGCTGGGTAATAAGCGTTGGC
CBE R-loop1	TGAATATCGACGGTTTCCATAT	GGGGAT	F-primer	ACCGTCGCGACATGGCCTGCCCGGTTATTA
			R-primer	AATAGACGACATCAGCCGCTACAGTCAAC
CBE R-loop2	CGTTTTCGCTCGGGAAGACGTA	CGGGGT	F-primer	CCTGATGCGCTGTTGACTGTAGCGGCTG
			R-primer	TGCTCGTACAGAGCGGGTAAACTGGCTC
CBE R-loop3	CAATGTCGTTATCCAGCGGTGC	ACGGGT	F-primer	AATCGTAGAACAACGCTGCTTCGGCCTG
			R-primer	TGTTCGACCGAGCTGGGTAATAAGCGTTGGC
BLCBE R-loop1	TGAATATCGACGGTTTCCATAT	GGGGAT	F-primer	TGAGCGGAGACATGGCCTGCCCGGTTATTA
			R-primer	AAGGACAAACATCAGCCGCTACAGTCAAC
BLCBE R-loop2	CGTTTTCGCTCGGGAAGACGTA	CGGGGT	F-primer	ACAGACGTGCTGTTGACTGTAGCGGCTG
			R-primer	TATGCGGCCAGAGCGGGTAAACTGGCTC
BLCBE R-loop3	CAATGTCGTTATCCAGCGGTGC	ACGGGT	F-primer	GACGCCTCAACAACGCTGCTTCGGCCTG
			R-primer	TTGACACTCGAGCTGGGTAATAAGCGTTGGC

ABE *HEK* site2-OT1	GAACACAATGCATAGATTGC	CGG	F-primer	CAGCTTAGGTGTGGAGAGTGAGTAAGCCA
			R-primer	GAATCTAGACGGTAGGATGATTTCAGGCA
BLABE *HEK* site2-OT1	GAACACAATGCATAGATTGC	CGG	F-primer	CAACGTTGGTGTGGAGAGTGAGTAAGCCA
			R-primer	GATGAGGAACGGTAGGATGATTTCAGGCA
CBE *EXM1*-OT1	GAGTCTAAGCAGAAGAAGAA	GAG	F-primer	GTACAGTAAGTAGCCTCTTTCTCAATGTGC
			R-primer	GCATGTGCGCTTTCACAAGGATGCAGTCT
BLCBE *EXM1*-OT1	GAGTCTAAGCAGAAGAAGAA	GAG	F-primer	GAACCGTGAGTAGCCTCTTTCTCAATGTGC
			R-primer	TGTTGCACGCTTTCACAAGGATGCAGTCT
CBE *HEK* site2-OT1	GAACACAATGCATAGATTGC	CGG	F-primer	GACAGGATGTGTGGAGAGTGAGTAAGCCA
			R-primer	CGATATCTACGGTAGGATGATTTCAGGCA
BLCBE *HEK* site2-OT1	GAACACAATGCATAGATTGC	CGG	F-primer	TCATGGTCGTGTGGAGAGTGAGTAAGCCA
			R-primer	CCTACATCACGGTAGGATGATTTCAGGCA
CBE *HEK* site3-OT1	CACCCAGACTGAGCACGTGC	TGG	F-primer	GTCACCAATCCCCTGTTGACCTGGAGAA
			R-primer	TGATGTTGCACTGTACTTGCCCTGACCA
BLCBE *HEK* site3-OT1	CACCCAGACTGAGCACGTGC	TGG	F-primer	TGGATTACTCCCCTGTTGACCTGGAGAA
			R-primer	AGTACGTGCACTGTACTTGCCCTGACCA
ABE Sa site-3	GTGTCAGGTAATGTGCTAAACA	GAGAGT	F-primer	CAGCATGCCTGCACCTAGCCTCCATGTC
			R-primer	CGGTAACCGTGCTGTGGCATCCAGAGACAT
ABE Sa site-4	GGTGGAGGAGGGTGCATGGGGT	CAGAAT	F-primer	GGATACATGATCCTGAGGTCTAGGAACCCG
			R-primer	CGTAAGACGGAGGTGGAGAGAGGATGT
ABE Sa site-5	TCTGCTTCTCCAGCCCTGGC	CTGGGT	F-primer	TCATGGGAATGTGGGCTGCCTAGAAAGG
			R-primer	CTACAACTCCCAGCCAAACTTGTCAACC
BLABE Sa site-3	GTGTCAGGTAATGTGCTAAACA	GAGAGT	F-primer	TGACCTTCCTGCACCTAGCCTCCATGTC
			R-primer	ACACATCGGTGCTGTGGCATCCAGAGACAT
BLABE Sa site-4	GGTGGAGGAGGGTGCATGGGGT	CAGAAT	F-primer	AGGCCTACGATCCTGAGGTCTAGGAACCCG
			R-primer	AGATGTTCGGAGGTGGAGAGAGGATGT
BLABE Sa site-5	TCTGCTTCTCCAGCCCTGGC	CTGGGT	F-primer	AGACATGAATGTGGGCTGCCTAGAAAGG
			R-primer	TCTCCGCACCCAGCCAAACTTGTCAACC

ABE<br>Sa site-6	GATGTTCCAATCAGTACGCA	GAGAGT	F-primer	CGTCGAATGGGGTCCCAGGTGCTGAC
			R-primer	CTTTGACGCATTGCAGAGAGGCGTATC
BLABE<br>Sa site-6	GATGTTCCAATCAGTACGCA	GAGAGT	F-primer	CAGGCGAAGGGGTCCCAGGTGCTGAC
			R-primer	GAGTATCACATTGCAGAGAGGCGTATC
CBE<br>Sa site-3	GTGTCAGGTAATGTGCTAAACA	GAGAGT	F-primer	ACTGTTAGCTGCACCTAGCCTCCATGTC
			R-primer	TGACATGGGTGCTGTGGCATCCAGAGACAT
CBE<br>Sa site-4	GGTGGAGGAGGGTGCATGGGGT	CAGAAT	F-primer	CACGGCCTGATCCTGAGGTCTAGGAACCCG
			R-primer	TAGTGATGGGAGGTGGAGAGAGGATGT
CBE<br>Sa site-5	TCTGCTTCTCCAGCCCTGGC	CTGGGT	F-primer	GGATCCGTATGTGGGCTGCCTAGAAAGG
			R-primer	TAGTATGACCCAGCCAAACTTGTCAACC
CBE<br>Sa site-6	GATGTTCCAATCAGTACGCA	GAGAGT	F-primer	ATATAACCGGGGTCCCAGGTGCTGAC
			R-primer	GGGATCTCCATTGCAGAGAGGCGTATC
BLCBE<br>Sa site-3	GTGTCAGGTAATGTGCTAAACA	GAGAGT	F-primer	CAGTCAGCCTGCACCTAGCCTCCATGTC
			R-primer	CCGATCGAGTGCTGTGGCATCCAGAGACAT
BLCBE<br>Sa site-4	GGTGGAGGAGGGTGCATGGGGT	CAGAAT	F-primer	GTGACTAAGATCCTGAGGTCTAGGAACCCG
			R-primer	TAACAGTCGGAGGTGGAGAGAGGATGT
BLCBE<br>Sa site-5	TCTGCTTCTCCAGCCCTGGC	CTGGGT	F-primer	GCAGCATTATGTGGGCTGCCTAGAAAGG
			R-primer	TCATAATCCCCAGCCAAACTTGTCAACC
BLCBE<br>Sa site-6	GATGTTCCAATCAGTACGCA	GAGAGT	F-primer	CAGCGCTCGGGGTCCCAGGTGCTGAC
			R-primer	AGGATCAGCATTGCAGAGAGGCGTATC

Supporting Sequences

Sequence S1. Plasmids (functional amino acid sequences shown only) for *E. coli* DH10B in this study.

pEcA8e (A8e-linker-nCas9)

MSEVEFSHEYWMRHALTLAKRARDEREVPVGAVLVLNNRVIGEGWLRAIGLHDPTAHAEIMALRQ
GGLVMQNYRLIDATLYVTFEPCVMCAGAMIHSRIGRVVFGVRNSKRGAAGSLMNVLNYPGMNHRV
EITEGILADECAALLCDFYRMPRQVFNAQKKAQSSINSGGSSGGSSGSETPGTSESATPESSGGS
SGGSDKKYSIGLAIGTNSVGWAVITDEYKVPSKKFKVLGNTDRHSIKKNLIGALLFDSGETAEAT
RLKRTARRRYTRRKNRICYLQEIFSNEMAKVDDSFFHRLEESFLVEEDKKHERHPIFGNIVDEVA
YHEKYPTIYHLRKKLVDSTDKADLRLIYLALAHMIKFRGHFLIEGDLNPDNSDVDKLFIQLVQTY
NQLFEENPINASGVDAKAILSARLSKSRRLENLIAQLPGEKKNGLFGNLIALSLGLTPNFKSNFD
LAEDAKLQLSKDTYDDDLDNLLAQIGDQYADLFLAAKNLSDAILLSDILRVNTEITKAPLSASMI
KRYDEHHQDLTLLKALVRQQLPEKYKEIFFDQSKNGYAGYIDGGASQEEFYKFIKPILEKMDGTE
ELLVKLNREDLLRKQRTFDNGSIPHQIHLGELHAILRRQEDFYPFLKDNREKIEKILTFRIPYYV
GPLARGNSRFAWMTRKSEETITPWNFEEVVDKGASAQSFIERMTNFDKNLPNEKVLPKHSLLYEY
FTVYNELTKVKYVTEGMRKPAFLSGEQKKAIVDLLFKTNRKVTVKQLKEDYFKKIECFDSVEISG
VEDRFNASLGTYHDLLKIIKDKDFLDNEENEDILEDIVLTLTLFEDREMIEERLKTYAHLFDDKV
MKQLKRRRYTGWGRLSRKLINGIRDKQSGKTILDFLKSDGFANRNFMQLIHDDSLTFKEDIQKAQ
VSGQGDSLHEHIANLAGSPAIKKGILQTVKVVDELVKVMGRHKPENIVIEMARENQTTQKGQKNS
RERMKRIEEGIKELGSQILKEHPVENTQLQNEKLYLYYLQNGRDMYVDQELDINRLSDYDVDHIV
PQSFLKDDSIDNKVLTRSDKNRGKSDNVPSEEVVKKMKNYWRQLLNAKLITQRKFDNLTKAERGG
LSELDKAGFIKRQLVETRQITKHVAQILDSRMNTKYDENDKLIREVKVITLKSKLVSDFRKDFQF
YKVREINNYHHAHDAYLNAVVGTALIKKYPKLESEFVYGDYKVYDVRKMIAKSEQEIGKATAKYF
FYSNIMNFFKTEITLANGEIRKRPLIETNGETGEIVWDKGRDFATVRKVLSMPQVNIVKKTEVQT
GGFSKESILPKRNSDKLIARKKDWDPKKYGGFDSPTVAYSVLVVAKVEKGKSKKLKSVKELLGIT
IMERSSFEKNPIDFLEAKGYKEVKKDLIIKLPKYSLFELENGRKRMLASAGELQKGNELALPSKY
VNFLYLASHYEKLKGSPEDNEQKQLFVEQHKHYLDEIIEQISEFSKRVILADANLDKVLSAYNKH
RDKPIREQAENIIHLFTLTNLGAPAAFKYFDTTIDRKRYTSTKEVLDATLIHQSITGLYETRIDL
SQLGGD*

pEcBLABE (N-A8e-linker-nMagHigh-RBS-pMag-linker-C-A8e-linker-nCas9)

MSEVEFSHEYWMRHALTLAKRARDEREVPVGAVLVLNNRVIGEGWNRAIGLHDPTAHAEIMALRQ
GGLVMQNYRGGSGGSGGSGHTLYAPGGYDIMGYLDQIGNRPNPQVELGPVDTSCALILCDLKQKD
TPIVYASEAFLYMTGYSNAEVLGRNCRFLQSPDGMVKPKSTRKYVDSNTINTIRKAIDRNAEVQV
EVVNFKKNGQRFVNFLTIIPVRDETGEYRYSMGFQCETE*gaattcattaaagaggagaaaggtc
atMHTLYAPGGYDIMGYLRQIRNRPNPQVELGPVDTSCALILCDLKQKDTPVVYASEAFLYMTGY
SNAEVLGRNCRFLQSPDGMVKPKSTRKYVDSNTINTMRKAIDRNAEVQVEVVNFKKNGQRFVNFL
TMIPVRDETGEYRYSMGFQCETEGSGSGSGSGSLIDATLYVTFEPCVMCAGAMIHSRIGRVVFGV
RNSKRGAAGSLMNVLNYPGMNHRVEITEGILADECAALLCDFYRMPRQVFNAQKKAQSSINSGGS
SGGSSGSETPGTSESATPESSGGSSGGSDKKYSIGLAIGTNSVGWAVITDEYKVPSKKFKVLGNT
DRHSIKKNLIGALLFDSGETAEATRLKRTARRRYTRRKNRICYLQEIFSNEMAKVDDSFFHRLEE
SFLVEEDKKHERHPIFGNIVDEVAYHEKYPTIYHLRKKLVDSTDKADLRLIYLALAHMIKFRGHF

LIEGDLNPDNSDVDKLFIQLVQTYNQLFEENPINASGVDAKAILSARLSKSRRLENLIAQLPGEK
KNGLFGNLIALSLGLTPNFKSNFDLAEDAKLQLSKDTYDDDLDNLLAQIGDQYADLFLAAKNLSD
AILLSDILRVNTEITKAPLSASMIKRYDEHHQDLTLLKALVRQQLPEKYKEIFFDQSKNGYAGYI
DGGASQEEFYKFIKPILEKMDGTEELLVKLNREDLLRKQRTFDNGSIPHQIHLGELHAILRRQED
FYPFLKDNREKIEKILTFRIPYYVGPLARGNSRFAWMTRKSEETITPWNFEEVVDKGASAQSFIE
RMTNFDKNLPNEKVLPKHSLLYEYFTVYNELTKVKYVTEGMRKPAFLSGEQKKAIVDLLFKTNRK
VTVKQLKEDYFKKIECFDSVEISGVEDRFNASLGTYHDLLKIIKDKDFLDNEENEDILEDIVLTL
TLFEDREMIEERLKTYAHLFDDKVMKQLKRRRYTGWGRLSRKLINGIRDKQSGKTILDFLKSDGF
ANRNFMQLIHDDSLTFKEDIQKAQVSGQGDSLHEHIANLAGSPAIKKGILQTVKVVDELVKVMGR
HKPENIVIEMARENQTTQKGQKNSRERMKRIEEGIKELGSQILKEHPVENTQLQNEKLYLYYLQN
GRDMYVDQELDINRLSDYDVDHIVPQSFLKDDSIDNKVLTRSDKNRGKSDNVPSEEVVKKMKNYW
RQLLNAKLITQRKFDNLTKAERGGLSELDKAGFIKRQLVETRQITKHVAQILDSRMNTKYDENDK
LIREVKVITLKSKLVSDFRKDFQFYKVREINNYHHAHDAYLNAVVGTALIKKYPKLESEFVYGDY
KVYDVRKMIAKSEQEIGKATAKYFFYSNIMNFFKTEITLANGEIRKRPLIETNGETGEIVWDKGR
DFATVRKVLSMPQVNIVKKTEVQTGGFSKESILPKRNSDKLIARKKDWDPKKYGGFDSPTVAYSV
LVVAKVEKGKSKKLKSVKELLGITIMERSSFEKNPIDFLEAKGYKEVKKDLIIKLPKYSLFELEN
GRKRMLASAGELQKGNELALPSKYVNFLYLASHYEKLKGSPEDNEQKQLFVEQHKHYLDEIIEQI
SEFSKRVILADANLDKVLSAYNKHRDKPIREQAENIIHLFTLTNLGAPAAFKYFDTTIDRKRYTS
TKEVLDATLIHQSITGLYETRIDLSQLGGD*

pEcA3A (A3A-linker-nCas9-linker-UGI)

MEASPASGPRHLMDPHIFTSNFNNGIGRHKTYLCYEVERLDNGTSVKMDQHRGFLHNQAKNLLCG
FYGRHAELRFLDLVPSLQLDAQIYRVTWFISWSPCFSWGCAGEVRAFLQENTHVRLRIFAARIYD
YDPLYKEALQMLRDAGAQVSIMTYDEFKHCWDTFVDHQGCPFQPWDGLDEHSQALSGRLRAILQN
QGNSGSETPGTSESATPESDKKYSIGLAIGTNSVGWAVITDEYKVPSKKFKVLGNTDRHSIKKNL
IGALLFDSGETAEATRLKRTARRRYTRRKNRICYLQEIFSNEMAKVDDSFFHRLEESFLVEEDKK
HERHPIFGNIVDEVAYHEKYPTIYHLRKKLVDSTDKADLRLIYLALAHMIKFRGHFLIEGDLNPD
NSDVDKLFIQLVQTYNQLFEENPINASGVDAKAILSARLSKSRRLENLIAQLPGEKKNGLFGNLI
ALSLGLTPNFKSNFDLAEDAKLQLSKDTYDDDLDNLLAQIGDQYADLFLAAKNLSDAILLSDILR
VNTEITKAPLSASMIKRYDEHHQDLTLLKALVRQQLPEKYKEIFFDQSKNGYAGYIDGGASQEEF
YKFIKPILEKMDGTEELLVKLNREDLLRKQRTFDNGSIPHQIHLGELHAILRRQEDFYPFLKDNR
EKIEKILTFRIPYYVGPLARGNSRFAWMTRKSEETITPWNFEEVVDKGASAQSFIERMTNFDKNL
PNEKVLPKHSLLYEYFTVYNELTKVKYVTEGMRKPAFLSGEQKKAIVDLLFKTNRKVTVKQLKED
YFKKIECFDSVEISGVEDRFNASLGTYHDLLKIIKDKDFLDNEENEDILEDIVLTLTLFEDREMI
EERLKTYAHLFDDKVMKQLKRRRYTGWGRLSRKLINGIRDKQSGKTILDFLKSDGFANRNFMQLI
HDDSLTFKEDIQKAQVSGQGDSLHEHIANLAGSPAIKKGILQTVKVVDELVKVMGRHKPENIVIE
MARENQTTQKGQKNSRERMKRIEEGIKELGSQILKEHPVENTQLQNEKLYLYYLQNGRDMYVDQE
LDINRLSDYDVDHIVPQSFLKDDSIDNKVLTRSDKNRGKSDNVPSEEVVKKMKNYWRQLLNAKLI
TQRKFDNLTKAERGGLSELDKAGFIKRQLVETRQITKHVAQILDSRMNTKYDENDKLIREVKVIT
LKSKLVSDFRKDFQFYKVREINNYHHAHDAYLNAVVGTALIKKYPKLESEFVYGDYKVYDVRKMI
AKSEQEIGKATAKYFFYSNIMNFFKTEITLANGEIRKRPLIETNGETGEIVWDKGRDFATVRKVL
SMPQVNIVKKTEVQTGGFSKESILPKRNSDKLIARKKDWDPKKYGGFDSPTVAYSVLVVAKVEKG
KSKKLKSVKELLGITIMERSSFEKNPIDFLEAKGYKEVKKDLIIKLPKYSLFELENGRKRMLASA
GELQKGNELALPSKYVNFLYLASHYEKLKGSPEDNEQKQLFVEQHKHYLDEIIEQISEFSKRVIL

ADANLDKVLSAYNKHRDKPIREQAENIIHLFTLTNLGAPAAFKYFDTTIDRKRYTSTKEVLDATL
IHQSITGLYETRIDLSQLGGDSGGSTNLSDIIEKETGKQLVIQESILMLPEEVEEVIGNKPESDI
LVHTAYDESTDENVMLLTSDAPEYKPWALVIQDSNGENKIKML*

pEcBLCBE (N-A3A-linker-nMagHigh-RBS-pMag-linker-C-A3A-linker-nCas9-linker-UGI)
MEASPASGPRHLMDPHIFTSNFNNGIGRHKTYLCYEVERLDNGTSVKMDQHRGFLHNQAKNLLCG
FYGRHAELRFLDLVPSLQLDGGSGGSGGSGHTLYAPGGYDIMGYLDQIGNRPNPQVELGPVDTSC
ALILCDLKQKDTPIVYASEAFLYMTGYSNAEVLGRNCRFLQSPDGMVKPKSTRKYVDSNTINTIR
KAIDRNAEVQVEVVNFKKNGQRFVNFLTIIPVRDETGEYRYSMGFQCETE*gaattcattaaaga
ggagaaaggtcatMHTLYAPGGYDIMGYLRQIRNRPNPQVELGPVDTSCALILCDLKQKDTPVVY
ASEAFLYMTGYSNAEVLGRNCRFLQSPDGMVKPKSTRKYVDSNTINTMRKAIDRNAEVQVEVVNF
KKNGQRFVNFLTMIPVRDETGEYRYSMGFQCETEGSGSGSGSGSPAQIYRVTWFISWSPCFSWGC
AGEVRAFLQENTHVRLRIFAARIYDYDPLYKEALQMLRDAGAQVSIMTYDEFKHCWDTFVDHQGC
PFQPWDGLDEHSQALSGRLRAILQNQGNSGSETPGTSESATPESDKKYSIGLAIGTNSVGWAVIT
DEYKVPSKKFKVLGNTDRHSIKKNLIGALLFDSGETAEATRLKRTARRRYTRRKNRICYLQEIFS
NEMAKVDDSFFHRLEESFLVEEDKKHERHPIFGNIVDEVAYHEKYPTIYHLRKKLVDSTDKADLR
LIYLALAHMIKFRGHFLIEGDLNPDNSDVDKLFIQLVQTYNQLFEENPINASGVDAKAILSARLS
KSRRLENLIAQLPGEKKNGLFGNLIALSLGLTPNFKSNFDLAEDAKLQLSKDTYDDDLDNLLAQI
GDQYADLFLAAKNLSDAILLSDILRVNTEITKAPLSASMIKRYDEHHQDLTLLKALVRQQLPEKY
KEIFFDQSKNGYAGYIDGGASQEEFYKFIKPILEKMDGTEELLVKLNREDLLRKQRTFDNGSIPH
QIHLGELHAILRRQEDFYPFLKDNREKIEKILTFRIPYYVGPLARGNSRFAWMTRKSEETITPWN
FEEVVDKGASAQSFIERMTNFDKNLPNEKVLPKHSLLYEYFTVYNELTKVKYVTEGMRKPAFLSG
EQKKAIVDLLFKTNRKVTVKQLKEDYFKKIECFDSVEISGVEDRFNASLGTYHDLLKIIKDKDFL
DNEENEDILEDIVLTLTLFEDREMIEERLKTYAHLFDDKVMKQLKRRRYTGWGRLSRKLINGIRD
KQSGKTILDFLKSDGFANRNFMQLIHDDSLTFKEDIQKAQVSGQGDSLHEHIANLAGSPAIKKGI
LQTVKVVDELVKVMGRHKPENIVIEMARENQTTQKGQKNSRERMKRIEEGIKELGSQILKEHPVE
NTQLQNEKLYLYYLQNGRDMYVDQELDINRLSDYDVDHIVPQSFLKDDSIDNKVLTRSDKNRGKS
DNVPSEEVVKKMKNYWRQLLNAKLITQRKFDNLTKAERGGLSELDKAGFIKRQLVETRQITKHVA
QILDSRMNTKYDENDKLIREVKVITLKSKLVSDFRKDFQFYKVREINNYHHAHDAYLNAVVGTAL
IKKYPKLESEFVYGDYKVYDVRKMIAKSEQEIGKATAKYFFYSNIMNFFKTEITLANGEIRKRPL
IETNGETGEIVWDKGRDFATVRKVLSMPQVNIVKKTEVQTGGFSKESILPKRNSDKLIARKKDWD
PKKYGGFDSPTVAYSVLVVAKVEKGKSKKLKSVKELLGITIMERSSFEKNPIDFLEAKGYKEVKK
DLIIKLPKYSLFELENGRKRMLASAGELQKGNELALPSKYVNFLYLASHYEKLKGSPEDNEQKQL
FVEQHKHYLDEIIEQISEFSKRVILADANLDKVLSAYNKHRDKPIREQAENIIHLFTLTNLGAPA
AFKYFDTTIDRKRYTSTKEVLDATLIHQSITGLYETRIDLSQLGGDSGGSTNLSDIIEKETGKQL
VIQESILMLPEEVEEVIGNKPESDILVHTAYDESTDENVMLLTSDAPEYKPWALVIQDSNGENKI
KML*

pdSaCas9 (dSaCas9)
KRNYILGLDIGITSVGYGIIDYETRDVIDAGVRLFKEANVENNEGRRSKRGARRLKRRRRHRIQR
VKKLLFDYNLLTDHSELSGINPYEARVKGLSQKLSEEEFSAALLHLAKRRGVHNVNEVEEDTGNE
LSTKEQISRNSKALEEKYVAELQLERLKKDGEVRGSINRFKTSDYVKEAKQLLKVQKAYHQLDQS
FIDTYIDLLETRRTYYEGPGEGSPFGWKDIKEWYEMLMGHCTYFPEELRSVKYAYNADLYNALND
LNNLVITRDENEKLEYYEKFQIIENVFKQKKKPTLKQIAKEILVNEEDIKGYRVTSTGKPEFTNL

KVYHDIKDITARKEIIENAELLDQIAKILTIYQSSEDIQEELTNLNSELTQEEIEQISNLKGYTG
THNLSLKAINLILDELWHTNDNQIAIFNRLKLVPKKVDLSQQKEIPTTLVDDFILSPVVKRSFIQ
SIKVINAIIKKYGLPNDIIIELAREKNSKDAQKMINEMQKRNRQTNERIEEIIRTTGKENAKYLI
EKIKLHDMQEGKCLYSLEAIPLEDLLNNPFNYEVDHIIPRSVSFDNSFNNKVLVKQEENSKKGNR
TPFQYLSSSDSKISYETFKKHILNLAKGKGRISKTKKEYLLEERDINRFSVQKDFINRNLVDTRY
ATRGLMNLLRSYFRVNNLDVKVKSINGGFTSFLRRKWKFKKERNKGYKHHAEDALIIANADFIFK
EWKKLDKAKKVMENQMFEEKQAESMPEIETEQEYKEIFITPHQIKHIKDFKDYKYSHRVDKKPNR
ELINDTLYSTRKDDKGNTLIVNNLNGLYDKDNDKLKKLINKSPEKLLMYHHDPQTYQKLKLIMEQ
YGDEKNPLYKYYEETGNYLTKYSKKDNGPVIKKIKYYGNKLNAHLDITDDYPNSRNKVVKLSLKP
YRFDVYLDNGVYKFVTVKNLDVIKKENYYEVNSKCYEEAKKLKKISNQAEFIASFYNNDLIKING
ELYRVIGVNNDLLNRIEVNMIDITYREYLENMNDKRPPRIIKTIASKTQSIKKYSTDILGNLYEV
KSKKHPQIIKKG*

Sequences S2. Plasmids (functional amino acid sequences shown only) for HEK293T in this study.

pCMV-A8e (A8e-linker-nCas9-NLS)

MSEVEFSHEYWMRHALTLAKRARDEREVPVGAVLVLNNRVIGEGWLRAIGLHDPTAHAEIMALRQ
GGLVMQNYRLIDATLYVTFEPCVMCAGAMIHSRIGRVVFGVRNSKRGAAGSLMNVLNYPGMNHRV
EITEGILADECAALLCDFYRMPRQVFNAQKKAQSSINSGGSSGGSSGSETPGTSESATPESSGGS
SGGSDKKYSIGLAIGTNSVGWAVITDEYKVPSKKFKVLGNTDRHSIKKNLIGALLFDSGETAEAT
RLKRTARRRYTRRKNRICYLQEIFSNEMAKVDDSFFHRLEESFLVEEDKKHERHPIFGNIVDEVA
YHEKYPTIYHLRKKLVDSTDKADLRLIYLALAHMIKFRGHFLIEGDLNPDNSDVDKLFIQLVQTY
NQLFEENPINASGVDAKAILSARLSKSRRLENLIAQLPGEKKNGLFGNLIALSLGLTPNFKSNFD
LAEDAKLQLSKDTYDDDLDNLLAQIGDQYADLFLAAKNLSDAILLSDILRVNTEITKAPLSASMI
KRYDEHHQDLTLLKALVRQQLPEKYKEIFFDQSKNGYAGYIDGGASQEEFYKFIKPILEKMDGTE
ELLVKLNREDLLRKQRTFDNGSIPHQIHLGELHAILRRQEDFYPFLKDNREKIEKILTFRIPYYV
GPLARGNSRFAWMTRKSEETITPWNFEEVVDKGASAQSFIERMTNFDKNLPNEKVLPKHSLLYEY
FTVYNELTKVKYVTEGMRKPAFLSGEQKKAIVDLLFKTNRKVTVKQLKEDYFKKIECFDSVEISG
VEDRFNASLGTYHDLLKIIKDKDFLDNEENEDILEDIVLTLTLFEDREMIEERLKTYAHLFDDKV
MKQLKRRRYTGWGRLSRKLINGIRDKQSGKTILDFLKSDGFANRNFMQLIHDDSLTFKEDIQKAQ
VSGQGDSLHEHIANLAGSPAIKKGILQTVKVVDELVKVMGRHKPENIVIEMARENQTTQKGQKNS
RERMKRIEEGIKELGSQILKEHPVENTQLQNEKLYLYYLQNGRDMYVDQELDINRLSDYDVDHIV
PQSFLKDDSIDNKVLTRSDKNRGKSDNVPSEEVVKKMKNYWRQLLNAKLITQRKFDNLTKAERGG
LSELDKAGFIKRQLVETRQITKHVAQILDSRMNTKYDENDKLIREVKVITLKSKLVSDFRKDFQF
YKVREINNYHHAHDAYLNAVVGTALIKKYPKLESEFVYGDYKVYDVRKMIAKSEQEIGKATAKYF
FYSNIMNFFKTEITLANGEIRKRPLIETNGETGEIVWDKGRDFATVRKVLSMPQVNIVKKTEVQT
GGFSKESILPKRNSDKLIARKKDWDPKKYGGFDSPTVAYSVLVVAKVEKGKSKKLKSVKELLGIT
IMERSSFEKNPIDFLEAKGYKEVKKDLIIKLPKYSLFELENGRKRMLASAGELQKGNELALPSKY
VNFLYLASHYEKLKGSPEDNEQKQLFVEQHKHYLDEIIEQISEFSKRVILADANLDKVLSAYNKH
RDKPIREQAENIIHLFTLTNLGAPAAFKYFDTTIDRKRYTSTKEVLDATLIHQSITGLYETRIDL
SQLGGDSGGSPKKKRKV*

pCMV- A8eN (NLS-N-A8e-linker-nMagHigh)

MKRTADGSEFESPKKKRKVGGSSEVEFSHEYWMRHALTLAKRARDEREVPVGAVLVLNNRVIGEG
WNRAIGLHDPTAHAEIMALRQGGLVMQNYRGGSGGSGGSGHTLYAPGGYDIMGYLDQIGNRPNPQ
VELGPVDTSCALILCDLKQKDTPIVYASEAFLYMTGYSNAEVLGRNCRFLQSPDGMVKPKSTRKY
VDSNTINTIRKAIDRNAEVQVEVVNFKKNGQRFVNFLTIIPVRDETGEYRYSMGFQCETE*

pCMV-pMag-A8eC (pMag-linker-C-A8e-linker-nCas9-NLS)

MHTLYAPGGYDIMGYLRQIRNRPNPQVELGPVDTSCALILCDLKQKDTPVVYASEAFLYMTGYSN
AEVLGRNCRFLQSPDGMVKPKSTRKYVDSNTINTMRKAIDRNAEVQVEVVNFKKNGQRFVNFLTM
IPVRDETGEYRYSMGFQCETEGSGSGSGSGSLIDATLYVTFEPCVMCAGAMIHSRIGRVVFGVRN
SKRGAAGSLMNVLNYPGMNHRVEITEGILADECAALLCDFYRMPRQVFNAQKKAQSSINSGGSSG
GSSGSDKKYSIGLAIGTNSVGWAVITDEYKVPSKKFKVLGNTDRHSIKKNLIGALLFDSGETAEA
TRLKRTARRRYTRRKNRICYLQEIFSNEMAKVDDSFFHRLEESFLVEEDKKHERHPIFGNIVDEV
AYHEKYPTIYHLRKKLVDSTDKADLRLIYLALAHMIKFRGHFLIEGDLNPDNSDVDKLFIQLVQT
YNQLFEENPINASGVDAKAILSARLSKSRRLENLIAQLPGEKKNGLFGNLIALSLGLTPNFKSNF

DLAEDAKLQLSKDTYDDDLDNLLAQIGDQYADLFLAAKNLSDAILLSDILRVNTEITKAPLSASM
IKRYDEHHQDLTLLKALVRQQLPEKYKEIFFDQSKNGYAGYIDGGASQEEFYKFIKPILEKMDGT
EELLVKLNREDLLRKQRTFDNGSIPHQIHLGELHAILRRQEDFYPFLKDNREKIEKILTFRIPYY
VGPLARGNSRFAWMTRKSEETITPWNFEEVVDKGASAQSFIERMTNFDKNLPNEKVLPKHSLLYE
YFTVYNELTKVKYVTEGMRKPAFLSGEQKKAIVDLLFKTNRKVTVKQLKEDYFKKIECFDSVEIS
GVEDRFNASLGTYHDLLKIIKDKDFLDNEENEDILEDIVLTLTLFEDREMIEERLKTYAHLFDDK
VMKQLKRRRYTGWGRLSRKLINGIRDKQSGKTILDFLKSDGFANRNFMQLIHDDSLTFKEDIQKA
QVSGQGDSLHEHIANLAGSPAIKKGILQTVKVVDELVKVMGRHKPENIVIEMARENQTTQKGQKN
SRERMKRIEEGIKELGSQILKEHPVENTQLQNEKLYLYYLQNGRDMYVDQELDINRLSDYDVDHI
VPQSFLKDDSIDNKVLTRSDKNRGKSDNVPSEEVVKKMKNYWRQLLNAKLITQRKFDNLTKAERG
GLSELDKAGFIKRQLVETRQITKHVAQILDSRMNTKYDENDKLIREVKVITLKSKLVSDFRKDFQ
FYKVREINNYHHAHDAYLNAVVGTALIKKYPKLESEFVYGDYKVYDVRKMIAKSEQEIGKATAKY
FFYSNIMNFFKTEITLANGEIRKRPLIETNGETGEIVWDKGRDFATVRKVLSMPQVNIVKKTEVQ
TGGFSKESILPKRNSDKLIARKKDWDPKKYGGFDSPTVAYSVLVVAKVEKGKSKKLKSVKELLGI
TIMERSSFEKNPIDFLEAKGYKEVKKDLIIKLPKYSLFELENGRKRMLASAGELQKGNELALPSK
YVNFLYLASHYEKLKGSPEDNEQKQLFVEQHKHYLDEIIEQISEFSKRVILADANLDKVLSAYNK
HRDKPIREQAENIIHLFTLTNLGAPAAFKYFDTTIDRKRYTSTKEVLDATLIHQSITGLYETRID
LSQLGGDSGGSPKKKRKV*

pCMV-A3A (A3A-linker-nCas9-linker-UGI-NLS)
MEASPASGPRHLMDPHIFTSNFNNGIGRHKTYLCYEVERLDNGTSVKMDQHRGFLHNQAKNLLCG
FYGRHAELRFLDLVPSLQLDAQIYRVTWFISWSPCFSWGCAGEVRAFLQENTHVRLRIFAARIYD
YDPLYKEALQMLRDAGAQVSIMTYDEFKHCWDTFVDHQGCPFQPWDGLDEHSQALSGRLRAILQN
QGNSGSETPGTSESATPESDKKYSIGLAIGTNSVGWAVITDEYKVPSKKFKVLGNTDRHSIKKNL
IGALLFDSGETAEATRLKRTARRRYTRRKNRICYLQEIFSNEMAKVDDSFFHRLEESFLVEEDKK
HERHPIFGNIVDEVAYHEKYPTIYHLRKKLVDSTDKADLRLIYLALAHMIKFRGHFLIEGDLNPD
NSDVDKLFIQLVQTYNQLFEENPINASGVDAKAILSARLSKSRRLENLIAQLPGEKKNGLFGNLI
ALSLGLTPNFKSNFDLAEDAKLQLSKDTYDDDLDNLLAQIGDQYADLFLAAKNLSDAILLSDILR
VNTEITKAPLSASMIKRYDEHHQDLTLLKALVRQQLPEKYKEIFFDQSKNGYAGYIDGGASQEEF
YKFIKPILEKMDGTEELLVKLNREDLLRKQRTFDNGSIPHQIHLGELHAILRRQEDFYPFLKDNR
EKIEKILTFRIPYYVGPLARGNSRFAWMTRKSEETITPWNFEEVVDKGASAQSFIERMTNFDKNL
PNEKVLPKHSLLYEYFTVYNELTKVKYVTEGMRKPAFLSGEQKKAIVDLLFKTNRKVTVKQLKED
YFKKIECFDSVEISGVEDRFNASLGTYHDLLKIIKDKDFLDNEENEDILEDIVLTLTLFEDREMI
EERLKTYAHLFDDKVMKQLKRRRYTGWGRLSRKLINGIRDKQSGKTILDFLKSDGFANRNFMQLI
HDDSLTFKEDIQKAQVSGQGDSLHEHIANLAGSPAIKKGILQTVKVVDELVKVMGRHKPENIVIE
MARENQTTQKGQKNSRERMKRIEEGIKELGSQILKEHPVENTQLQNEKLYLYYLQNGRDMYVDQE
LDINRLSDYDVDHIVPQSFLKDDSIDNKVLTRSDKNRGKSDNVPSEEVVKKMKNYWRQLLNAKLI
TQRKFDNLTKAERGGLSELDKAGFIKRQLVETRQITKHVAQILDSRMNTKYDENDKLIREVKVIT
LKSKLVSDFRKDFQFYKVREINNYHHAHDAYLNAVVGTALIKKYPKLESEFVYGDYKVYDVRKMI
AKSEQEIGKATAKYFFYSNIMNFFKTEITLANGEIRKRPLIETNGETGEIVWDKGRDFATVRKVL
SMPQVNIVKKTEVQTGGFSKESILPKRNSDKLIARKKDWDPKKYGGFDSPTVAYSVLVVAKVEKG
KSKKLKSVKELLGITIMERSSFEKNPIDFLEAKGYKEVKKDLIIKLPKYSLFELENGRKRMLASA
GELQKGNELALPSKYVNFLYLASHYEKLKGSPEDNEQKQLFVEQHKHYLDEIIEQISEFSKRVIL
ADANLDKVLSAYNKHRDKPIREQAENIIHLFTLTNLGAPAAFKYFDTTIDRKRYTSTKEVLDATL

IHQSITGLYETRIDLSQLGGDSGGSTNLSDIIEKETGKQLVIQESILMLPEEVEEVIGNKPESDI
LVHTAYDESTDENVMLLTSDAPEYKPWALVIQDSNGENKIKMLSGGSPKKKRKV*

pCMV-A3AN (NLS-N-A3A-linker-nMagHigh)

MKRTADGSEFESPKKKRKVGGSEASPASGPRHLMDPHIFTSNFNNGIGRHKTYLCYEVERLDNGT
SVKMDQHRGFLHNQAKNLLCGFYGRHAELRFLDLVPSLQLDGGSGGSGGSGHTLYAPGGYDIMGY
LDQIGNRPNPQVELGPVDTSCALILCDLKQKDTPIVYASEAFLYMTGYSNAEVLGRNCRFLQSPD
GMVKPKSTRKYVDSNTINTIRKAIDRNAEVQVEVVNFKKNGQRFVNFLTIIPVRDETGEYRYSMG
FQCETE*

pCMV-pMag-A3AC (pMag-linker-C-A3A-linker-nCas9-linker-UGI-NLS)

MHTLYAPGGYDIMGYLRQIRNRPNPQVELGPVDTSCALILCDLKQKDTPVVYASEAFLYMTGYSN
AEVLGRNCRFLQSPDGMVKPKSTRKYVDSNTINTMRKAIDRNAEVQVEVVNFKKNGQRFVNFLTM
IPVRDETGEYRYSMGFQCETEGSGSGSGSGSPAQIYRVTWFISWSPCFSWGCAGEVRAFLQENTH
VRLRIFAARIYDYDPLYKEALQMLRDAGAQVSIMTYDEFKHCWDTFVDHQGCPFQPWDGLDEHSQ
ALSGRLRAILQNQGNSGSETPGTSESATPESDKKYSIGLAIGTNSVGWAVITDEYKVPSKKFKVL
GNTDRHSIKKNLIGALLFDSGETAEATRLKRTARRRYTRRKNRICYLQEIFSNEMAKVDDSFFHR
LEESFLVEEDKKHERHPIFGNIVDEVAYHEKYPTIYHLRKKLVDSTDKADLRLIYLALAHMIKFR
GHFLIEGDLNPDNSDVDKLFIQLVQTYNQLFEENPINASGVDAKAILSARLSKSRRLENLIAQLP
GEKKNGLFGNLIALSLGLTPNFKSNFDLAEDAKLQLSKDTYDDDLDNLLAQIGDQYADLFLAAKN
LSDAILLSDILRVNTEITKAPLSASMIKRYDEHHQDLTLLKALVRQQLPEKYKEIFFDQSKNGYA
GYIDGGASQEEFYKFIKPILEKMDGTEELLVKLNREDLLRKQRTFDNGSIPHQIHLGELHAILRR
QEDFYPFLKDNREKIEKILTFRIPYYVGPLARGNSRFAWMTRKSEETITPWNFEEVVDKGASAQS
FIERMTNFDKNLPNEKVLPKHSLLYEYFTVYNELTKVKYVTEGMRKPAFLSGEQKKAIVDLLFKT
NRKVTVKQLKEDYFKKIECFDSVEISGVEDRFNASLGTYHDLLKIIKDKDFLDNEENEDILEDIV
LTLTLFEDREMIEERLKTYAHLFDDKVMKQLKRRRYTGWGRLSRKLINGIRDKQSGKTILDFLKS
DGFANRNFMQLIHDDSLTFKEDIQKAQVSGQGDSLHEHIANLAGSPAIKKGILQTVKVVDELVKV
MGRHKPENIVIEMARENQTTQKGQKNSRERMKRIEEGIKELGSQILKEHPVENTQLQNEKLYLYY
LQNGRDMYVDQELDINRLSDYDVDHIVPQSFLKDDSIDNKVLTRSDKNRGKSDNVPSEEVVKKMK
NYWRQLLNAKLITQRKFDNLTKAERGGLSELDKAGFIKRQLVETRQITKHVAQILDSRMNTKYDE
NDKLIREVKVITLKSKLVSDFRKDFQFYKVREINNYHHAHDAYLNAVVGTALIKKYPKLESEFVY
GDYKVYDVRKMIAKSEQEIGKATAKYFFYSNIMNFFKTEITLANGEIRKRPLIETNGETGEIVWD
KGRDFATVRKVLSMPQVNIVKKTEVQTGGFSKESILPKRNSDKLIARKKDWDPKKYGGFDSPTVA
YSVLVVAKVEKGKSKKLKSVKELLGITIMERSSFEKNPIDFLEAKGYKEVKKDLIIKLPKYSLFE
LENGRKRMLASAGELQKGNELALPSKYVNFLYLASHYEKLKGSPEDNEQKQLFVEQHKHYLDEII
EQISEFSKRVILADANLDKVLSAYNKHRDKPIREQAENIIHLFTLTNLGAPAAFKYFDTTIDRKR
YTSTKEVLDATLIHQSITGLYETRIDLSQLGGDSGGSTNLSDIIEKETGKQLVIQESILMLPEEV
EEVIGNKPESDILVHTAYDESTDENVMLLTSDAPEYKPWALVIQDSNGENKIKMLSGGSPKKKRK
V*

pCMV-IRES-BLCBE (N-A3A-linker-nMagHigh-IRES-pMag-linker-C-A3A-linker- nCas9-4aa-UGI-NLS)

MEASPASGPRHLMDPHIFTSNFNNGIGRHKTYLCYEVERLDNGTSVKMDQHRGFLHNQAKNLLCG
FYGRHAELRFLDLVPSLQLDGGSGGSGGSGHTLYAPGGYDIMGYLDQIGNRPNPQVELGPVDTSC

ALILCDLKQKDTPIVYASEAFLYMTGYSNAEVLGRNCRFLQSPDGMVKPKSTRKYVDSNTINTIR
KAIDRNAEVQVEVVNFKKNGQRFVNFLTIIPVRDETGEYRYSMGFQCETETAATGACCCCCCCC
CTAACGTTACTGGCCGAAGCCGCTTGGAATAAGGCCGGTGTGCGTTTGTCTATATGTTATTTTCC
ACCATATTGCCGTCTTTTGGCAATGTGAGGGCCCGGAAACCTGGCCCTGTCTTCTTGACGAGCAT
TCCTAGGGGTCTTTCCCCTCTCGCCAAAGGAATGCAAGGTCTGTTGAATGTCGTGAAGGAAGCAG
TTCCTCTGGAAGCTTCTTGAAGACAAACAACGTCTGTAGCGACCCTTTGCAGGCAGCGGAACCCC
CCACCTGGCGACAGGTGCCTCTGCGGCCAAAAGCCACGTGTATAAGATACACCTGCAAAGGCGGC
ACAACCCCAGTGCCACGTTGTGAGTTGGATAGTTGTGGAAAGAGTCAAATGGCTCTCCTCAAGCG
TATTCAACAAGGGGCTGAAGGATGCCCAGAAGGTACCCCATTGTATGGGATCTGATCTGGGGCCT
CGGTGCACATGCTTTACATGTGTTTAGTCGAGGTTAAAAAAACGTCTAGGCCCCCCGAACCACGG
GGACGTGGTTTTCCTTTGAAAAACACGATGATAACCCCAAGCTTGCCACCMHTLYAPGGYDIMGY
LRQIRNRPNPQVELGPVDTSCALILCDLKQKDTPVVYASEAFLYMTGYSNAEVLGRNCRFLQSPD
GMVKPKSTRKYVDSNTINTMRKAIDRNAEVQVEVVNFKKNGQRFVNFLTMIPVRDETGEYRYSMG
FQCETEGSGSGSGSGSPAQIYRVTWFISWSPCFSWGCAGEVRAFLQENTHVRLRIFAARIYDYDP
LYKEALQMLRDAGAQVSIMTYDEFKHCWDTFVDHQGCPFQPWDGLDEHSQALSGRLRAILQNQGN
SGSETPGTSESATPESDKKYSIGLAIGTNSVGWAVITDEYKVPSKKFKVLGNTDRHSIKKNLIGA
LLFDSGETAEATRLKRTARRRYTRRKNRICYLQEIFSNEMAKVDDSFFHRLEESFLVEEDKKHER
HPIFGNIVDEVAYHEKYPTIYHLRKKLVDSTDKADLRLIYLALAHMIKFRGHFLIEGDLNPDNSD
VDKLFIQLVQTYNQLFEENPINASGVDAKAILSARLSKSRRLENLIAQLPGEKKNGLFGNLIALS
LGLTPNFKSNFDLAEDAKLQLSKDTYDDDLDNLLAQIGDQYADLFLAAKNLSDAILLSDILRVNT
EITKAPLSASMIKRYDEHHQDLTLLKALVRQQLPEKYKEIFFDQSKNGYAGYIDGGASQEEFYKF
IKPILEKMDGTEELLVKLNREDLLRKQRTFDNGSIPHQIHLGELHAILRRQEDFYPFLKDNREKI
EKILTFRIPYYVGPLARGNSRFAWMTRKSEETITPWNFEEVVDKGASAQSFIERMTNFDKNLPNE
KVLPKHSLLYEYFTVYNELTKVKYVTEGMRKPAFLSGEQKKAIVDLLFKTNRKVTVKQLKEDYFK
KIECFDSVEISGVEDRFNASLGTYHDLLKIIKDKDFLDNEENEDILEDIVLTLTLFEDREMIEER
LKTYAHLFDDKVMKQLKRRRYTGWGRLSRKLINGIRDKQSGKTILDFLKSDGFANRNFMQLIHDD
SLTFKEDIQKAQVSGQGDSLHEHIANLAGSPAIKKGILQTVKVVDELVKVMGRHKPENIVIEMAR
ENQTTQKGQKNSRERMKRIEEGIKELGSQILKEHPVENTQLQNEKLYLYYLQNGRDMYVDQELDI
NRLSDYDVDHIVPQSFLKDDSIDNKVLTRSDKNRGKSDNVPSEEVVKKMKNYWRQLLNAKLITQR
KFDNLTKAERGGLSELDKAGFIKRQLVETRQITKHVAQILDSRMNTKYDENDKLIREVKVITLKS
KLVSDFRKDFQFYKVREINNYHHAHDAYLNAVVGTALIKKYPKLESEFVYGDYKVYDVRKMIAKS
EQEIGKATAKYFFYSNIMNFFKTEITLANGEIRKRPLIETNGETGEIVWDKGRDFATVRKVLSMP
QVNIVKKTEVQTGGFSKESILPKRNSDKLIARKKDWDPKKYGGFDSPTVAYSVLVVAKVEKGKSK
KLKSVKELLGITIMERSSFEKNPIDFLEAKGYKEVKKDLIIKLPKYSLFELENGRKRMLASAGEL
QKGNELALPSKYVNFLYLASHYEKLKGSPEDNEQKQLFVEQHKHYLDEIIEQISEFSKRVILADA
NLDKVLSAYNKHRDKPIREQAENIIHLFTLTNLGAPAAFKYFDTTIDRKRYTSTKEVLDATLIHQ
SITGLYETRIDLSQLGGDSGGSTNLSDIIEKETGKQLVIQESILMLPEEVEEVIGNKPESDILVH
TAYDESTDENVMLLTSDAPEYKPWALVIQDSNGENKIKMLSGGSPKKKRKV*

pCMV-P2A-BLCBE (N-A3A-linker-nMagHigh-P2A-pMag-linker-C-A3A-linker-nCas9-linker-UGI-NLS)

MEASPASGPRHLMDPHIFTSNFNNGIGRHKTYLCYEVERLDNGTSVKMDQHRGFLHNQAKNLLCG
FYGRHAELRFLDLVPSLQLDGGSGGSGGSGHTLYAPGGYDIMGYLDQIGNRPNPQVELGPVDTSC
ALILCDLKQKDTPIVYASEAFLYMTGYSNAEVLGRNCRFLQSPDGMVKPKSTRKYVDSNTINTIR

KAIDRNAEVQVEVVNFKKNGQRFVNFLTIIPVRDETGEYRYSMGFQCETEGSGATNFSLLKQAGD
VEENPGPMHTLYAPGGYDIMGYLRQIRNRPNPQVELGPVDTSCALILCDLKQKDTPVVYASEAFL
YMTGYSNAEVLGRNCRFLQSPDGMVKPKSTRKYVDSNTINTMRKAIDRNAEVQVEVVNFKKNGQR
FVNFLTMIPVRDETGEYRYSMGFQCETEGSGSGSGSPAQIYRVTWFISWSPCFSWGCAGEVRA
FLQENTHVRLRIFAARIYDYDPLYKEALQMLRDAGAQVSIMTYDEFKHCWDTFVDHQGCPFQPWD
GLDEHSQALSGRLRAILQNQGNSGSETPGTSESATPESDKKYSIGLAIGTNSVGWAVITDEYKVP
SKKFKVLGNTDRHSIKKNLIGALLFDSGETAEATRLKRTARRRYTRRKNRICYLQEIFSNEMAKV
DDSFFHRLEESFLVEEDKKHERHPIFGNIVDEVAYHEKYPTIYHLRKKLVDSTDKADLRLIYLAL
AHMIKFRGHFLIEGDLNPDNSDVDKLFIQLVQTYNQLFEENPINASGVDAKAILSARLSKSRRLE
NLIAQLPGEKKNGLFGNLIALSLGLTPNFKSNFDLAEDAKLQLSKDTYDDDLDNLLAQIGDQYAD
LFLAAKNLSDAILLSDILRVNTEITKAPLSASMIKRYDEHHQDLTLLKALVRQQLPEKYKEIFFD
QSKNGYAGYIDGGASQEEFYKFIKPILEKMDGTEELLVKLNREDLLRKQRTFDNGSIPHQIHLGE
LHAILRRQEDFYPFLKDNREKIEKILTFRIPYYVGPLARGNSRFAWMTRKSEETITPWNFEEVVD
KGASAQSFIERMTNFDKNLPNEKVLPKHSLLYEYFTVYNELTKVKYVTEGMRKPAFLSGEQKKAI
VDLLFKTNRKVTVKQLKEDYFKKIECFDSVEISGVEDRFNASLGTYHDLLKIIKDKDFLDNEENE
DILEDIVLTLTLFEDREMIEERLKTYAHLFDDKVMKQLKRRRYTGWGRLSRKLINGIRDKQSGKT
ILDFLKSDGFANRNFMQLIHDDSLTFKEDIQKAQVSGQGDSLHEHIANLAGSPAIKKGILQTVKV
VDELVKVMGRHKPENIVIEMARENQTTQKGQKNSRERMKRIEEGIKELGSQILKEHPVENTQLQN
EKLYLYYLQNGRDMYVDQELDINRLSDYDVDHIVPQSFLKDDSIDNKVLTRSDKNRGKSDNVPSE
EVVKKMKNYWRQLLNAKLITQRKFDNLTKAERGGLSELDKAGFIKRQLVETRQITKHVAQILDSR
MNTKYDENDKLIREVKVITLKSKLVSDFRKDFQFYKVREINNYHHAHDAYLNAVVGTALIKKYPK
LESEFVYGDYKVYDVRKMIAKSEQEIGKATAKYFFYSNIMNFFKTEITLANGEIRKRPLIETNGE
TGEIVWDKGRDFATVRKVLSMPQVNIVKKTEVQTGGFSKESILPKRNSDKLIARKKDWDPKKYGG
FDSPTVAYSVLVVAKVEKGKSKKLKSVKELLGITIMERSSFEKNPIDFLEAKGYKEVKKDLIIKL
PKYSLFELENGRKRMLASAGELQKGNELALPSKYVNFLYLASHYEKLKGSPEDNEQKQLFVEQHK
HYLDEIIEQISEFSKRVILADANLDKVLSAYNKHRDKPIREQAENIIHLFTLTNLGAPAAFKYFD
TTIDRKRYTSTKEVLDATLIHQSITGLYETRIDLSQLGGDSGGSTNLSDIIEKETGKQLVIQESI
LMLPEEVEEVIGNKPESDILVHTAYDESTDENVMLLTSDAPEYKPWALVIQDSNGENKIKMLSGG
SPKKKRKV*

pCMV-dSaCas9 (dSaCas9-linker-UGI-NLS)

KRNYILGLDIGITSVGYGIIDYETRDVIDAGVRLFKEANVENNEGRRSKRGARRLKRRRRHRIQR
VKKLLFDYNLLTDHSELSGINPYEARVKGLSQKLSEEEFSAALLHLAKRRGVHNVNEVEEDTGNE
LSTKEQISRNSKALEEKYVAELQLERLKKDGEVRGSINRFKTSDYVKEAKQLLKVQKAYHQLDQS
FIDTYIDLLETRRTYYEGPGEGSPFGWKDIKEWYEMLMGHCTYFPEELRSVKYAYNADLYNALND
LNNLVITRDENEKLEYYEKFQIIENVFKQKKKPTLKQIAKEILVNEEDIKGYRVTSTGKPEFTNL
KVYHDIKDITARKEIIENAELLDQIAKILTIYQSSEDIQEELTNLNSELTQEEIEQISNLKGYTG
THNLSLKAINLILDELWHTNDNQIAIFNRLKLVPKKVDLSQQKEIPTTLVDDFILSPVVKRSFIQ
SIKVINAIIKKYGLPNDIIIELAREKNSKDAQKMINEMQKRNRQTNERIEEIIRTTGKENAKYLI
EKIKLHDMQEGKCLYSLEAIPLEDLLNNPFNYEVDHIIPRSVSFDNSFNNKVLVKQEENSKKGNR
TPFQYLSSSDSKISYETFKKHILNLAKGKGRISKTKKEYLLEERDINRFSVQKDFINRNLVDTRY
ATRGLMNLLRSYFRVNNLDVKVKSINGGFTSFLRRKWKFKKERNKGYKHHAEDALIIANADFIFK
EWKKLDKAKKVMENQMFEEKQAESMPEIETEQEYKEIFITPHQIKHIKDFKDYKYSHRVDKKPNR
ELINDTLYSTRKDDKGNTLIVNNLNGLYDKDNDKLKKLINKSPEKLLMYHHDPQTYQKLKLIMEQ

YGDEKNPLYKYYEETGNYLTKYSKKDNGPVIKKIKYYGNKLNAHLDITDDYPNSRNKVVKLSLKP
YRFDVYLDNGVYKFVTVKNLDVIKKENYYEVNSKCYEEAKKLKKISNQAEFIASFYNNDLIKING
ELYRVIGVNNDLLNRIEVNMIDITYREYLENMNDKRPPRIIKTIASKTQSIKKYSTDILGNLYEV
KSKKHPQIIKKGSGGSTNLSDIIEKETGKQLVIQESILMLPEEVEEVIGNKPESDILVHTAYDES
TDENVMLLTSDAPEYKPWALVIQDSNGENKIKMLSGGSPKKKRKV*
